# Early effect of laser irradiation in signaling pathways of diabetic rat submandibular salivary glands

**DOI:** 10.1371/journal.pone.0236727

**Published:** 2020-08-04

**Authors:** Cíntia Yuki Fukuoka, Hugo Passos Vicari, Carla Renata Sipert, Ujjal Kumar Bhawal, Yoshimitsu Abiko, Victor Elias Arana-Chavez, Alyne Simões

**Affiliations:** 1 Laboratory of Oral Biology, Department of Biomaterials and Oral Biology, School of Dentistry, University of São Paulo, São Paulo, Brazil; 2 Division of Endodontics, Department of Restorative Dentistry, School of Dentistry, University of São Paulo, São Paulo, Brazil; 3 Department of Biochemistry and Molecular Biology, Nihon University School of Dentistry at Matsudo, Matsudo, Chiba, Japan; National Institutes of Health, UNITED STATES

## Abstract

Low-power laser irradiation (LPLI) is clinically used to modulate inflammation, proliferation and apoptosis. However, its molecular mechanisms are still not fully understood. This study aimed to describe the effects of LPLI upon inflammatory, apoptotic and proliferation markers in submandibular salivary glands (SMGs) in an experimental model of chronic disorder, 24h after one time irradiation. Diabetes was induced in rats by the injection of streptozotocin. After 29 days, these animals were treated with LPLI in the SMG area, and euthanized 24h after this irradiation. Treatment with LPLI significantly decreased diabetes-induced high mobility group box 1 (HMGB1) and tumor necrosis factor alpha (TNF-α) expression, while enhancing the activation of the transcriptional factor cAMP response element binding (CREB) protein. LPLI also reduced the expression of bax, a mitochondrial apoptotic marker, favoring the cell survival. These findings suggest that LPLI can hamper the state of chronic inflammation and favor homeostasis in diabetic rats SMGs.

## Introduction

Photobiomodulation therapy (PBMT) using low-power laser irradiation (LPLI) is a promising treatment for inflammatory disorders and biomodulation processes. Clinically it shows great results in Sjögren syndrome, oral mucositis and rheumatoid arthritis treatment by its effects upon the biomodulation of the inflammation and tissue repair processes [1–3].

Molecular studies indicate that LPLI can decrease the expression of many inflammatory markers, as the high mobility group box 1 (HMGB1) and the tumor necrosis factor alpha (TNF-α) [4–6]. *In vitro* and *in vivo* studies also show the laser effects upon proliferation and apoptosis [5, 7], increasing the expression of many growth factors [4].

Diabetes is a disease characterized by chronic hyperglycemia that results in damage in many organs [8]. It increases the formation of advanced glycation end-products (AGEs) [9, 10], activating the receptor for advanced glycation end-products (RAGE) and self-sustaining the inflammation by up-regulation of the nuclear factor kappa-light-chain-enhancer of activated B cells (NFκB) [11]. Clinical reports revealed high levels of HMGB1, a high-affinity ligand of RAGE, and enhanced NFκB activity in the blood of diabetic patients [12, 13].

Diabetes also impairs the balance between proliferation and apoptosis [9, 10]. Studies in cutaneous tissue repair after injury in diabetic animals, showed a delay in the reepithelialization process, with lack of growth factors and less angiogenesis [14]. The increased inflammatory markers and AGEs can also lead to apoptosis by activation of RAGE, resulting in the cleavage of caspase-3 and cell death [15, 16].

Apoptosis is an important event responsible for the tissue homeostasis that occurs mainly by the extrinsic and the intrinsic pathways. Basically, the extrinsic pathway is mediated by death receptors in the surface of the cell external membrane. The intrinsic pathway, also known as mitochondrial pathway, occurs by the interaction of pro-apoptotic proteins such as, bax and bad, with caspases, both culminating with the activation of caspase-3 leading to cell death [17]. The inflammatory mediator, TNF-α can induce apoptosis by the two pathways [18, 19]. The HMGB1 protein, by the other hand, is a redox sensitive regulator of the cell fate. Under conditions of severe metabolic stress, intracellular HMGB1 controls apoptosis and autophagy, an event that degrades damaged organelles and defective proteins in intracellular vacuoles [20, 21]. Extracellular HMGB1 can promote inflammation, and activate autophagy or intrinsic apoptotic pathways, depending on its interaction with its receptors in the cell membrane surface [20, 22]. The process of inflammation and apoptosis is, therefore, closely- related and stringently controlled by many molecules.

In salivary glands, diabetes impairs its function and alters its metabolism [8, 23, 24]. Increases autophagy and lysosomal activity, as well as, endocytosis of proteins in ducts cells, decreases the salivary flow and reduces the total amount of sialic acid in the saliva, which might be related to the xerostomia reported by diabetic patients [25–27]. Morphologically, diabetes salivary glands presents lipid droplets accumulation and signs of degeneration of salivary secretory and ducts cells, with substitution by connective tissue [26, 28].

Previous reports from our group indicate that LPLI can modulate salivary glands metabolism activity and function [23, 24, 29]. A recent finding showed reduction of lipid droplets in LPLI- treated diabetic rats parotid glands, thus favoring its normal function [30].

Since LPLI-induced effects can be mediated by cyclic adenosine monophosphate (cAMP) [31], we investigated its pathway in diabetic SMG. It is well known that cAMP response element binding (CREB) protein, is a transcription factor that regulates diverse cellular responses, including cell proliferation and survival [32]. Recent evidences indicated that LPLI beneficial effects might be related to the regulation of CREB pathway [33, 34].

To further elucidate the potential mechanism of LPLI in biomodulation, we investigated its early effects on the cAMP/CREB signaling pathway, as well as, upon inflammatory and apoptotic markers in submandibular salivary glands.

## Materials and methods

### Animals and diabetes induction

Female Wistar rats (n = 24) (*Rattus novergicus*) weighing 115–221 g, were divided in three experimental groups (n = 8), C0, D0 and D20, fed with a standard laboratory diet. Diabetes was induced in groups D0 and D20, by intraperitoneally injecting adult rats with streptozotocin, single dose of 60 mg/kg, dissolved in citrate buffer (100 mM; pH 4.5), in the first experimental day. After 3 days, blood glucose of 12h-fasting rats was measured and animals presenting glycemic levels equal or above 14 mM were considered diabetics. Naïve control rats, group C0, received only the injection of citrate buffer (100 mM, pH 4.5). On the 29^th^ experimental day, all animals were anesthetized with ketamine/xylazine solution (4 ml/kg IM), but only group D20 received the low-level laser irradiation, according to Simões et al. [23]. Twenty-four hours after the irradiation, all the animals were anesthetized, the 12h-fasting glycemia was measured, the SMG were removed and the animals were decapitated (S1 Fig). This animal experiment was performed in the same way as reported by Fukuoka *et al*. [5]

All experiments were performed in accordance with the ARRIVE guidelines and were carried out in accordance with the U.K. Animals (Scientific Procedures) Act, 1986 and associated guidelines, EU Directive 2010/63/EU for animal experiments, and the National Institutes of Health guide for the care and use of Laboratory animals (NIH Publications No. 8023, revised 1978), approved by the Ethics Committee of Animal Experimentation of the University of São Paulo School of Dentistry, Brazil (Protocol 002/2019-CEUA).

### Low-power laser irradiation procedure

For this procedure, first we anesthetized and made the trichotomy in the rats SMG area. After that, we drawn a circle with 1.13 cm^2^ (above the area of both SMG together). Inside this area, LPLI was applied in 40 points, transcutaneous and perpendicular to the skin surface, in contact with it, but without pressure (S1 Fig).

The wavelength used was 660-nm continuous (70 mW; laser spot area of 0.028 cm^2^; Photon Lase III, DMC IND. CO., Ltd., São Paulo, Brazil). Forty points of irradiation with 0.56 J, 8 seconds per point was performed at energy densities of 20 J/cm^2^, covering the SMG area, 1.13 cm^2^, being the total energy of 22.4 J and laser spot area of 0.028 cm^2^, as described by Simões *et al*. [23] modified.

### SMG immunohistochemistry analysis

Immunostaining was performed on 4% formaldehyde fixed and embedded paraffin-blocks, cut into 4μm sections. For inflammatory markers, rabbit monoclonal anti-HMGB1 (1:250; Abcam, Inc., Cambridge, MA, USA, Cat# ab79823, RRID:AB_1603373), rabbit polyclonal TNF-α (IHC World, LLC, Woodstock, MD, Cat #IW-PA1079), rabbit polyclonal anti-RAGE (1:20; GeneTex, Inc., Irvine, CA, USA, Cat# GTX23611, RRID:AB_385109) and rabbit polyclonal anti-phosphorylated NFκB p65 (1:100; Bioss, Inc., Woburn, MA, USA, Bioss, Cat# bs-0982R, RRID:AB_10856012) were used as the primary antibodies. And for proliferation markers, rabbit polyclonal anti-cAMP (1:100; EDM Millipore, Corp., Billerica, MA, Cat# 07–1497, RRID:AB_10616218), rabbit monoclonal anti-phospho-p44/42 MAPK (ERK1/2) (1:200; Cell Signaling Technology, Inc., Danvers, MA, Cat# 4370, RRID:AB_2315112), rabbit monoclonal anti-phospho-CREB antibody (detecting CREB phosphorylated at Ser-133) (1:250, Abcam, Inc., Cambridge, MA, USA, Cat# ab32096, RRID:AB_731734) and rabbit monoclonal anti-cyclin D1 (1:100; Abcam, Inc., Cambridge, MA, USA, Cat# ab134175, RRID:AB_2750906) were used as the primary antibodies. All primary antibodies were incubated overnight at 4°C, according to the manufacturer’s instructions. The specimens were rinsed in PBS, and incubated with the secondary antibody Histofine Simple Stain Rat MAX-PO (Multi) (Nichirei Biosciences, Tokyo, Japan), for 1h at room temperature. The reaction was visualized with DAB solution (DAKO, North America Inc. Carpinteria, CA, USA). The slides were counterstained with hematoxylin, dehydrated and coverslipped.

### SMG western blotting analysis

Tissues were kept in RNA Later (Life Technologies), and homogenized in ice-cold RIPA lysis buffer (Santa Cruz Biotechnology, Santa Cruz, CA, USA) and then centrifuged at 15000 rpm for 15 min at 4°C. Bicinchoninic Acid (BCA) protein assay kit (Pierce Biotechnology, Rockford, IL, USA) was utilized to measure the protein concentration in the supernatant. Equal protein amounts were used for Western blot using the following antibodies: rabbit monoclonal anti-HMGB1 (1:800; Abcam, Cambridge, MA, USA, Cat# ab79823, RRID:AB_1603373), rabbit polyclonal anti-RAGE (1:500; GeneTex, Inc., Irvine, CA, USA, Cat# GTX23611, RRID:AB_385109), rabbit polyclonal anti-phosphorylated NFkB p65 (1:200; Bioss, Inc., Woburn, MA, USA, Bioss Cat# bs-0982R, RRID:AB_10856012), rabbit monoclonal phospho-p44/42 MAPK (ERK1/2) (1:1000; Cell Signaling Technology, Inc., Danvers, MA, Cat# 4370, RRID:AB_2315112), rabbit monoclonal total p44/42 (ERK) (1:1000, Cell Signaling, Cat# 4695, RRID:AB_390779), rabbit monoclonal anti-cyclin D1 (1:200; Abcam, Cambridge, MA, USA, Cat# ab134175, RRID:AB_2750906), rabbit monoclonal anti-phosphorylated CREB (ser 133) (1:200; Abcam, Cambridge, MA, USA, Abcam Cat# ab32096, RRID:AB_731734), rabbit monoclonal anti-bax (1:1000; Abcam, Cambridge, MA, USA, Cat# ab32503, RRID:AB_725631), rabbit polyclonal anti-cleaved caspase-3 (Asp 175) (1:200; Cell Signaling Technology, Danvers, MA, USA, Cat# 9661, RRID:AB_2341188) and rabbit monoclonal anti-β-actin (Cell Signaling Technology, Inc., Danvers, MA Cat# 4970, RRID:AB_2223172). The membrane was incubated with horseradish peroxidase-conjugated anti-rabbit secondary antibody (Cell Signaling Technology Inc., Danvers, MA Cat# 7074, RRID:AB_2099233) for 1 h at room temperature. Blots were developed by ECL Plus Western Blotting Detection System (Amersham, Uppsala, Sweden), and images were analyzed with a Luminescent Image Analyzer (G:BOX XRQ; Syngene, MD, USA, https://www.syngene.com/software/genesys-rapid-gel-image-capture/). The relative expression of each protein vs. β-actin expression was measured using National Institutes of Health Image J software (http://imagej.nih.gov/ij/).

### SMG qRT-PCR analysis

After removal, SMG samples were kept in RNA Later (Life Technologies). Total RNA was extracted using RNeasy Mini Kit (Qiagen, Hilden, Germany) and the RNA concentrations were determined with Nanodrop (Thermo Fisher Scientific Inc., Paisley, PA, RU). Reverse transcription for cDNA synthesis was performed with 1 μg of each total RNA using Super Script VILO Master Mix (Life Technologies, CA, USA). One μl of each cDNA was subjected to real-time quantitative polymerase chain reaction (qPCR), using TaqMan Master Mix together with TaqMan Gene Expression Assays for HMGB1 (Rn02377062_g1), RAGE (Rn01525753_g1), CREB5 (Rn01451883_m1) and β-actin (Rn01412977_g1) (Applied Biosystems, Framingham, MA, USA), performed on Step One Plus equipment (Thermo Fisher Scientific, MA, USA). Standard thermocycling conditions were used in a total volume of 10 μl. β-actin, was used as an endogenous control gene. Fold change in intensity of the target gene was calculated using Step One Software version 2.3 (https://www.thermofisher.com/br/en/home/technical-resources/software-downloads/StepOne-and-StepOnePlus-Real-Time-PCR-System.html) based on the control group.

### Double immunofluorescence for phospho ERK and phospho CREB in SMG

Paraffin sections of the SMG (4-μm thick) were deparaffinized in xylene and rehydrated in graded alcohol series. Sections were immersed in citrate buffer pH 6.0 (Epitomics) at 97°C for 20 min, washed in PBS and immersed in peroxidase blocking (DAKO) for 10 min. The samples were then washed in PBS and incubated in protein block (DAKO) for 1 h. The first antibody phosphorylated ERK (1:150, Cell Signaling Technology, Cat# 4370, RRID:AB_2315112) was prepared with Zenon Alexa Fluor 594 (Molecular Probes, Inc., Eugene, OR) according to manufacturer’s instructions, and applied to the samples and incubated in a humidified dark chamber for 2 h. Then the sections were washed in PBS for 25 min and subsequently incubated with the second antibody phosphorylated CREB (1:150, Abcam Cat# ab32096, RRID:AB_731734) prepared with Zenon Alexa Fluor 488 for 2 h. DAPI was used for nuclear counterstaining.

### SMG apoptosis analysis with terminal deoxynucleotidyl transferase-mediated dUTP nick end-labelling (TUNEL) assay

The paraffin-embedded SMG tissues were sectioned and mounted on glass slides. The slides were de-paraffinized and dehydrated, treated with proteinase K 1:100 for 15 min at room temperature. Slides were then immersed in peroxidase blocking (DAKO) for 5 min, followed by washing with PBS. Next, slides were incubated in 1XTdT Labeling Buffer for 5 minutes, and covered with 50 μl of Labeling Reaction Mix at 37°C for 1 h. Sections were then immersed in 1XTdT Stop Buffer for 5 min and washed in PBS. To visualize TUNEL-positive cells, slides were staining with 3, 3′-diaminobenzidine (DAB). The samples were later washed, counterstained with 1% methyl green for 5 min before washing. All these process were performed according to the manufacturer’s instructions (Trevigen, Inc., Gaithersburg, MD, USA).

After the image acquisition, we quantified the positive staining by image colour deconvolution in Image J. First, the image was opened into the program and set the command: *Analyze/Set scale* to calibrate all the images with the scale bar. Then we measure the whole area by selecting it and add the command: *Analyze/Measure* to obtain the total area. After that the image was deconvoluted by color: *Plugins/Colour deconvolution/HDAB*. Only the brown color image was selected and set to 8-bit (*Image/Type/8-bit*), the threshould was stablished (*Image/Adjust/Threshould*) and the area with positive staining was calculated by *Analyze/Analyze particles*. We obtained the whole image area and the percentage of the area with positive staining, as described by Castro [35].

### Enzyme-Linked Immunosorbent Assay (ELISA) for HMGB1 and TNF-α in SMG

The concentrations of HMGB1 and TNF-α in the protein samples extracted from SMGs, were evaluated using the HMGB1 Rat Elisa kit (MyBiosource, Inc., San Diego, CA) and Legend Max Rat TNF-α ELISA kit (Biolegend, Inc., San Diego, CA) respectively, according to the manufacturer’s instructions. The total protein concentrations in SMGs samples were determined with BCA protein assay kit (Pierce Biotechnology, Rockford, Il, USA) following the protocol provided with the kit.

### Statistical analysis

Data was presented as mean ± SEM. Statistical analysis was performed using ANOVA with Tukey. Differences were considered to be significant at P<0.05. Minitab 19 software (https://www.minitab.com/pt-br/products/minitab/) was used for statistical calculations.

## Results

### Metabolic parameters

At the end of the experiment, D0 animals have lost about 22% of their initial body weight and D20 animals lost 26% of their weight (P < 0.05) (S1 Table). No changes were observed in C0 group.

The initial fasting blood glucose was about 4 times higher in the diabetic groups (P < 0.05), in relation to the C0 group. The LPLI significantly reduced 22% of the diabetic rats glycemia (S1 Table).

### HMGB1 and RAGE mRNA expression in SMG

**Fig 1:**
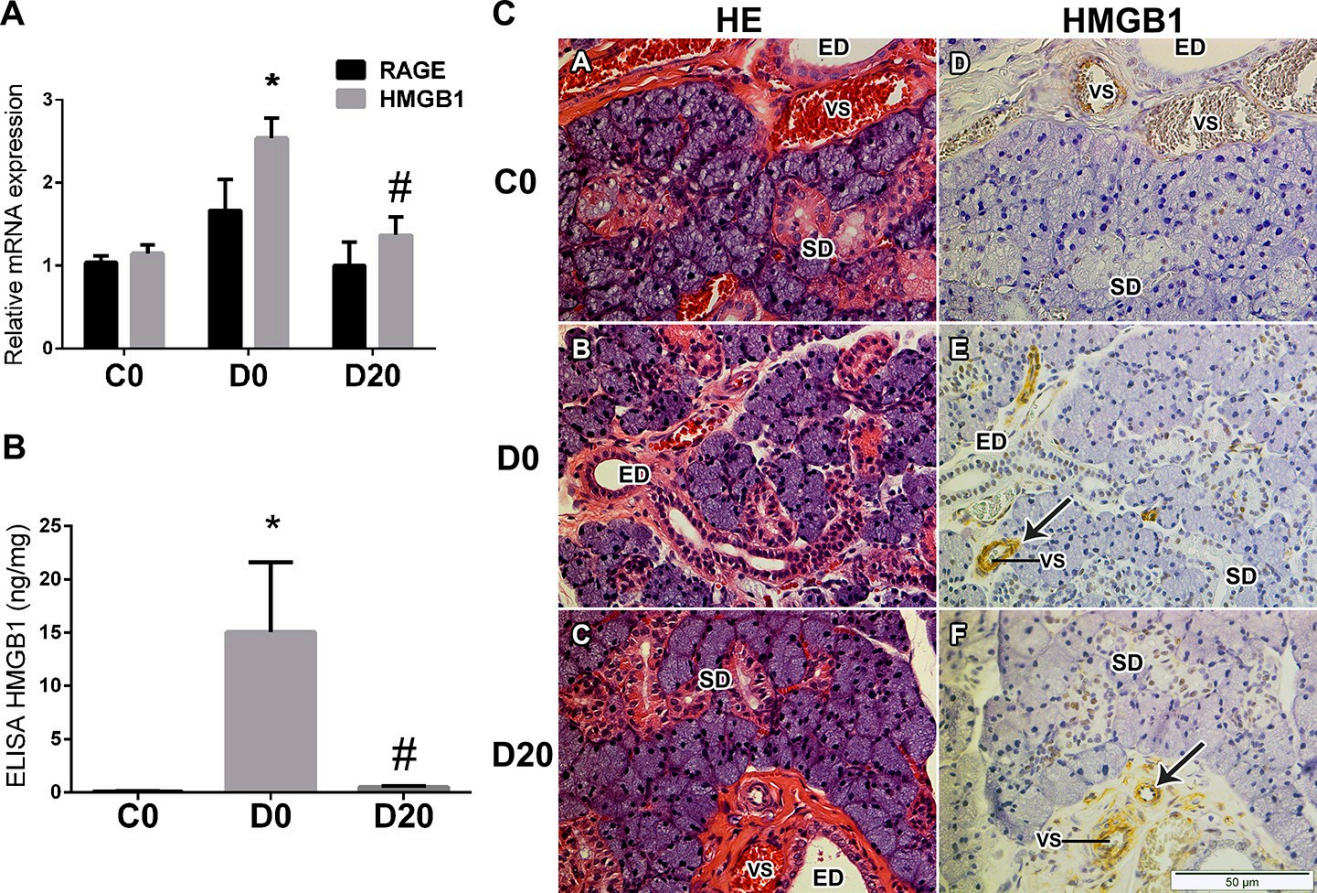
LPLI reduced HMGB1 expression in diabetic rats SMG. A. RAGE and HMGB1 mRNA expression were analyzed by qRT-PCR (n = 4/group). B. ELISA analysis for HMGB1 in ng/mg of total protein. From left to right: control (C0 n = 4), diabetes (D0 n = 5) and diabetes treated with LPLI (D20 n = 5), respectively. Bar graphs indicate means ± SE; * P < 0.05 vs control group (C0) and # P < 0.05 vs respective diabetic group (D0). C. Hematoxylin and eosin (HE) staining shows a normal morphology of acinar and ducts cells in all groups, C0, D0 and D20 (n = 3). Immunohistochemistry analysis for HMGB1 in SMG (n = 3). Arrows indicate the positive staining for HMGB1 in endothelial cells. Scale bar, 50μM. ED, excretory duct; SD, striated duct; VS, blood vessel.

Diabetes led to a 2.2-fold increase on HMGB1 mRNA levels in D0 rats SMG (P = 0.002). LPLI decreased diabetic rats SMG transcription of HMGB1 to levels similar to control ones, mean of 1.15 ± 0.10, 2.54 ± 0.24, 1.37 ± 0.22 for C0, D0 and D20, respectively. The levels of RAGE mRNA did not differ between the groups, mean of 1.04 ± 0.08 for C0, 1.67 ± 0.37 for D0 and 1.00 ± 0.29 for D20 (Fig 1A).

Our findings showed increased HMGB1 protein levels in the SMG of D0 group in relation to C0 group, means of 15.06 ± 13.70 ng/mg of protein and 0.09 ± 0.02 ng/mg of protein for D0 and C0, respectively (P = 0.008). LPLI reduced these levels of HMGB1 in the diabetic rats in 96.60%, to the mean value of 0.50 ± 0.24 ng/mg of total protein in D20, similar to the control group (C0) (Fig 1B).

Although SMG morphology does not differ much between groups, diabetic animals SMG glands seems to have increased connective tissue areas with more space between acinus (Fig 1C). HMGB1 was localized mainly in the nucleus of endothelial cells in the diabetic animals SMG, as well as, in the surrounding perivascular connective tissue (Fig 1C- section 1E and 1F). The presence of HMGB1 staining was also observed in the nucleus of ducts cells, striated and excretory ducts, in all groups (Fig 1C).

### TNF-α expression in SMG

**Fig 2:**
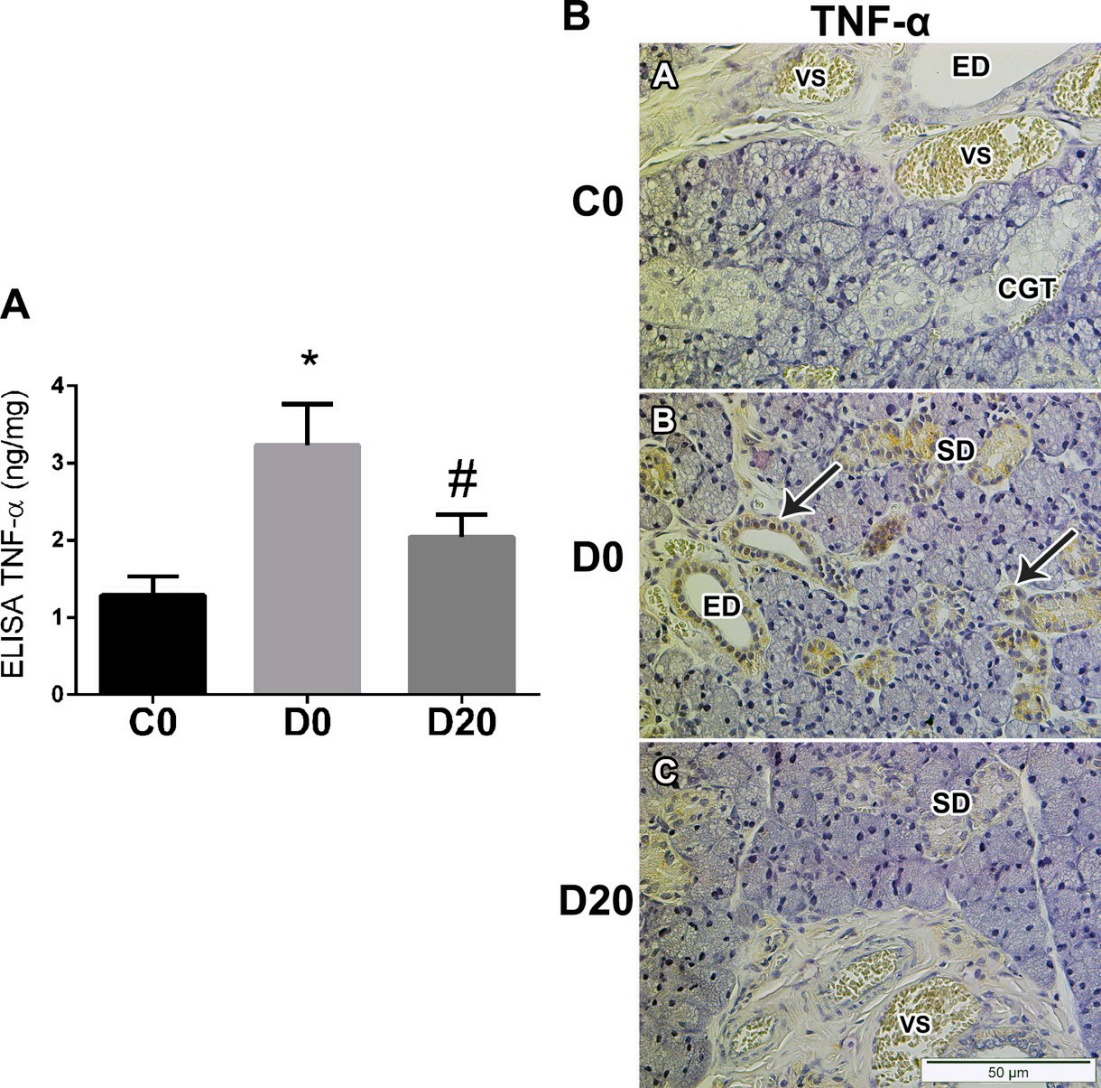
LPLI reduced TNF-α expression in diabetic rats SMG. A. ELISA analysis for TNF-α in pg/mg of total protein (n = 6). From right to left: control (C0), diabetes (D0) and diabetes treated with LPLI (D20). Bar graphs indicate means ± SE; * P < 0.05 vs control group (C0) and # P < 0.05 vs respective diabetic group (D0). B. Immunohistochemistry analysis for TNF-α in SMG (n = 3). Arrows show positive staining for TNF-α in striated and excretory ducts cells nucleus and cytoplasm. Scale bar, 50μM. ED, excretory duct; SD, striated duct; CGT, convoluted granular tubules; VS, blood vessel.

The levels of TNF-α in the diabetic animals (D0 group) showed an increase of 2.50 times in relation to control group (C0), means of 1.28 ± 0.60 pg/mg and 3.23 ± 1.20 pg/mg of total protein for C0 and D0, respectively (P = 0.038). After LPLI, these levels reduced 36.84% when compared to D0 group, mean of 2.04 ± 0.70 pg/mg in D20, similar to the values founded in the control group (Fig 2A).

In the immunohistochemistry analysis, staining for TNF-α was not detected in the C0 group (Fig 2B- section 2A). However, the presence of TNF-α was observed in some nucleus and cytoplasm of ducts cells, striated and excretory, as well as in the connective tissue in D0 group (Fig 2B- section 2B). A slight staining was founded in the cytoplasm of ducts cells in D20 group (Fig 2B- section 2C).

### RAGE and phosphorylated NFκB protein expression in SMG

**Fig 3:**
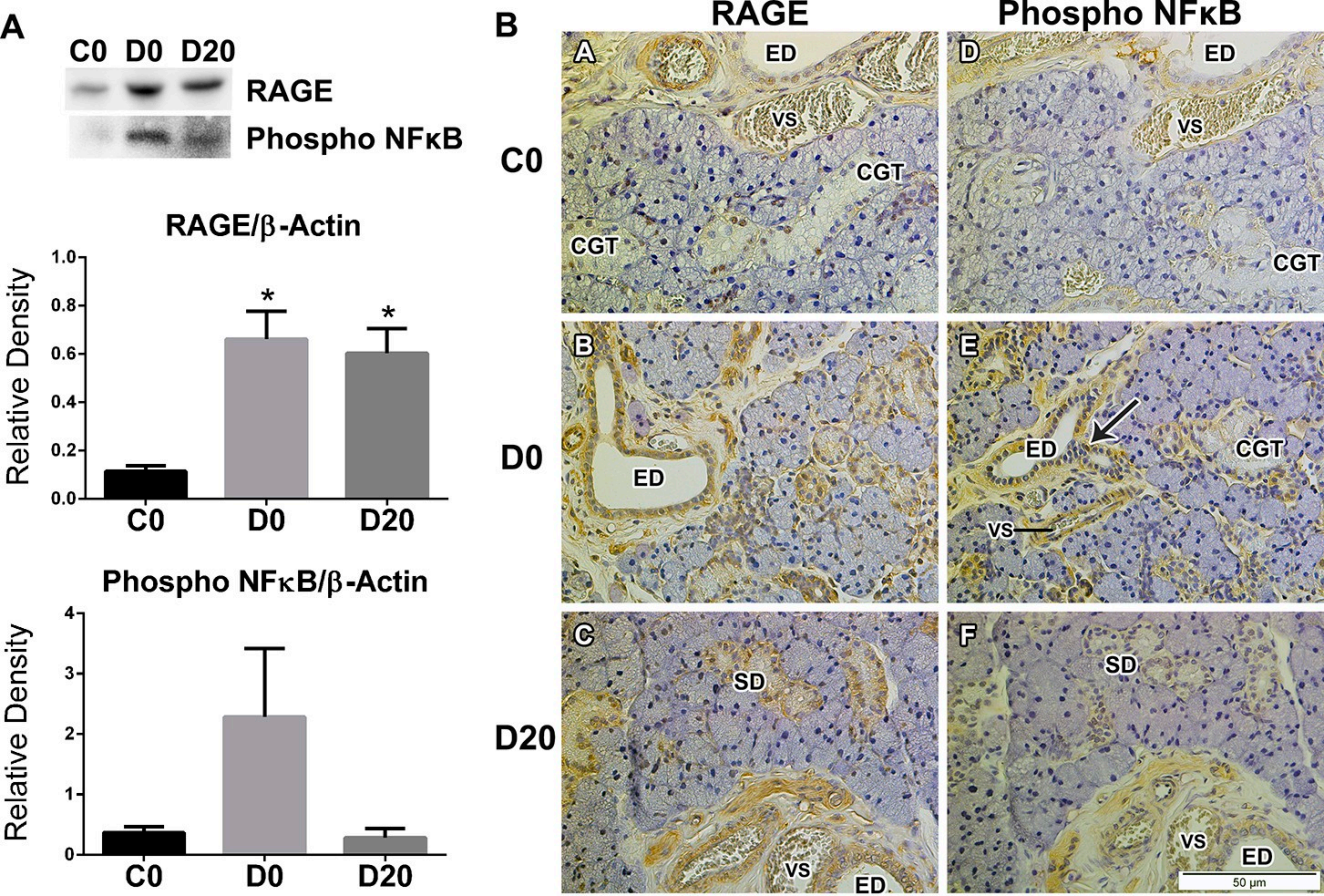
LPLI effects upon NFκB activation in diabetic rats SMG. A. Western blot analysis for RAGE (n = 7) and phosphorylated NFκB (n = 4). The bands were detected and quantified using an image analyzer, and RAGE and NFκB protein levels were normalized to that of β-actin. Protein sizes were evaluated by standard protein markers, and their sizes were as follows: RAGE (45 kDa) and phosphorylated p65 (65 kDa). From left to right: control (C0), diabetes (D0) and diabetes treated with LPLI (D20). Bar graphs indicate means ± SE; * P < 0.05 vs control group (C0). B. Immunohistochemistry analysis for RAGE and NFκB in SMG (n = 3). Arrows show the positive staining in the nucleus of excretory ducts cells and perivascular connective tissue cells. Scale bar, 50μM. ED, excretory duct; SD, striated duct; CGT, convoluted granular tubules; VS, blood vessel.

Diabetes significantly increased RAGE expression in SMG when compared to control group, mean of 0.11 ± 0.05 for C0, 0.66 ± 0.30 for D0 and 0.60 ± 0.26 for D20 (P = 0.001). There were no significant changes after LPLI (Fig 3A).

The expression of the transcriptional factor, phosphorylated NFκB had a tendency to increase in diabetic samples and decrease with LPLI, means of 0.36 ± 0.18, 2.28 ± 2.26 and 0.28 ± 0.29, for C0, D0 and D20, respectively (Fig 3A).

The localization of RAGE in SMG was observed in acinar and ducts cells, striated, excretory and convoluted granular tubules, as well as, in the perivascular connective tissue in all groups (Fig 3B). The activated p65 was founded in the nucleus of ducts and in the perivascular connective tissue, mainly stained in the groups D0 and D20 (Fig 3B- section 3E and 3F).

### cAMP and ERK expression in SMG

**Fig 4:**
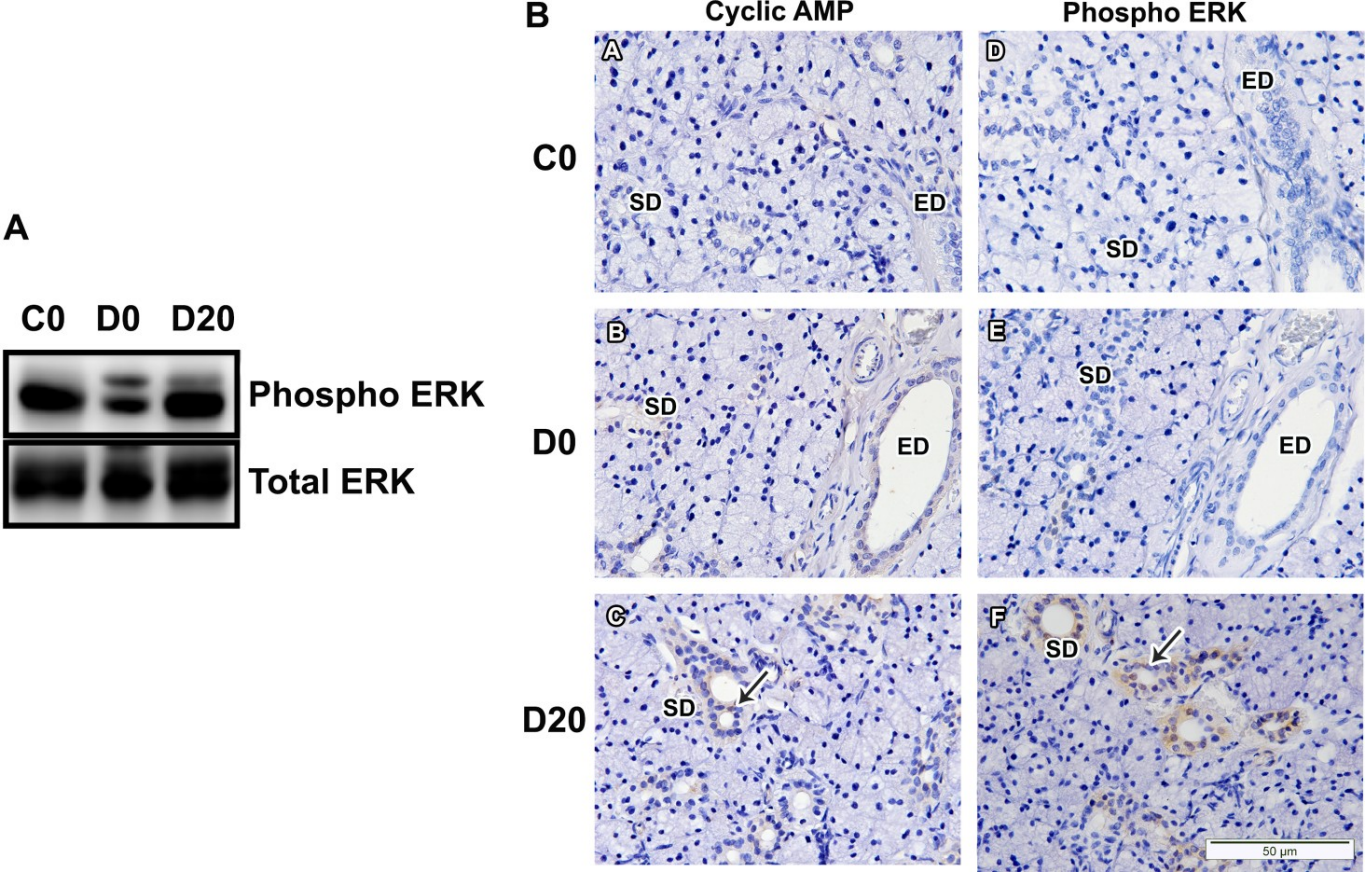
LPLI effects upon cAMP and ERK expression. A. Western blotting analysis for phosphorylated ERK and total ERK. From left to right: control (C0), diabetes (D0) and diabetes treated with LPLI (D20), respectively. Protein sizes were evaluated by standard protein markers, and their sizes were as follows: phosphorylated ERK (42, 44 kDa) and total ERK (42, 44 kDa) (n = 1/group). B. Immunohistochemistry analysis for cAMP and phosphorylated ERK in SMG. Arrows points to positive-stained striated ducts cells in LPLI treated group (n = 1/group). Scale bar, 50μM. ED, excretory duct; SD, striated duct. B. Western blotting analysis for phosphorylated ERK and total ERK. From left to right: control (C0), diabetes (D0) and diabetes treated with LPLI (D20), respectively. Protein sizes were evaluated by standard protein markers, and their sizes were as follows: phosphorylated ERK (42, 44 kDa) and total ERK (42, 44 kDa) (n = 1/group).

Our results indicate that LPLI might induce cAMP expression, in acinus, ducts, endothelium and myofibroblasts cells, stained in D20 group (Fig 4B- section 4C). To further analyze expression of components of the cAMP signaling cascade, we checked ERK expression. ERK activation seems to be hampered in the diabetic submandibular glands (Fig 4A). In contrast, laser irradiation might enhance ERK phosphorylation, predominantly in ducts cells (Figs 4B- section 2F).

### CREB expression in SMG

**Fig 5:**
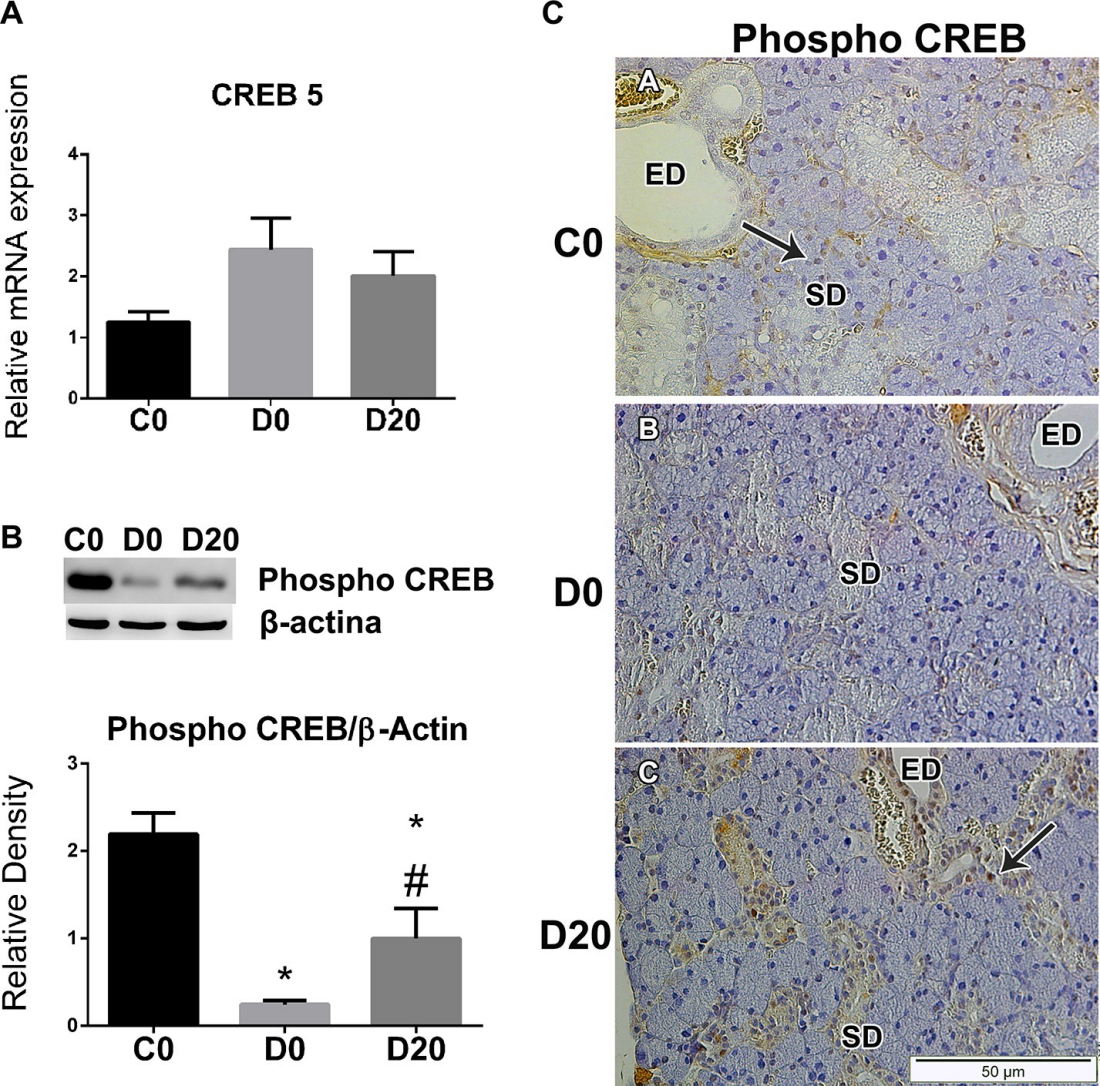
LPLI triggered the activation of the transcriptional factor CREB. A. CREB5 mRNA expression in SMG analyzed by qRT-PCR (n = 4/group). B. Western blotting analysis for phosphorylated CREB (Ser 133) and β-actin. From left to right: control (C0), diabetes (D0) and diabetes treated with LPLI (D20), respectively. Protein sizes were evaluated by standard protein markers, and their sizes were as follows: phosphorylated CREB (36 kDa) and β-actin (42 kDa) (n = 6/group). Bar graphs indicate means ± SE; * P < 0.05 vs control group (C0); # P < 0.05 vs respective diabetic group (D0). C. Immunohistochemistry analysis for phosphorylated CREB in SMG. Arrows points to positive-stained striated ducts cells (n = 3/group). Scale bar, 50μM. ED, excretory duct; SD, striated duct.

Our results suggest that diabetes does not interfere in the transcription of the cAMP responsive element binding protein 5, CREB5, in SMG, and LPLI did not change its mRNA levels expression, means of 1.20 ± 0.34, 2.44 ± 1.00 and 2.01 ± 0.79 for C0, D0 and D20, respectively (Fig 5A).

In spite of this finding, diabetes reduced the expression of phosphorylated CREB (Ser 133) to 10.95% of the values found in control, means of 2.19 ± 0.58 for C0 and 0.24 ± 0.11 for D0. In turn, LPLI significantly (P = 0.000) enhanced the activation of this transcriptional factor in SMG of the treated diabetic rats, mean of 0.99 ± 0.83 in D20 (Fig 5B).

Phosphorylated CREB could be observed in all cell types, acinus, ducts, endothelium, in control animals, but mainly in the nucleus of striated and excretory ducts cells, strongly stained in C0 and D20 groups (Fig 5C- section 5A and 5C).

### CREB and ERK co-localization in SMG

Since phosphorylated CREB and ERK can interact with each other, we performed double immunofluorescence and found double positives cells in laser-irradiated diabetic submandibular glands, endothelium and ducts cells (S2G Fig and S2I Fig), while almost no expression was seen in diabetes (S2H Fig).

### Apoptotic markers in SMG

**Fig 6:**
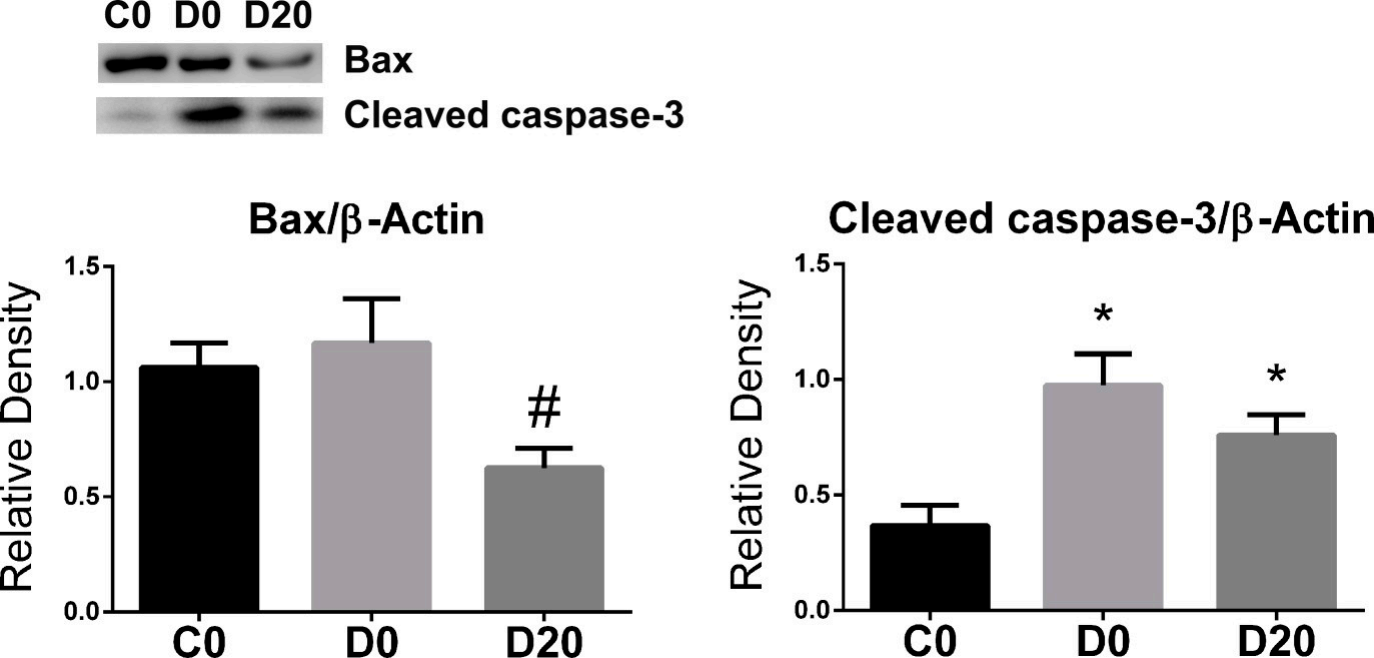
LPLI decreased cell death marker. Western blotting analysis of apoptotic markers, bax (n = 8/group) and cleaved-caspase-3 (n = 7/group), 21 kDa and 17–19 kDa respectively. From left to right: control (C0), diabetes (D0) and diabetes treated with LPLI (D20), respectively. Data are expressed as mean ± SE; * P < 0.05 vs control group (C0); # P < 0.05 vs respective diabetic group (D0).

Diabetes seems to induce apoptosis via activation of the caspase-3 pathway (P = 0.003), mean of 0.97 ± 0.31 for D0, 0.75 ± 0.24 for D20 and 0.36 ± 0.24 for C0 (Fig 6), without changes in bax expression in SMG, mean of 1.06 ± 0.29 for C0 and 1.16 ± 0.54 for D0 (Fig 6). LPLI could down-regulate the expression of bax, a mitochondrial apoptotic marker, mean of 0.62 ± 0.23 in D20 group (P = 0.023), but not activated caspase-3.

**Fig 7:**
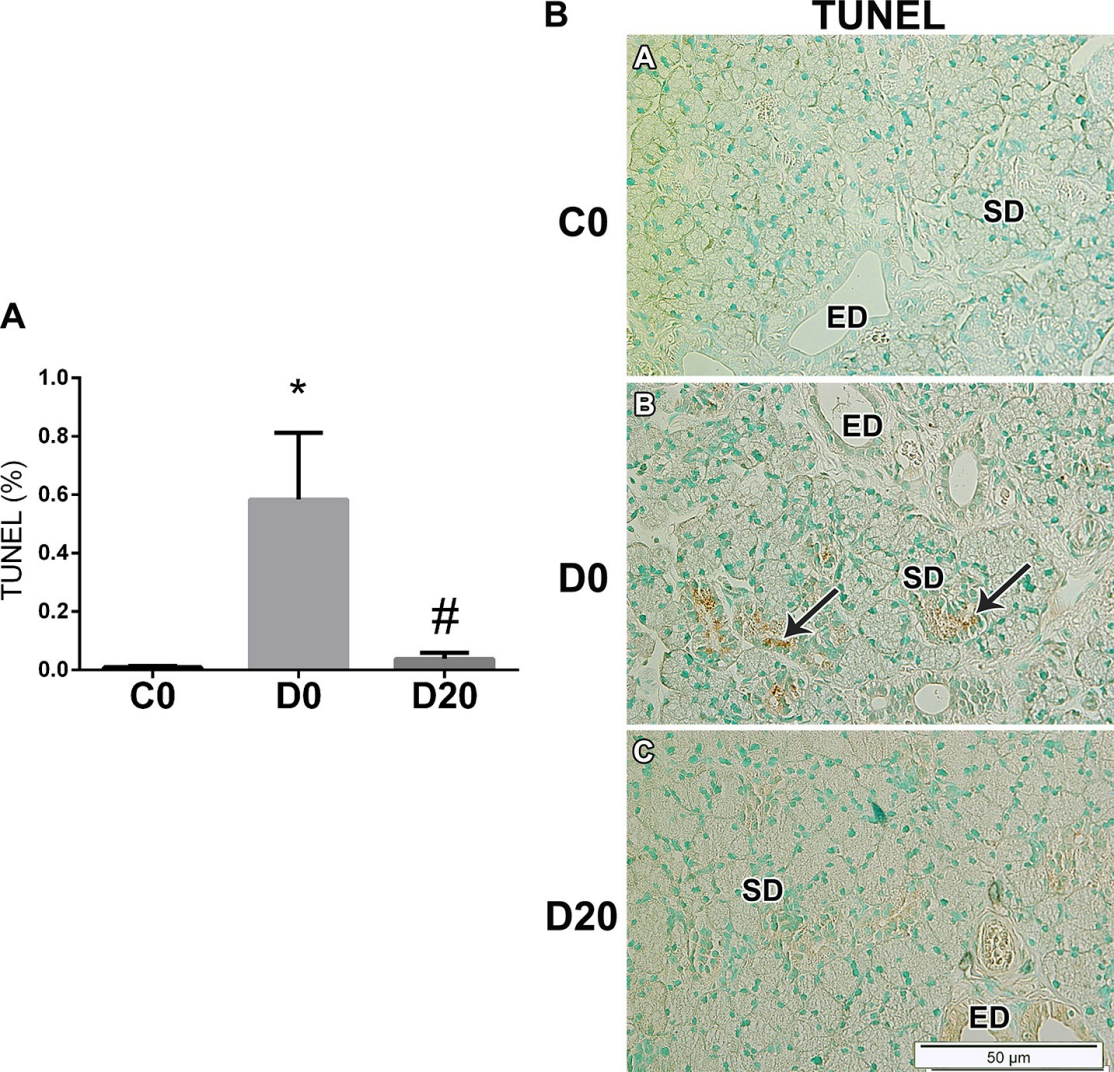
LPLI reduces apoptosis in diabetic SMG. A. The images for TUNEL assay were quantified by color deconvolution, and shows the percentage of positive staining in relation to the whole image area (n = 4/group). From left to right: control (C0), diabetes (D0) and diabetes treated with LPLI (D20), respectively. Data are expressed as mean ± SE; * P < 0.05 vs control group (C0); # P < 0.05 vs respective diabetic group (D0). B. Detection of apoptosis by TUNEL assay in SMG (n = 3/group). Arrows indicate positive-staining in the nucleus of striated ducts cells. Scale bar, 50μM. ED, excretory duct; SD, striated duct.

TUNEL assay shows positive-marked cells mainly in striated ducts, mainly in D0 samples (Fig 7B- section 7B). The quantification of apoptotic cells by image deconvolution indicates an increase in apoptotic cells in diabetic rats SMG, and a significant reduction after LPLI (P = 0.022), to levels similar to control group (Fig 7A).

## Discussion

The current study investigated the early mechanism of action of LPLI in inflammation, apoptosis and proliferation pathways of salivary glands. As expected, diabetes significantly increased inflammatory markers such as, HMGB1 and TNF-α in SMG, as well as the receptor RAGE with a tendency of increase in the activation of the transcriptional factor NFκB. LPLI reduced HMGB1 and TNF-α expression to similar levels to the ones found in control group. These findings are in accordance to our previous study, where we suggested these effects by western blotting analysis for HMGB1 and immunohistochemistry for HMGB1 and TNF-α in diabetic rats SMG [5]. Interestingly, LPLI did not affect RAGE expression in SMG, and although it tended to decreased NFκB phosphorylation, this was not statistically significant. Other studies showed that laser therapy can modulate the inflammatory reaction by decreasing inflammatory infiltration and proteins as, TNF-α and INF-γ in mesenteric and periaortic lymph node cells culture [36], also it helped in the reduction of IL-6 and TNF-α levels in the serum of aging animals [37]. This is the first report to quantify its effects upon all these inflammatory markers in diabetic SMG glands.

Diabetes resulted in decreased expression of phosphorylated CREB. The CREB protein is shown to be almost exclusively located in the nucleus compartment. When activated by phosphorylation at serine 133 (Ser133), CREB interacts with its coactivator protein, CREB-binding protein (CBP), or p300 to initiate transcription of CREB- related genes [38]. Activated CREB has been proposed to inhibit NF-κB activity through competition for limiting amounts of CBP/p300 p300 [39], since this interaction is necessary for NF-κB optimal activity at some target genes [40, 41]. For the first time, we showed that the activation of CREB can be induced by LPLI in diabetic SMGs. CREB binds to a DNA target sequence named cAMP-responsive element (CRE) as a dimer, regulating diverse cellular responses, including cell proliferation, survival and differentiation [42]. Accumulation of cAMP can lead to CREB phosphorylation at Ser 133 via PKA activation [39]. Activation of PKA also mediates ERK1/2 phosphorylation [43, 44]. Intriguingly, double immunofluorescence analysis reveals the same cellular localization for LPLI-induced expression of phosphorylated CREB and ERK in diabetic salivary glands, mainly in ducts and endothelium cells. It has been reported that activated ERK can translocate into nucleus and indirectly phosphorylate CREB at Ser133 [45]. Kwon et all, showed that both CREB and ERK were necessary for fibroblasts cells proliferation *in vitro* [46]). Phosphorylated ERK binding to the DNA target sequence SRE and phosphorylated CREB binding to CRE site were required for maximal c-fos promoter activation and proliferation [46]. Although our results did not analyze cell proliferation markers, LPLI can increase cell survival by reducing apoptosis in diabetic SMG, by reduction of bax expression. Since, the cell survival is mediated by the balance between the pro-apoptotic and anti-apoptotic markers, LPLI mechanism of action against inflammation induced apoptosis might not only involve the regulation of mitochondrial apoptotic markers but also the regulation of inflammatory markers, as HMGB1 and TNF-α that trigger this process.

Diabetes increased apoptosis in SMG ducts cells. In this study, we have demonstrated that diabetes increased the cleavage of caspase-3, probably by via up-regulation of TNF-α expression, since the levels of bax, an apoptotic marker of the intrinsic pathway, was similar to the ones founded in control animals. Increased expression of apoptotic cell death proteins was also reported in our previous study [5]. The pro-apoptotic markers, TNF-α and bax, were strongly repressed by LPLI in diabetic tissues, suggesting that the laser protective effects might be mediated by the regulation of mitochondrial apoptotic proteins. In accordance to our results, LPLI also suppressed TNF-α secretion in bone marrow stem cells under inflammatory conditions, and promote proliferation and osteogenesis *in vitro* [47]. LPLI also prevented apoptosis induced by TNF-α in endothelial cells, down-regulating caspase 3, 7, 8 and 9 expression and enhancing ERK phosphorylation [48].

This laser protective effect could be related to the ERK activation observed in the present study. Yan et al. showed that low-level laser irradiation induction of CREB phosphorylation is dependent on ERK, and ERK/CREB pathway were important for laser beneficial effects in neuronal survival [34].

ERK was also reported to inhibit caspase-9 via phosphorylation of T125 which inhibits activation of cleaved-caspase-3 and apoptosis, it is also involved in the modulation of the activity of many proteins involved in apoptosis [49].

Yun *et al*. also reported that laser therapy could not only up-regulate CREB gene expression to normal levels in rats hippocampus after injury, but also increase bcl-2, an anti-apoptotic protein, and decrease bax expression, preventing neurodegeneration [33]. LPLI also protected macrophages and muscle precursors cells from apoptosis after injury in gilts [50]. Its biomodulation effects were also reported in an animal experimental model of osteoarthritis treated with laser, where it significantly reduced caspase-3 expression [51]. In our study, a single time LPLI could not significantly reduce cleaved-caspase-3 expression in diabetic rats SMG, but did reduce apoptosis in this tissue. We hypothesized that LPLI might have an effect in non-caspase apoptotic pathways regulation.

**Fig 8:**
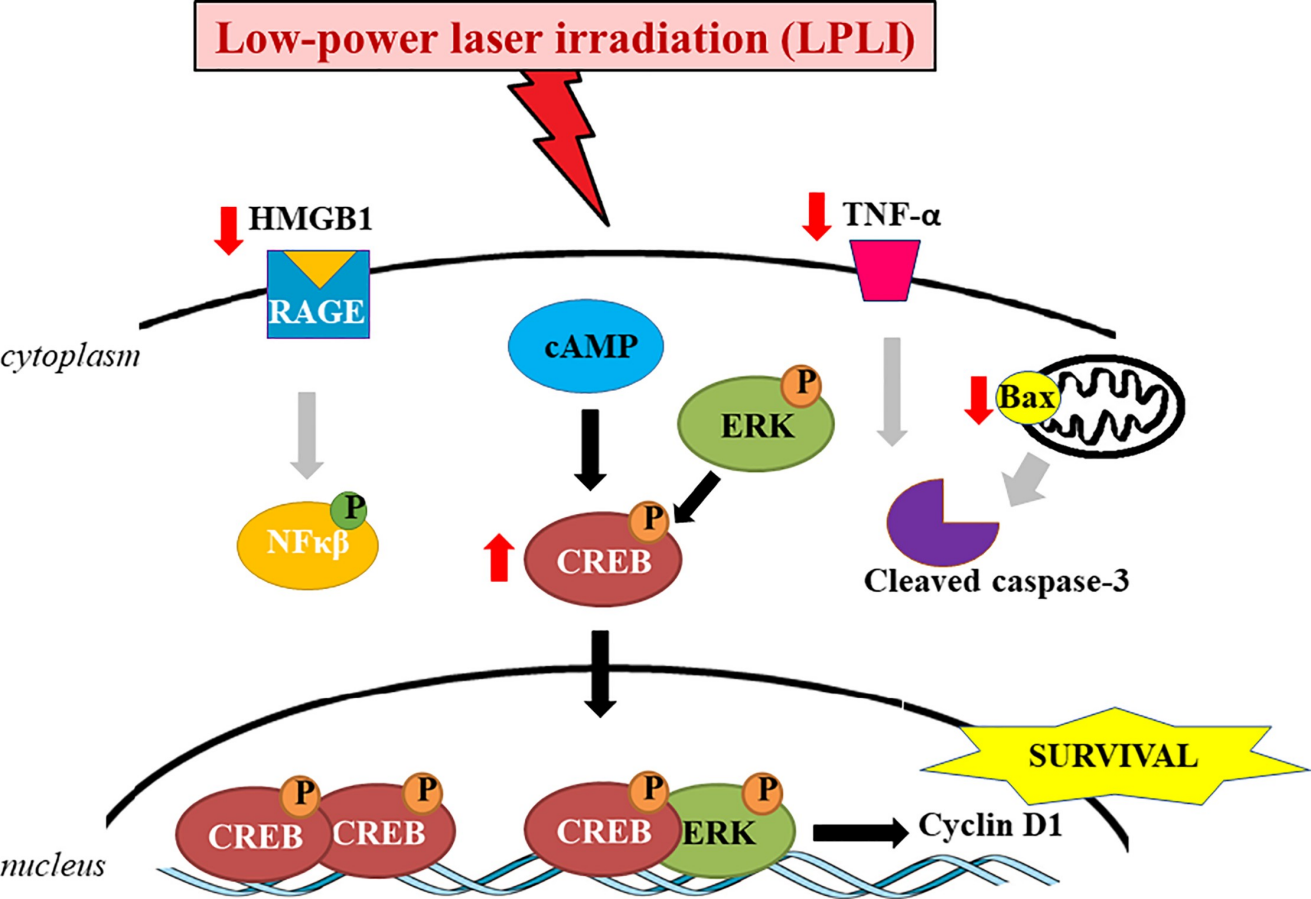
LPLI effects upon diabetic SMGs. Diabetes significantly increased the inflammatory markers, HMGB1 and its ligand RAGE, and promoted apoptosis by up-regulation of TNF-α expression and caspase-3 cleavage. Interestingly, LPLI strongly decreased HMGB1 and TNF-α accumulation in the tissue, also promoted the activation of the transcription factor CREB, by its phosphorylation on Ser 133, and reduced the expression of bax, therefore, modulating apoptosis and favoring the cell survival in diabetic SMG.

In agreement with our previous findings [5, 6], LPLI significantly reduced the hyperglycemia in diabetic rats. Our results suggest a mechanism of action of LPLI (Fig 8), where it could efficiently down-regulate diabetes-induced HMGB1 and TNF-α expression. Reducing the expression of bax as well, and preventing apoptosis in diabetic SMG. At the same time, LPLI up-regulated the activation of CREB pathway, partly through ERK, favoring cell survival. These findings indicate a promising therapeutic horizon for LPLI and deserves further studies to enlighten LPLI mechanisms of action and its effects in a long-term experiment.

## Conclusion

LPLI can reduce inflammation and apoptosis in diabetic SMG, possibly by activating cAMP/CREB signaling pathway, partially mediated by ERK.

## Supporting information

S1 Fig Rat experimental model. A. Procedures schedule. B. LPLI points distribution. C0- control. D0- diabetic rats. D20- diabetic rats treated with LPLI.
(TIF)

S2 Fig Co-localization of phosphorylated CREB and phosphorylated ERK assessed by double immunofluorescence analysis in SMG. In I, arrows points to double positive-stained striated and excretory ducts cells (n = 1/group). Scale bar, 50μM. ED, excretory duct; SD, striated duct.
(TIF)

S1 Table LPLI decreased diabetic animals blood glucose levels. Body weight on the 1^st^ experimental day (initial) and in the 30^th^ day (final). Blood glucose levels were measured in the 3^rd^ experimental day (initial) and in the day of the sacrifice (final), after 12h-fasting (n = 8/group). Data are presented as mean ± SD; * P < 0.05 vs control group (C0); # P < 0.05 vs respective diabetic group (D0); ⱡ P < 0.05 vs initial time in the same group.
(DOCX)

S1 Raw file (ZIP)
