# Supplementary figures and images for: Early effect of laser irradiation in signaling pathways of diabetic rat submandibular salivary glands
Source: PLoS One. 2020 Aug 4;15(8):e0236727. doi: 10.1371/journal.pone.0236727 (PMC7402516; doi:10.1371/journal.pone.0236727)

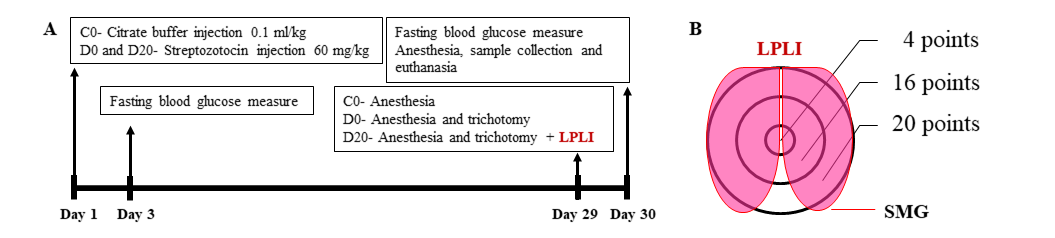

Supplement: S1 Fig — A. Procedures schedule. B. LPLI points distribution. C0- control. D0- diabetic rats. D20- diabetic rats treated with LPLI. (TIF) [file pone.0236727.s001.tif]

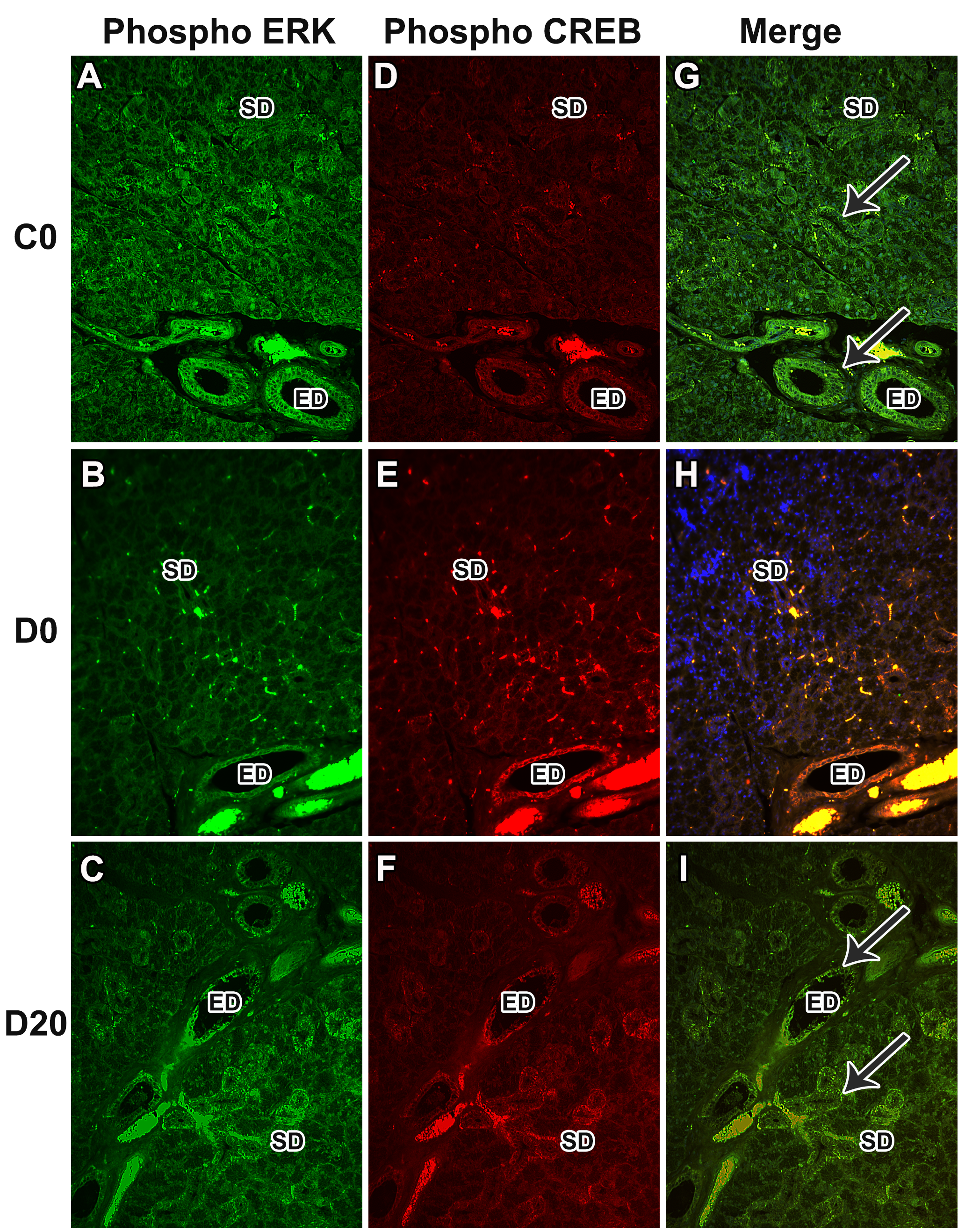

Supplement: S2 Fig — In I, arrows points to double positive-stained striated and excretory ducts cells (n = 1/group). Scale bar, 50μM. ED, excretory duct; SD, striated duct. (TIF) [file pone.0236727.s002.tif]

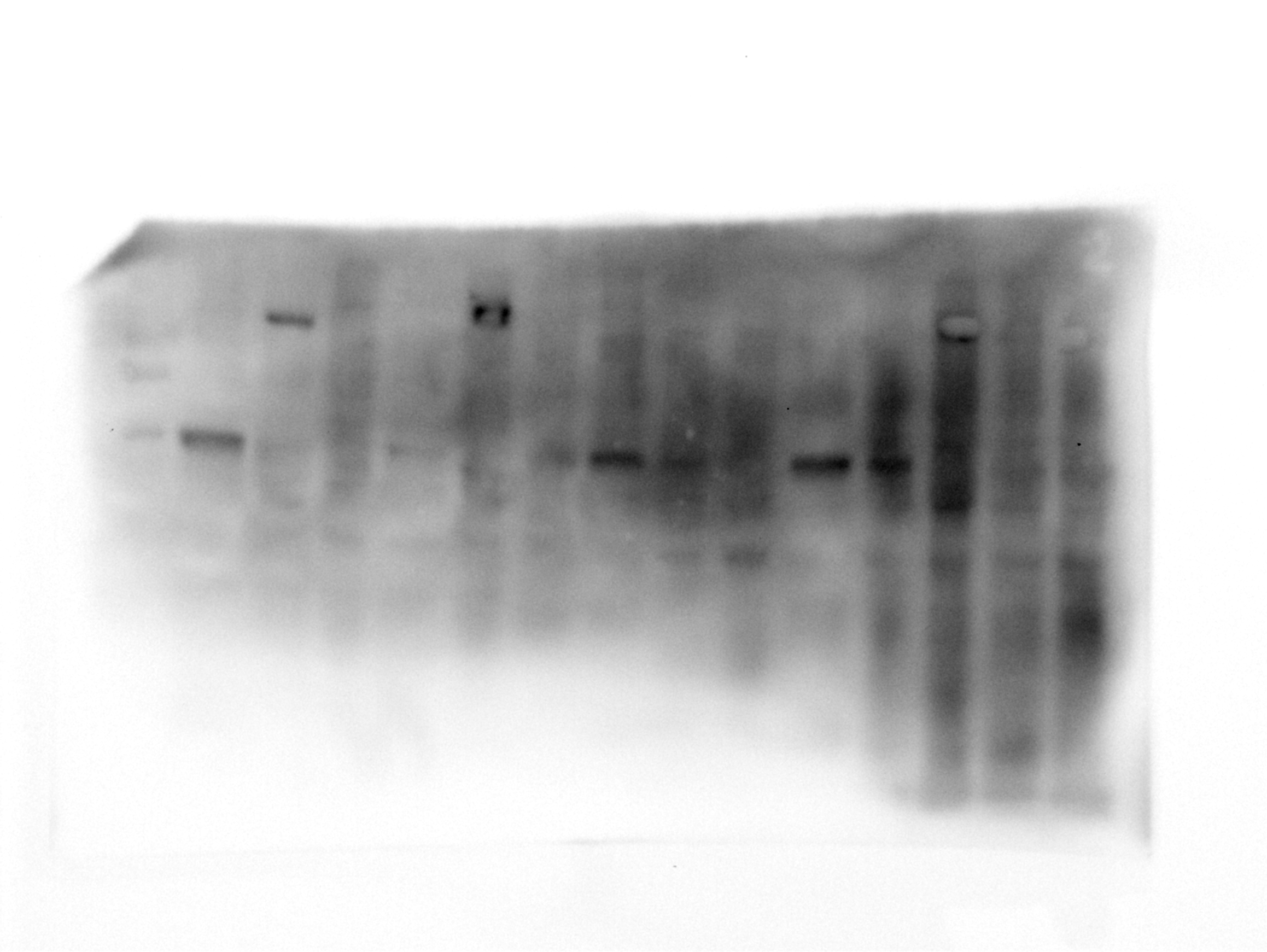

Supplement: S1 Raw file — (ZIP) [file pone.0236727.s004.zip › 130716-gel2-NFkB-F-3-WB.tif]

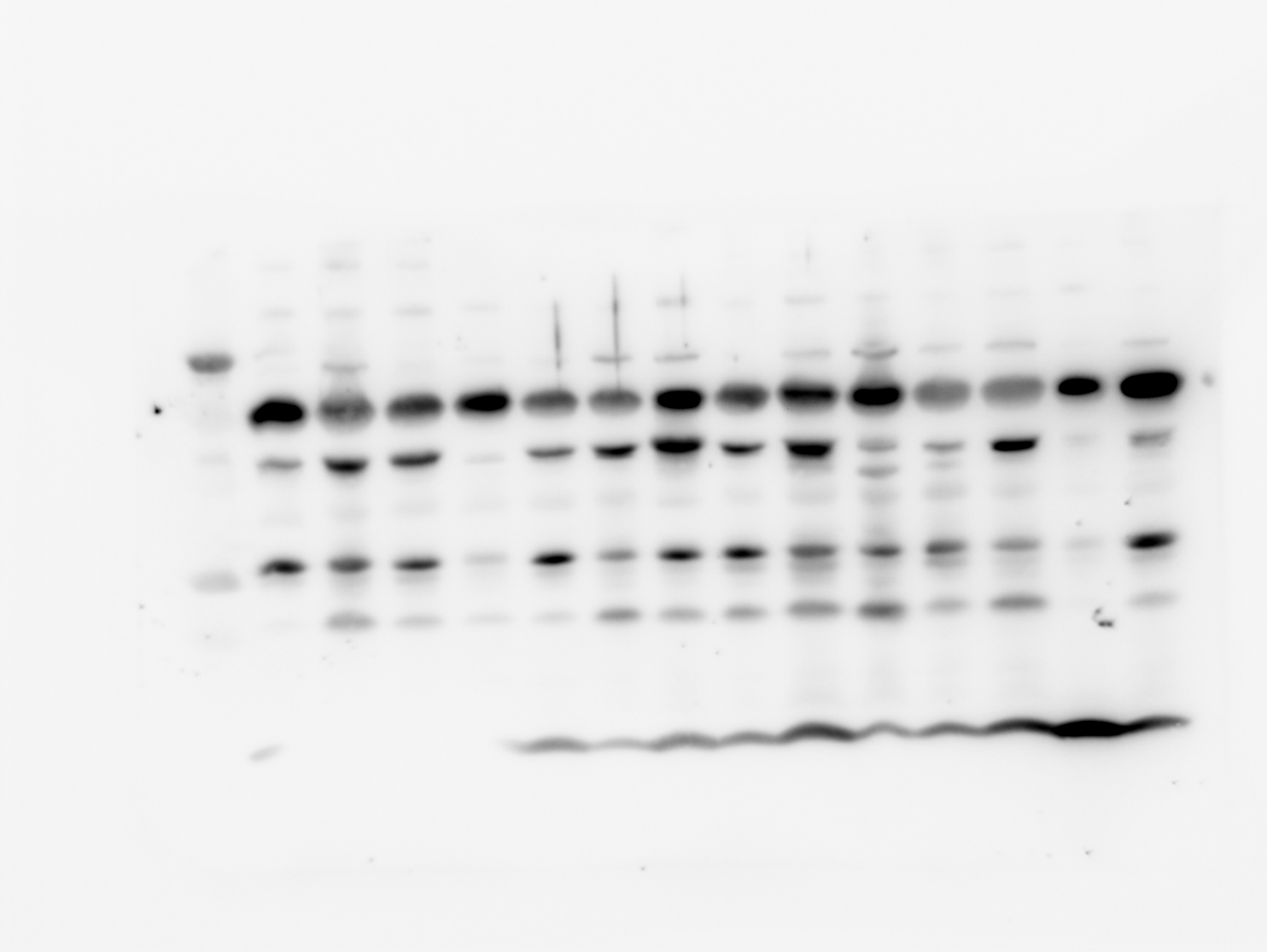

Supplement: S1 Raw file — (ZIP) [file pone.0236727.s004.zip › 130716-gel3-RAGE-F-WB.tif]

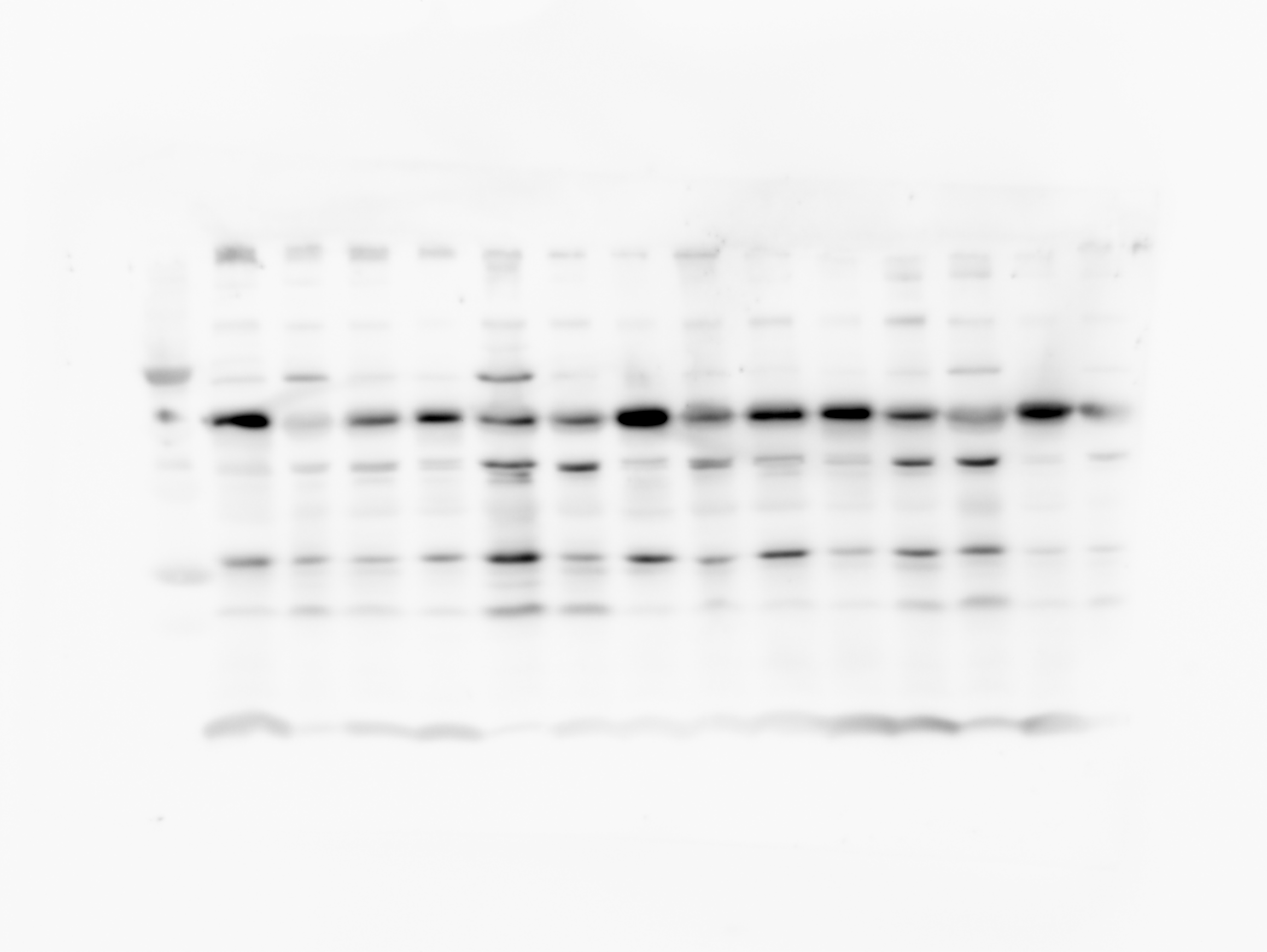

Supplement: S1 Raw file — (ZIP) [file pone.0236727.s004.zip › 130716-gel4-RAGE-F-2leitura-WB.tif]

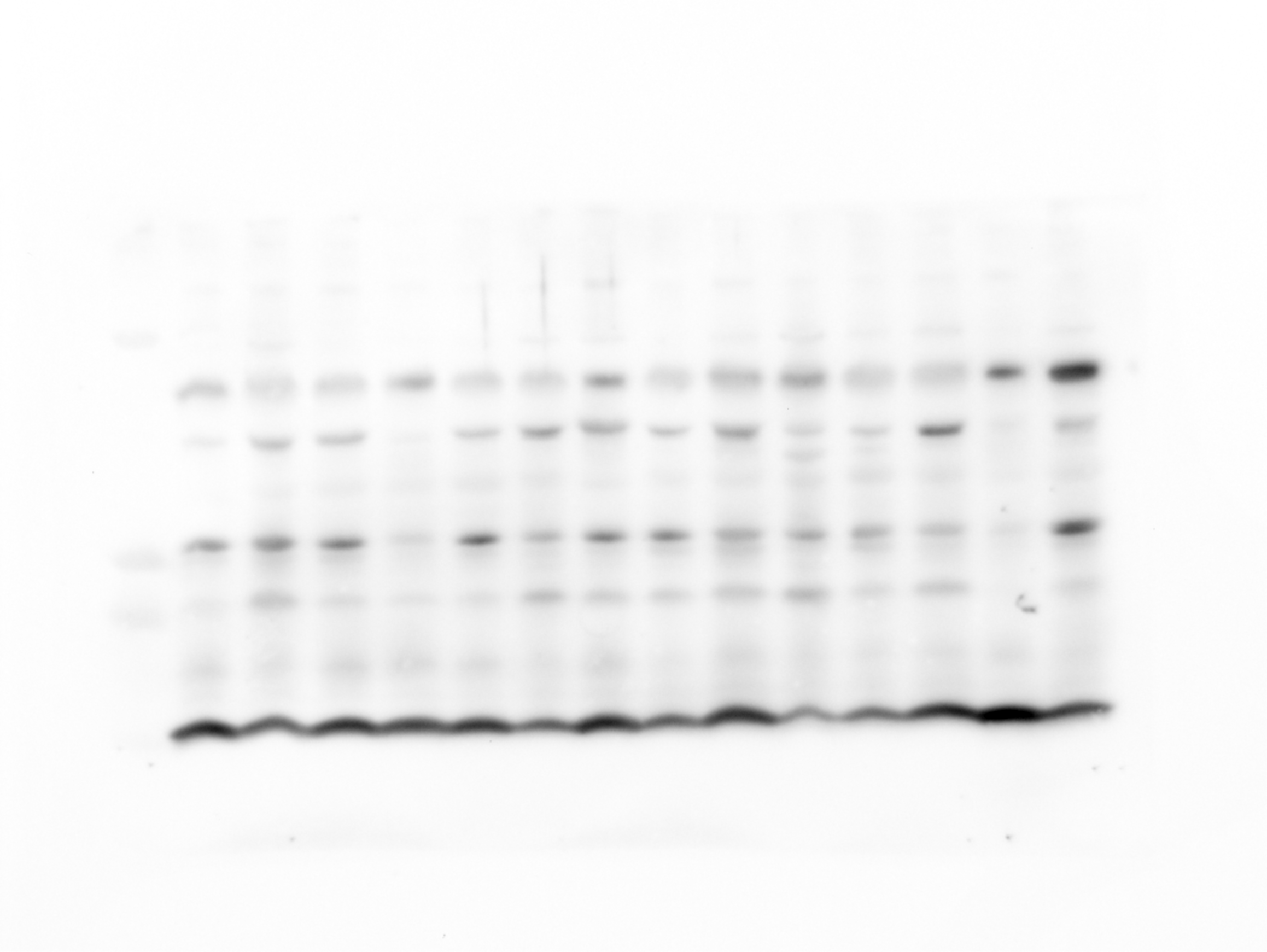

Supplement: S1 Raw file — (ZIP) [file pone.0236727.s004.zip › 140716-gel3.2-caspase-3-2-original-1.tif]

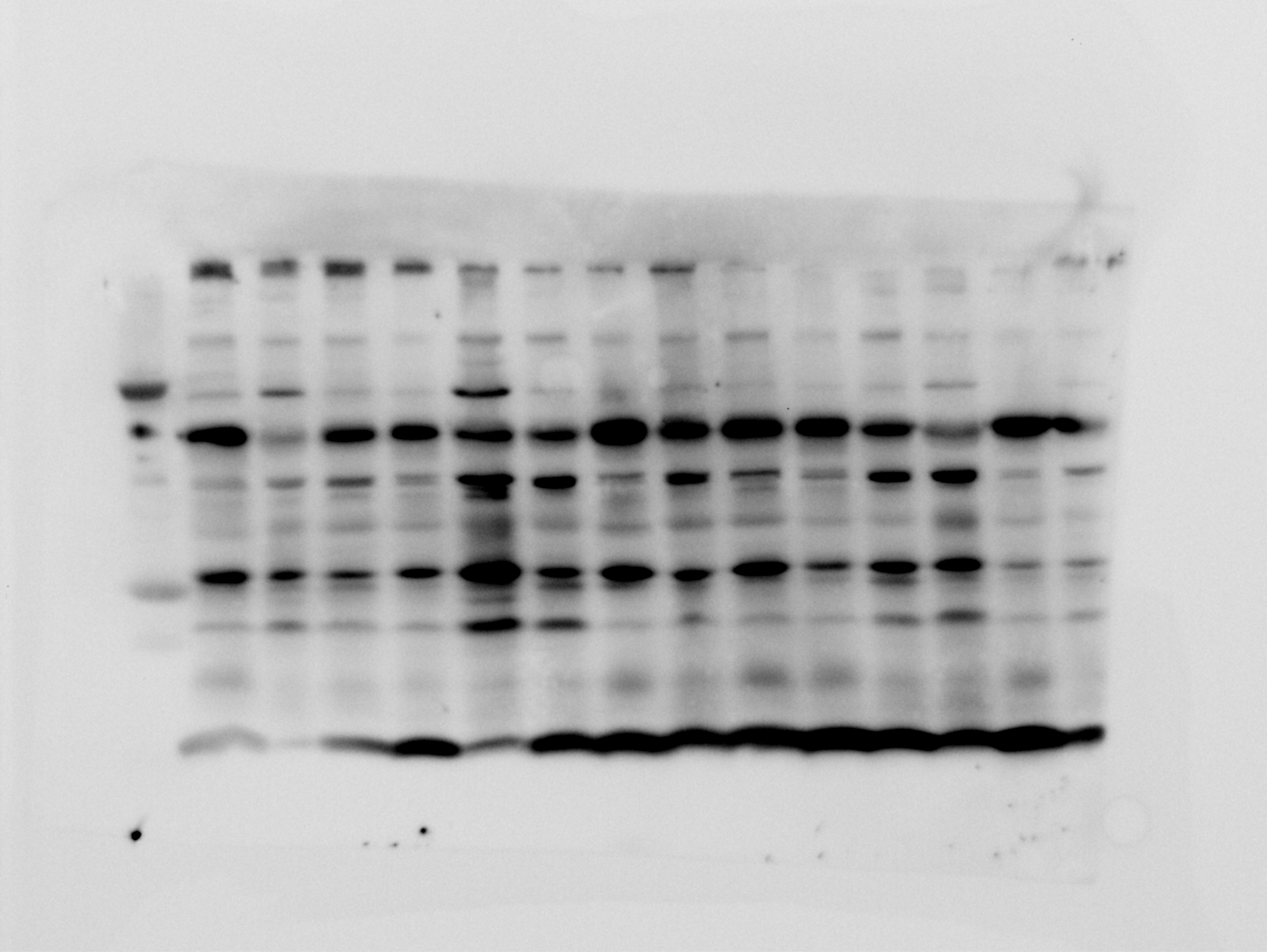

Supplement: S1 Raw file — (ZIP) [file pone.0236727.s004.zip › 140716-gel4.2-caspase-3-2WB.tif]

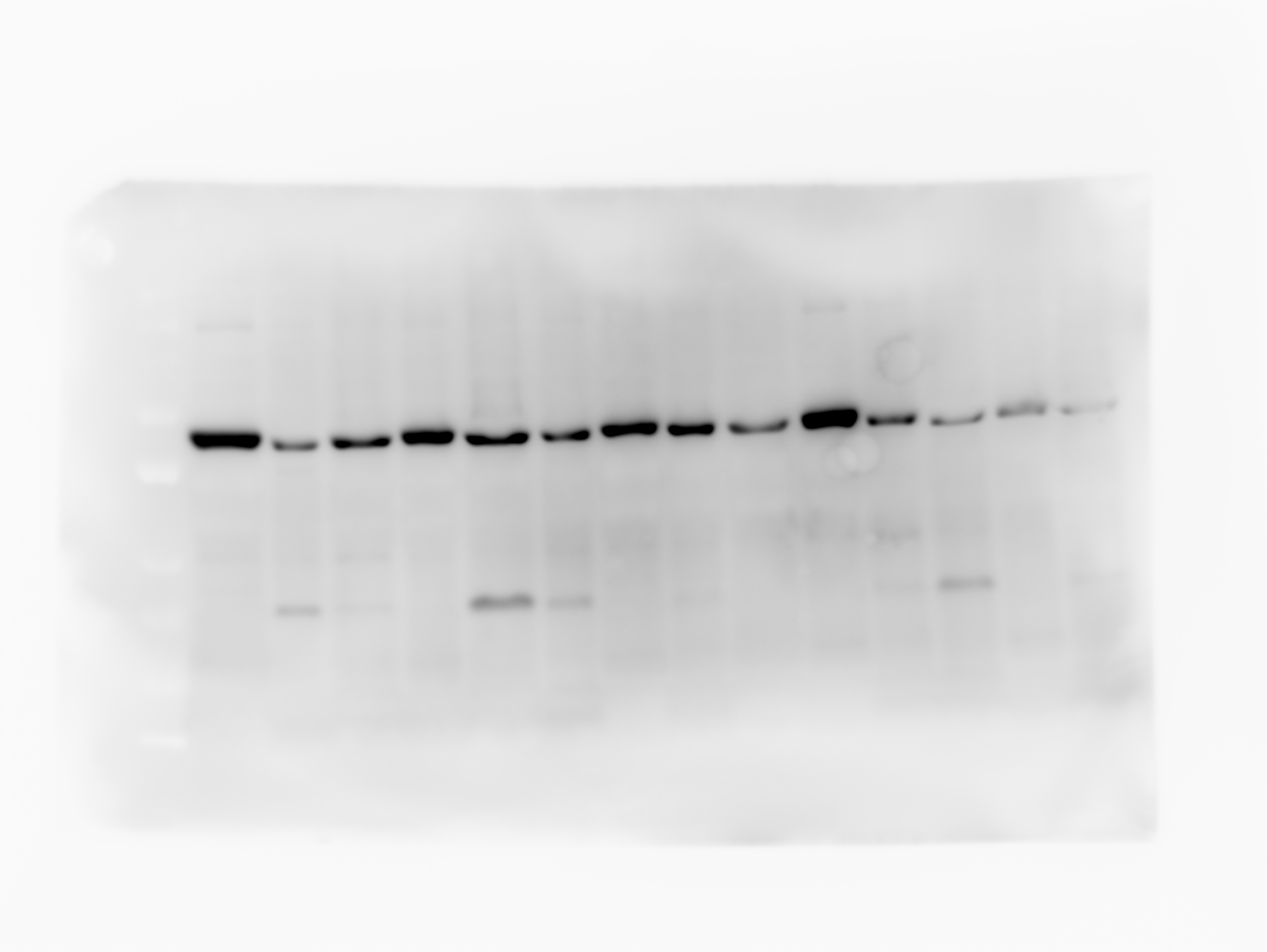

Supplement: S1 Raw file — (ZIP) [file pone.0236727.s004.zip › 140716-gel5.2-bax-3-original.tif]

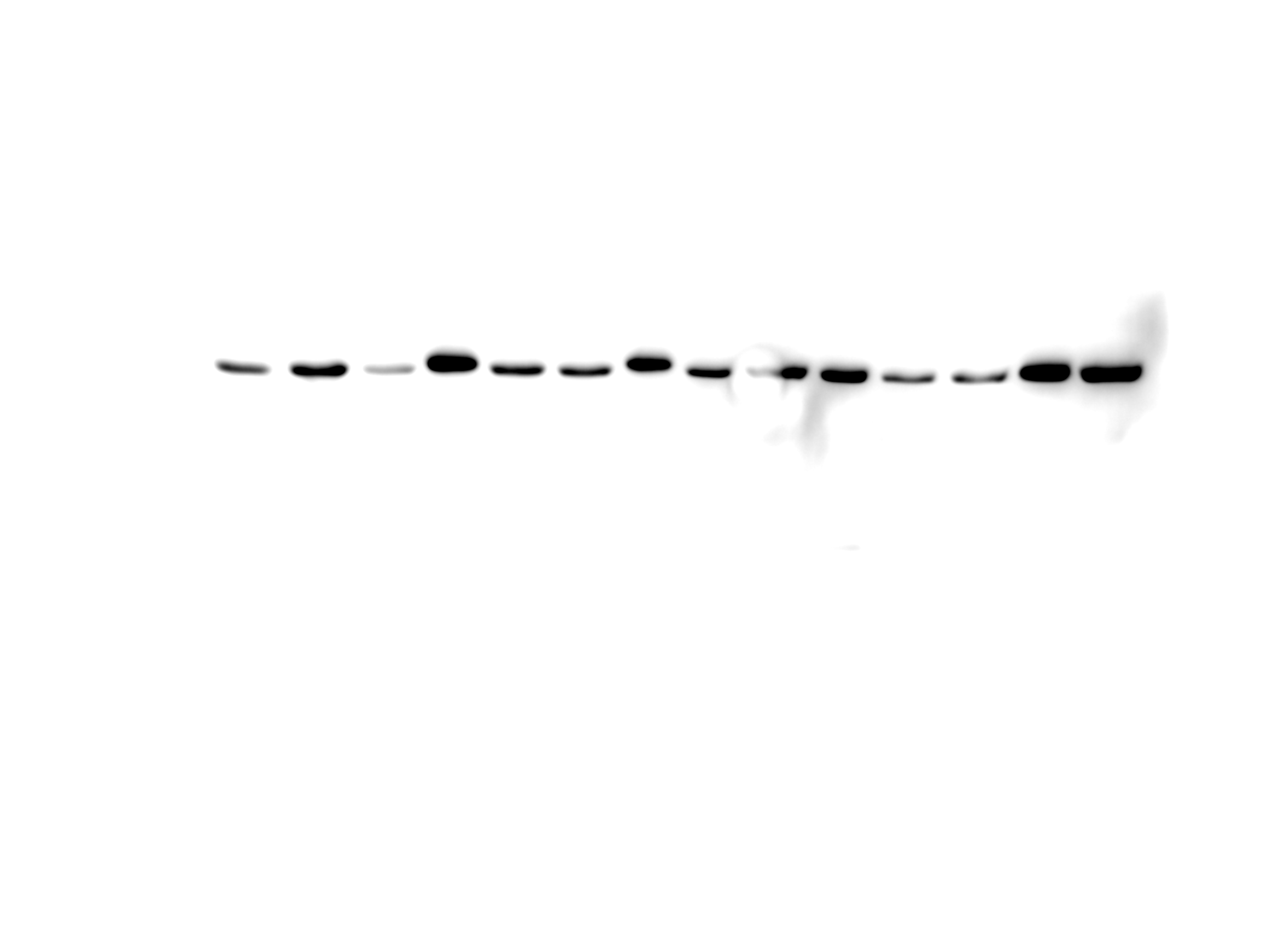

Supplement: S1 Raw file — (ZIP) [file pone.0236727.s004.zip › 140716-gel6.2-bax--1-WB.tif]

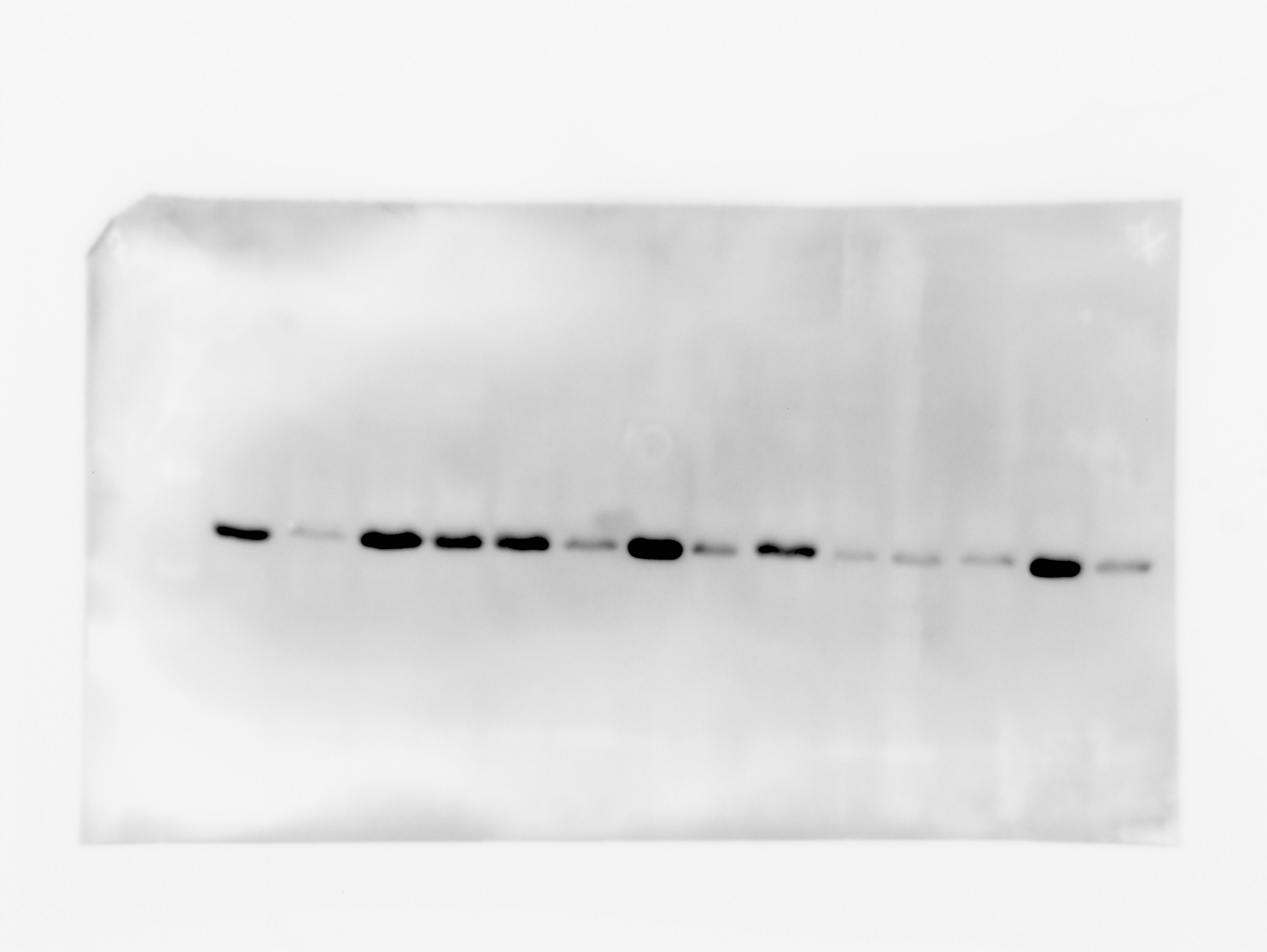

Supplement: S1 Raw file — (ZIP) [file pone.0236727.s004.zip › 140716-gel7.2-phosphoCREB--2-WB-foto.tif]

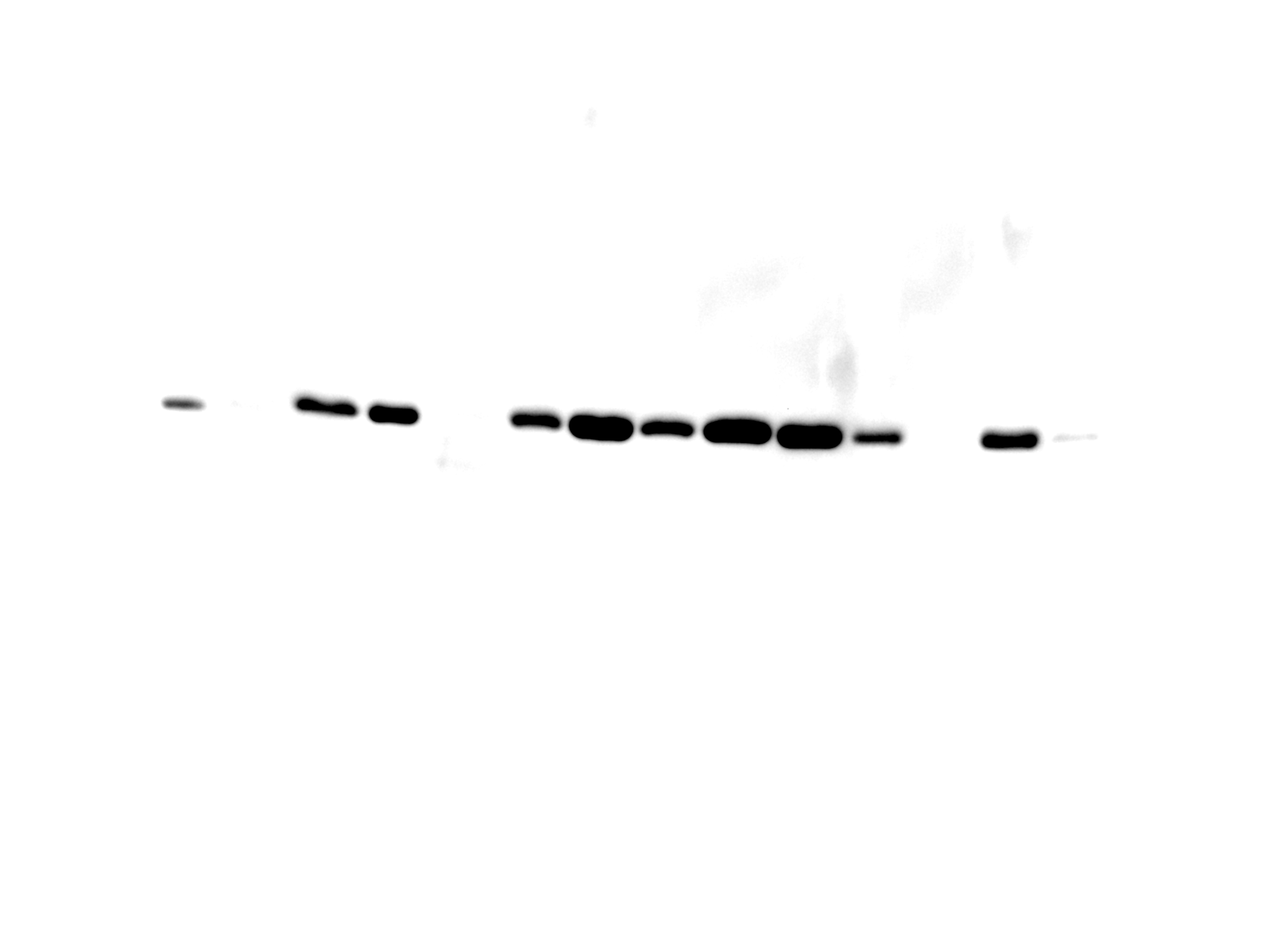

Supplement: S1 Raw file — (ZIP) [file pone.0236727.s004.zip › 140716-gel8.2-phosphoCREB-3-WB.tif]

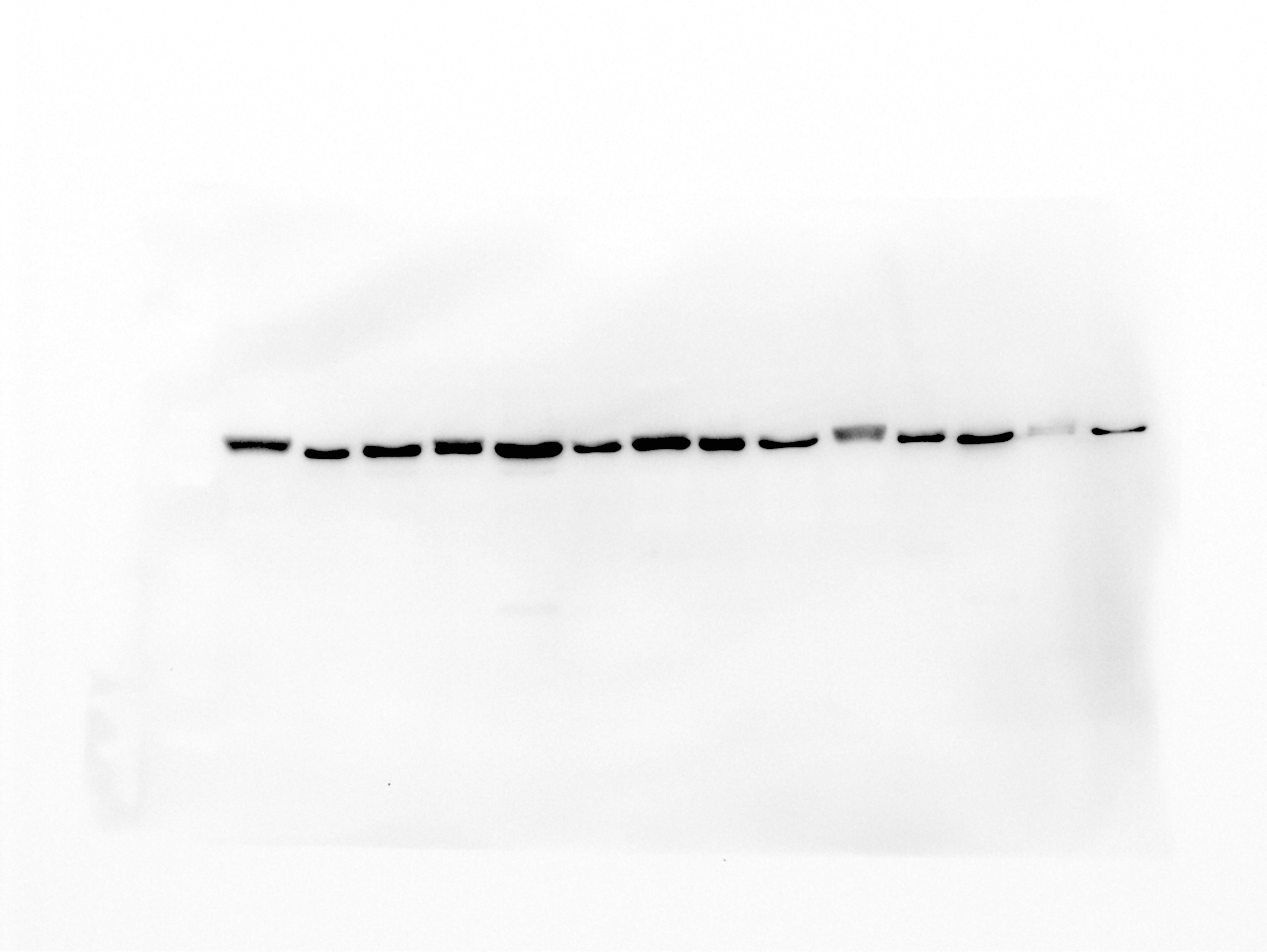

Supplement: S1 Raw file — (ZIP) [file pone.0236727.s004.zip › 150716-gel5.2-beta-actina-2-WB.tif]

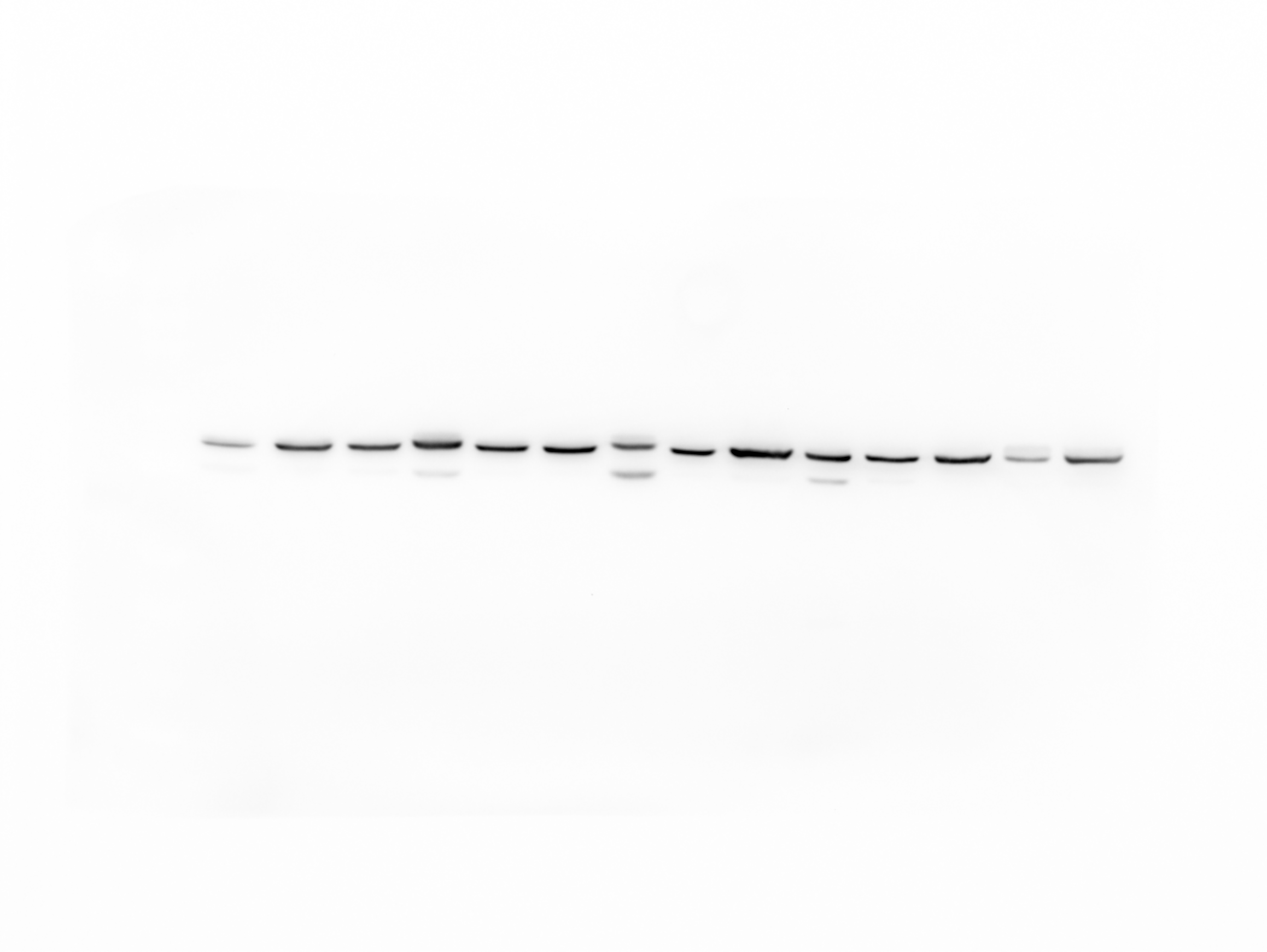

Supplement: S1 Raw file — (ZIP) [file pone.0236727.s004.zip › 150716-gel62-beta-actina-2-original.tif]

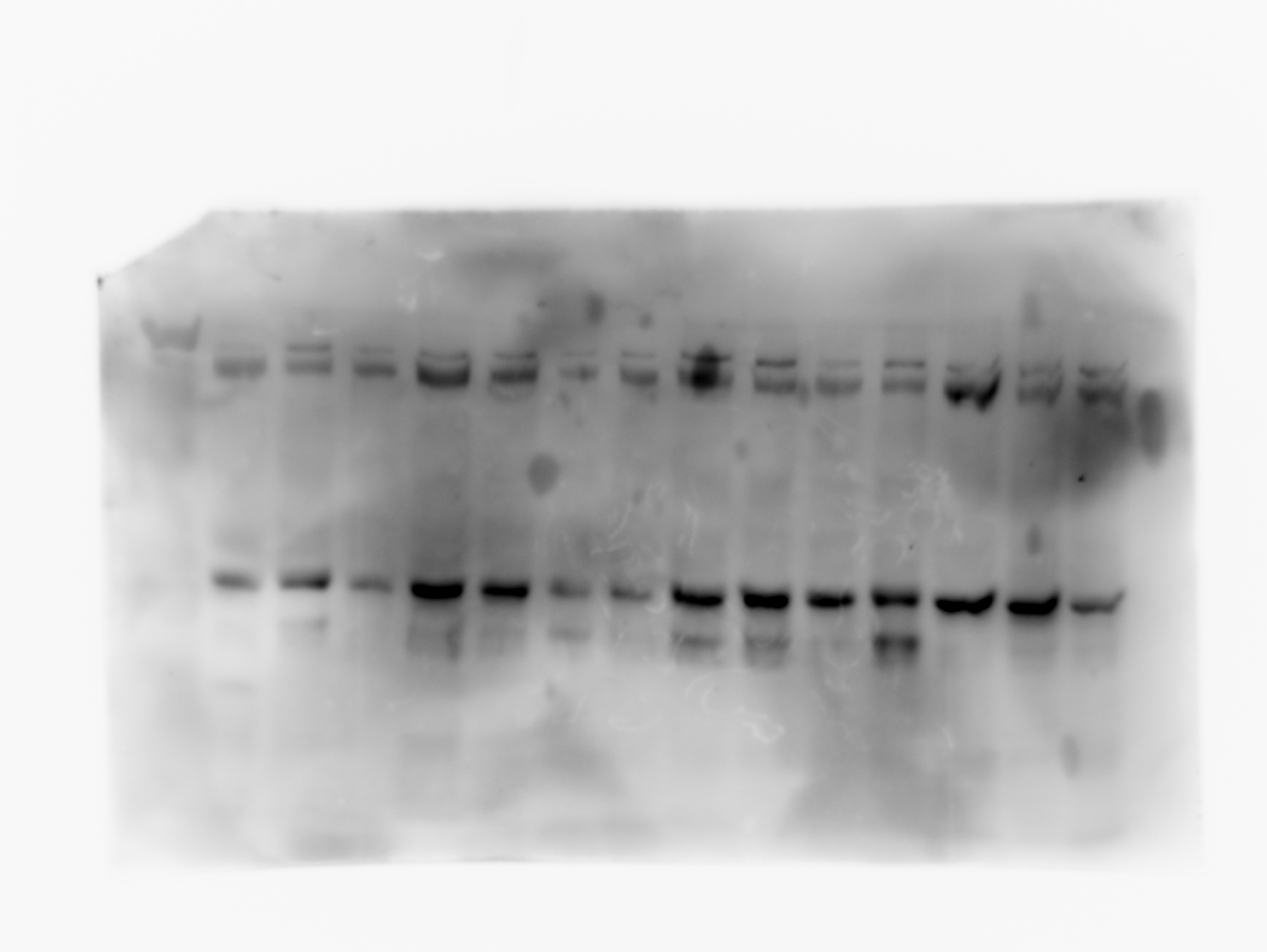

Supplement: S1 Raw file — (ZIP) [file pone.0236727.s004.zip › 300616-gel1-NFKB-reprobing-4leitura-C-WB-1.tif]

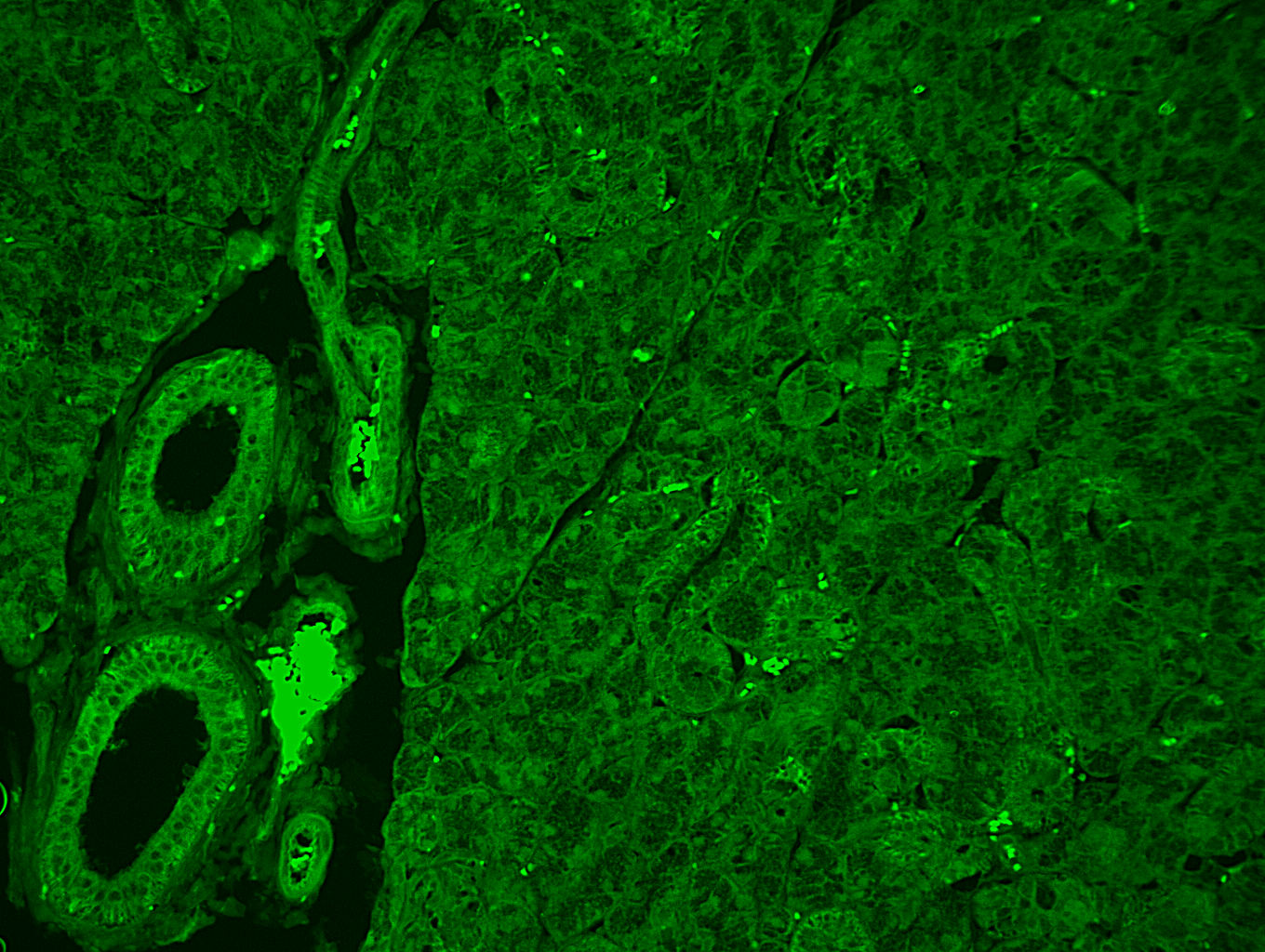

Supplement: S1 Raw file — (ZIP) [file pone.0236727.s004.zip › C30-0J-pERK.tif]

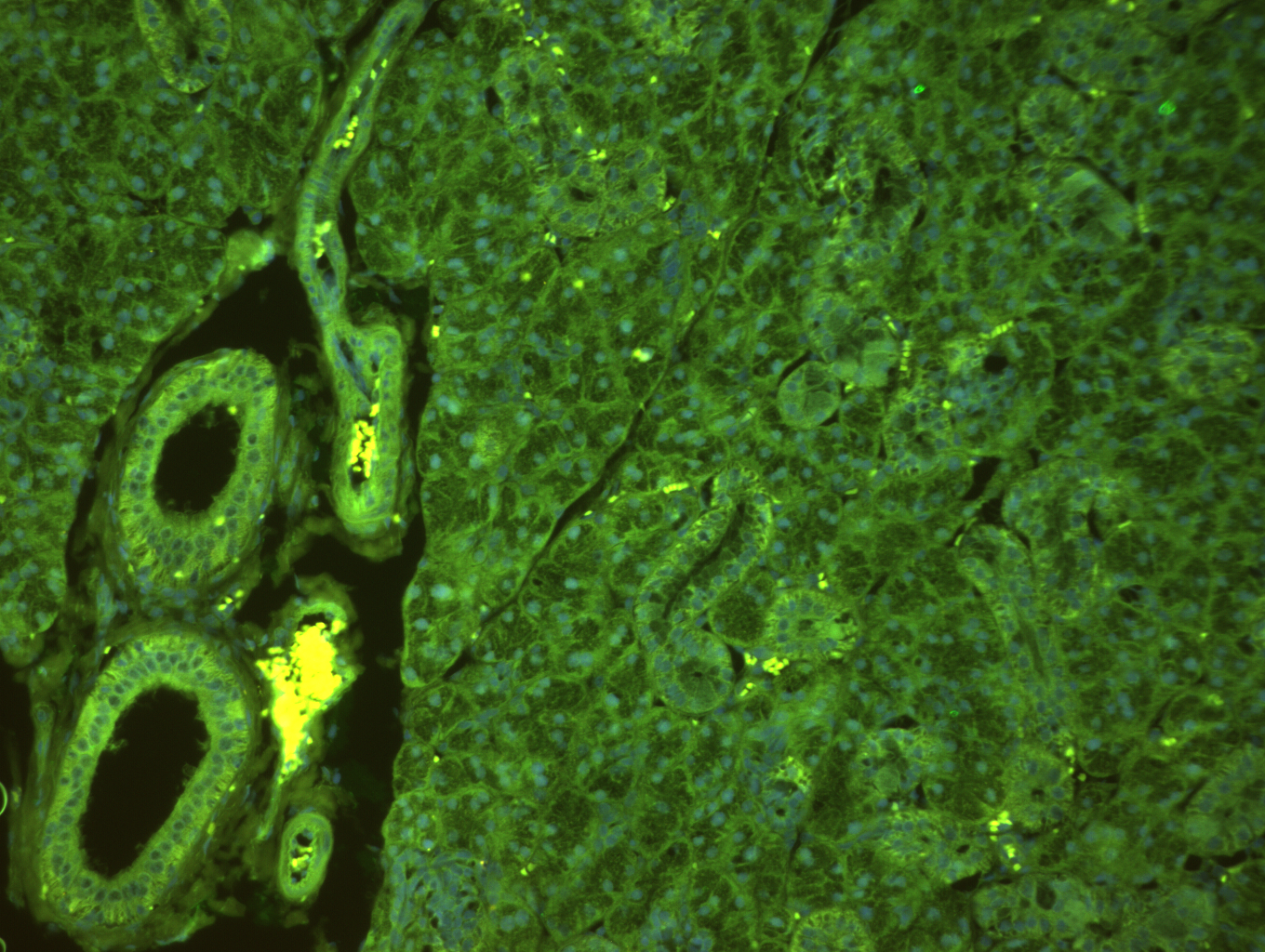

Supplement: S1 Raw file — (ZIP) [file pone.0236727.s004.zip › C30-0J-pERKCREB.TIF]

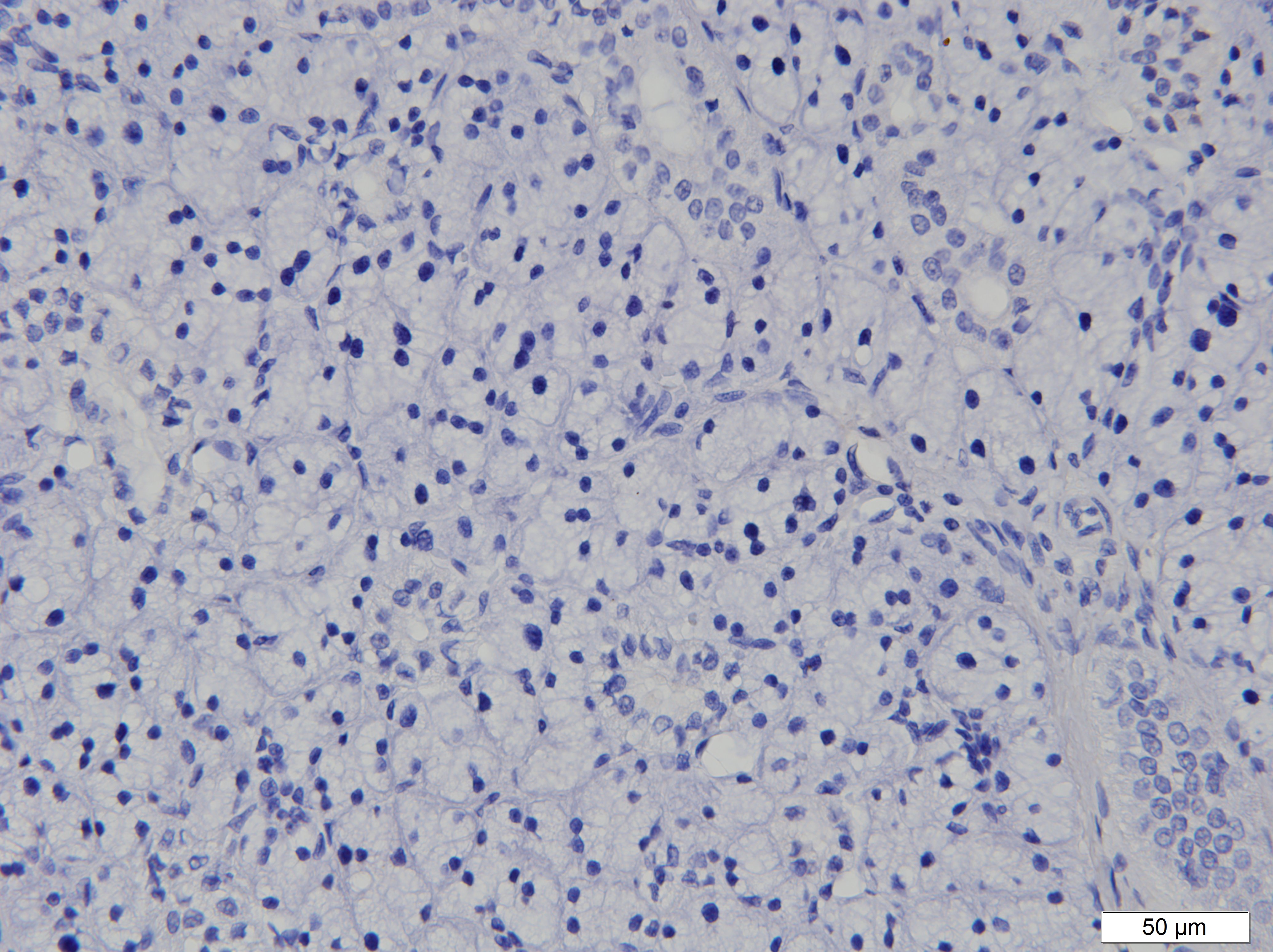

Supplement: S1 Raw file — (ZIP) [file pone.0236727.s004.zip › C30-0J-SM cAMPX40.jpg]

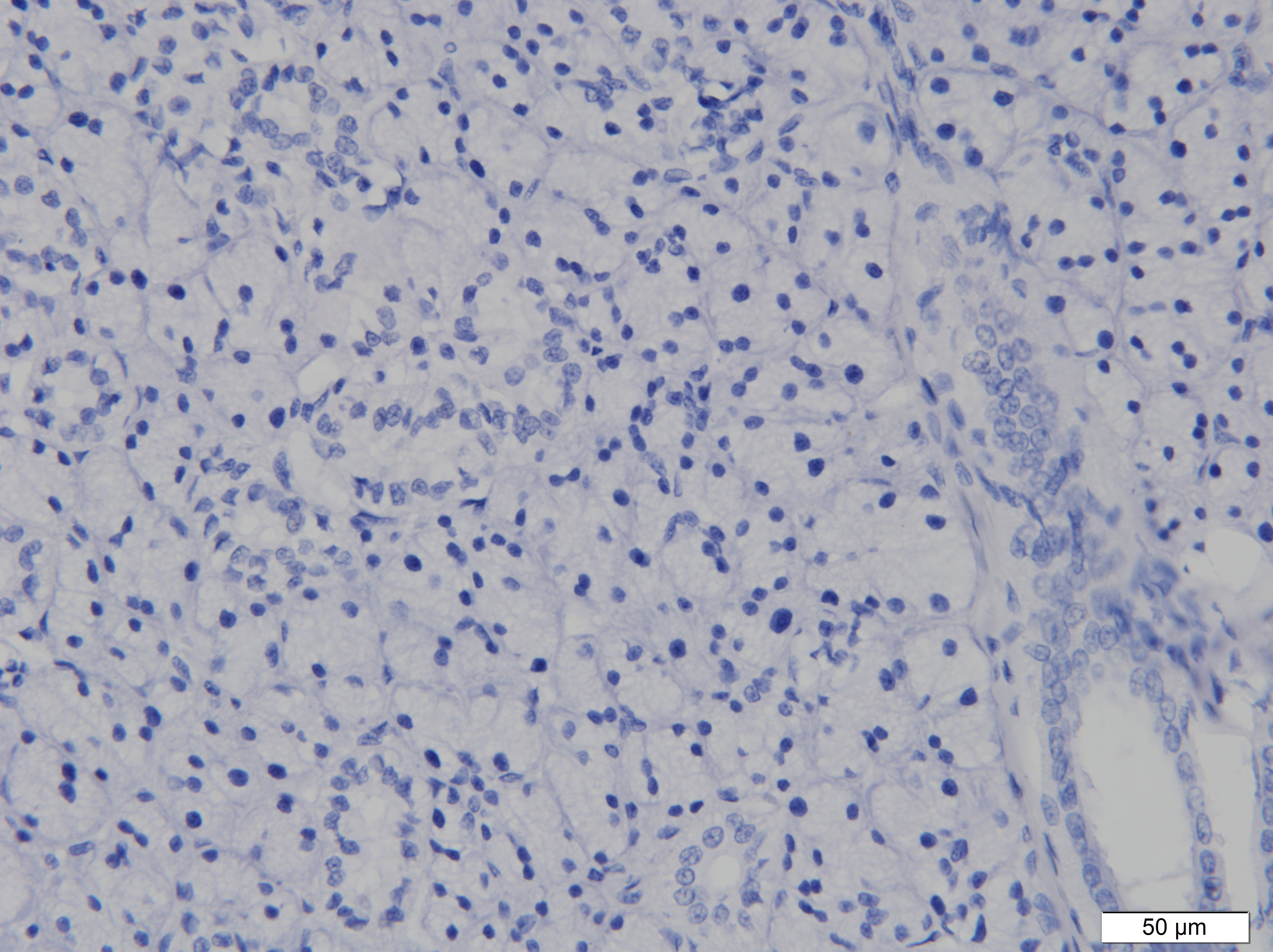

Supplement: S1 Raw file — (ZIP) [file pone.0236727.s004.zip › C30-0J-SM phospho ERKX40-10.jpg]

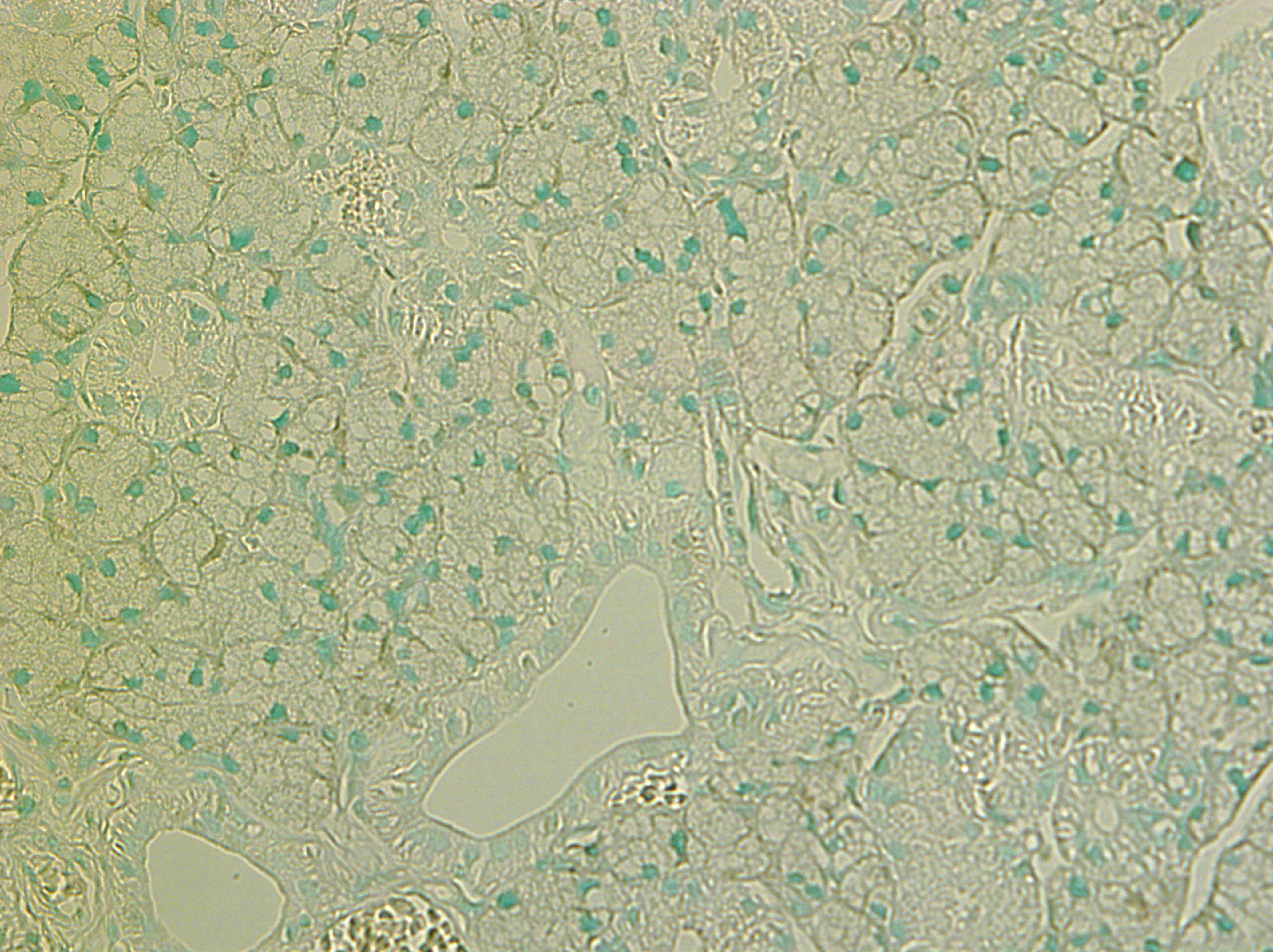

Supplement: S1 Raw file — (ZIP) [file pone.0236727.s004.zip › control 5 SMG0J TUNEL x40s.tif]

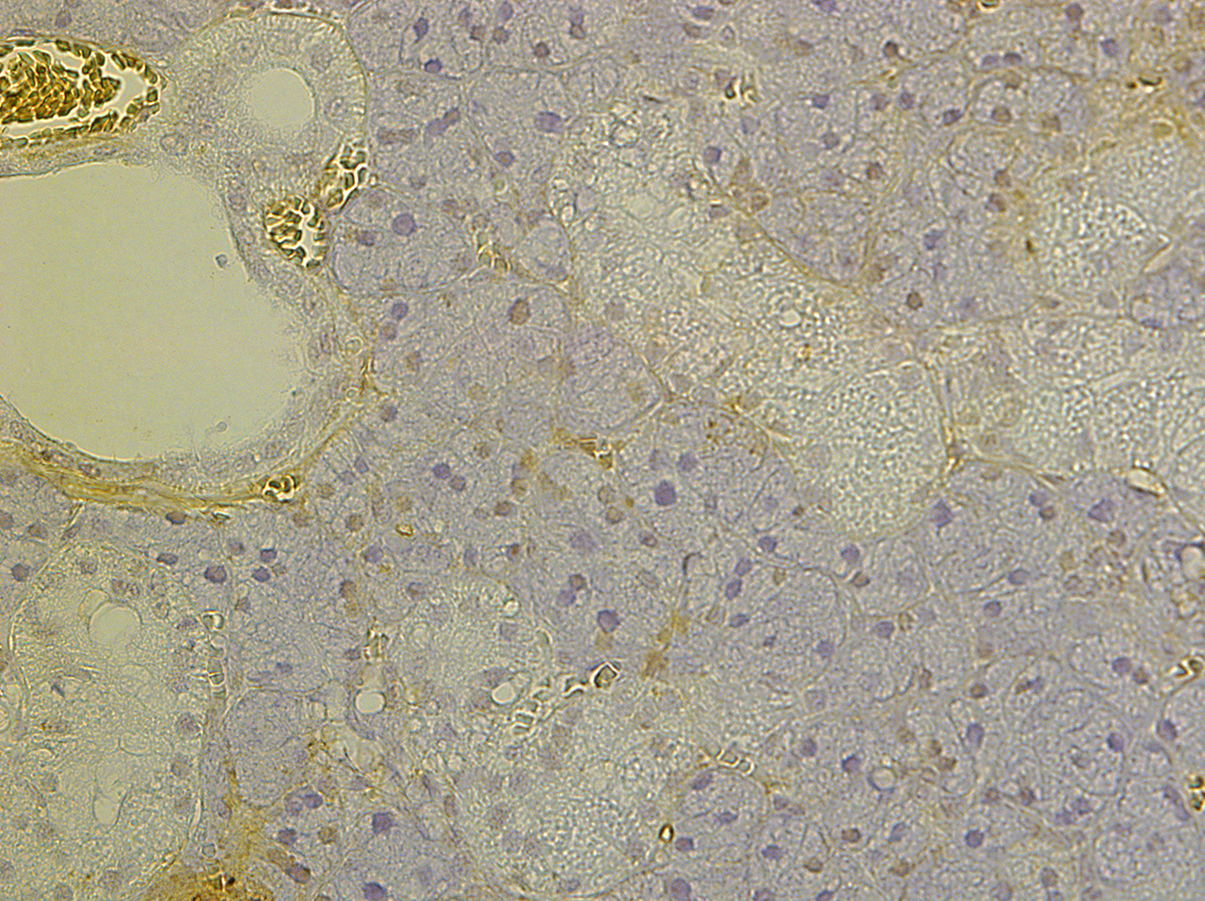

Supplement: S1 Raw file — (ZIP) [file pone.0236727.s004.zip › control3 SMG0J phosphoCREB x40s.tif]

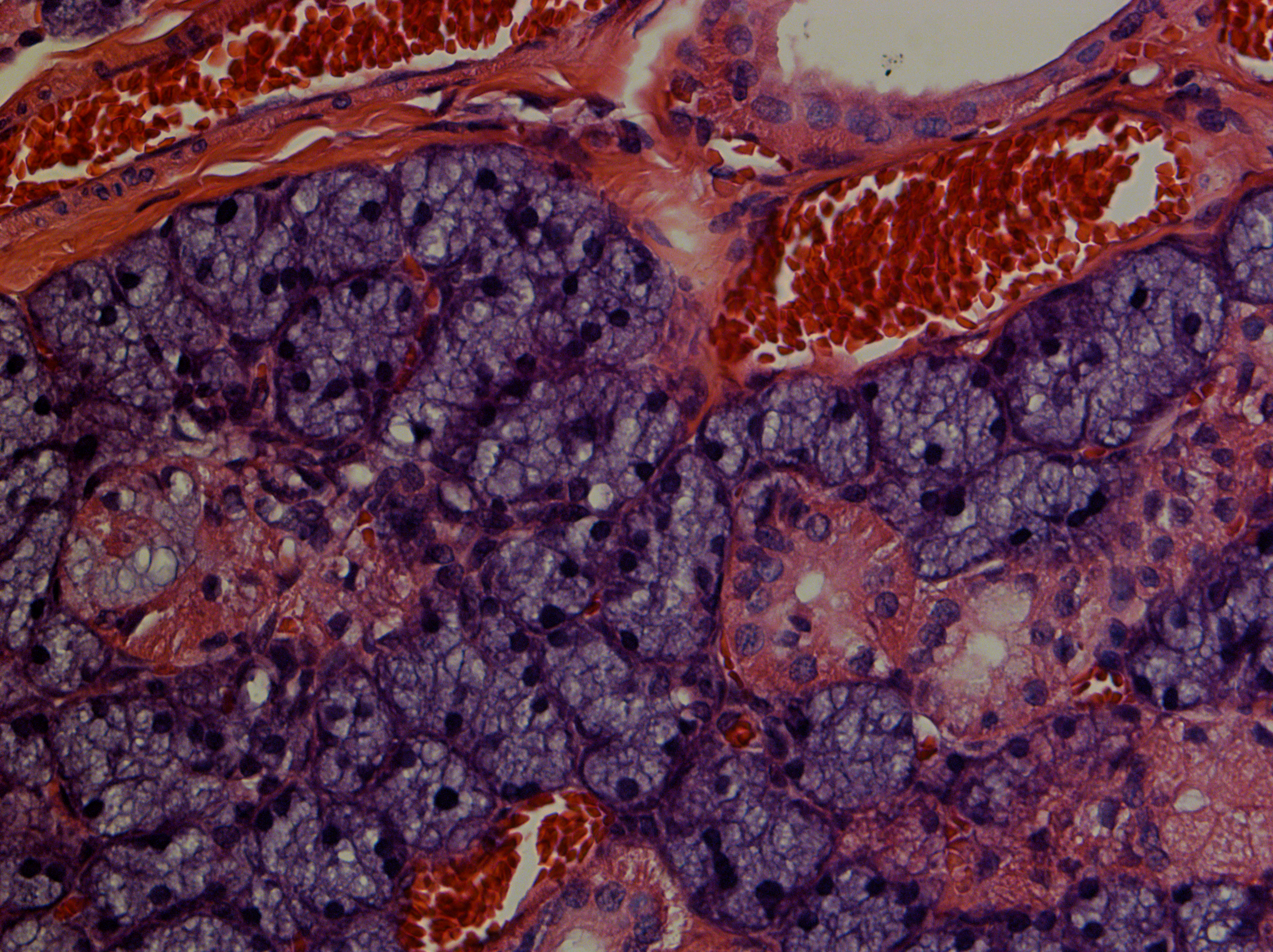

Supplement: S1 Raw file — (ZIP) [file pone.0236727.s004.zip › control4 SMG0J HEX40s.tif]

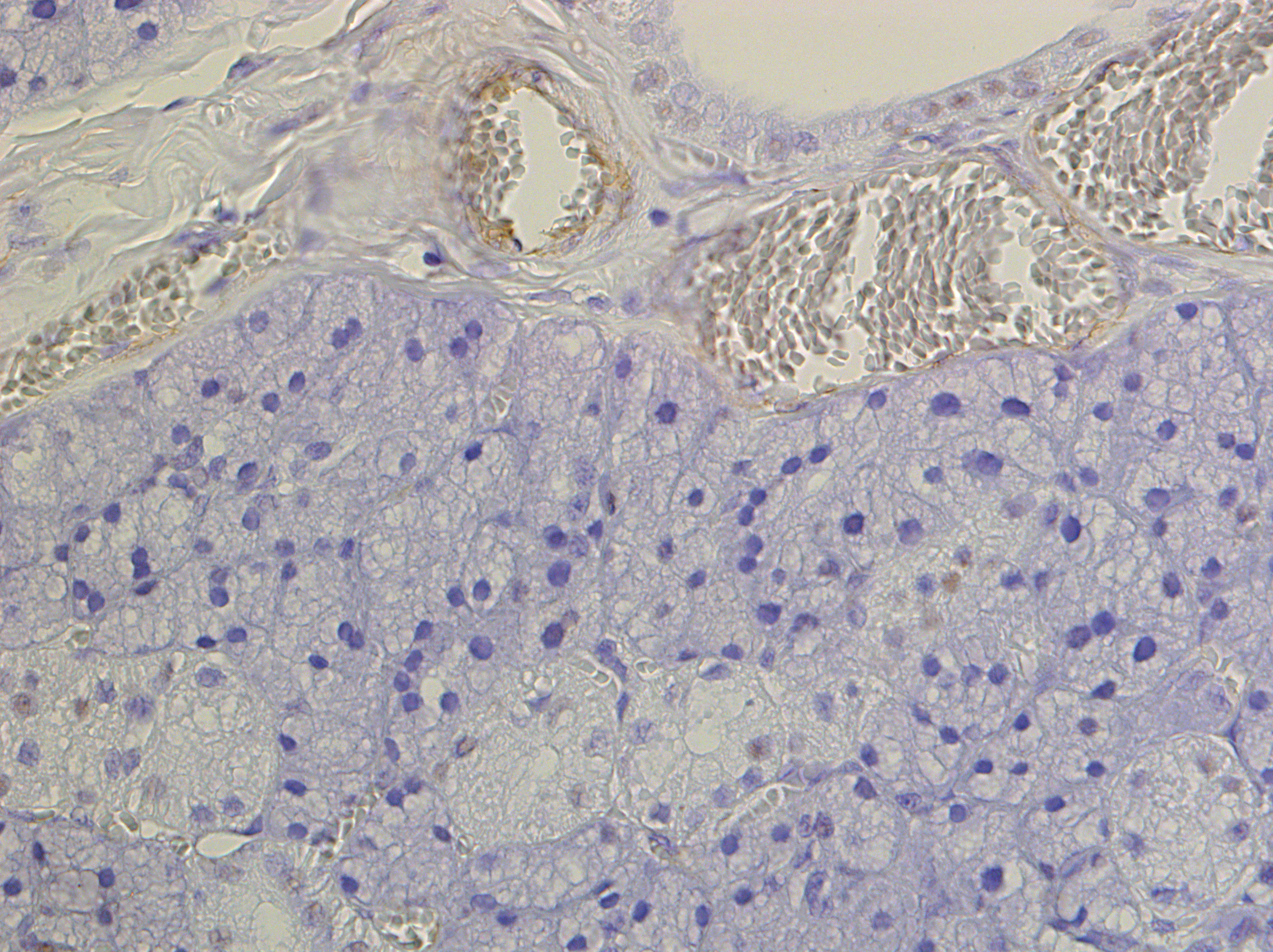

Supplement: S1 Raw file — (ZIP) [file pone.0236727.s004.zip › control4 SMG0J HMGB1X40s.tif]

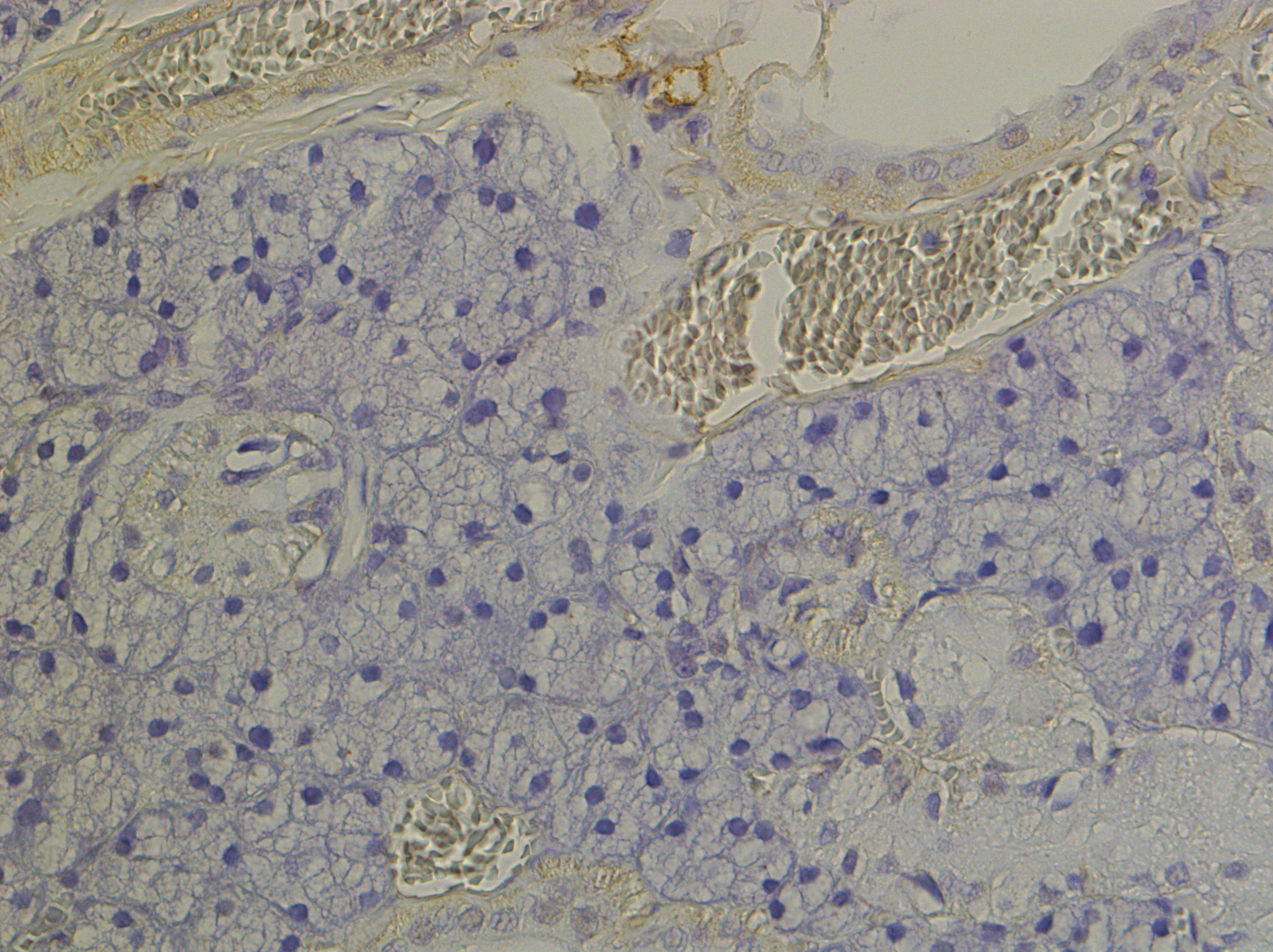

Supplement: S1 Raw file — (ZIP) [file pone.0236727.s004.zip › control4 SMG0J phosphoNFkBX40s.tif]

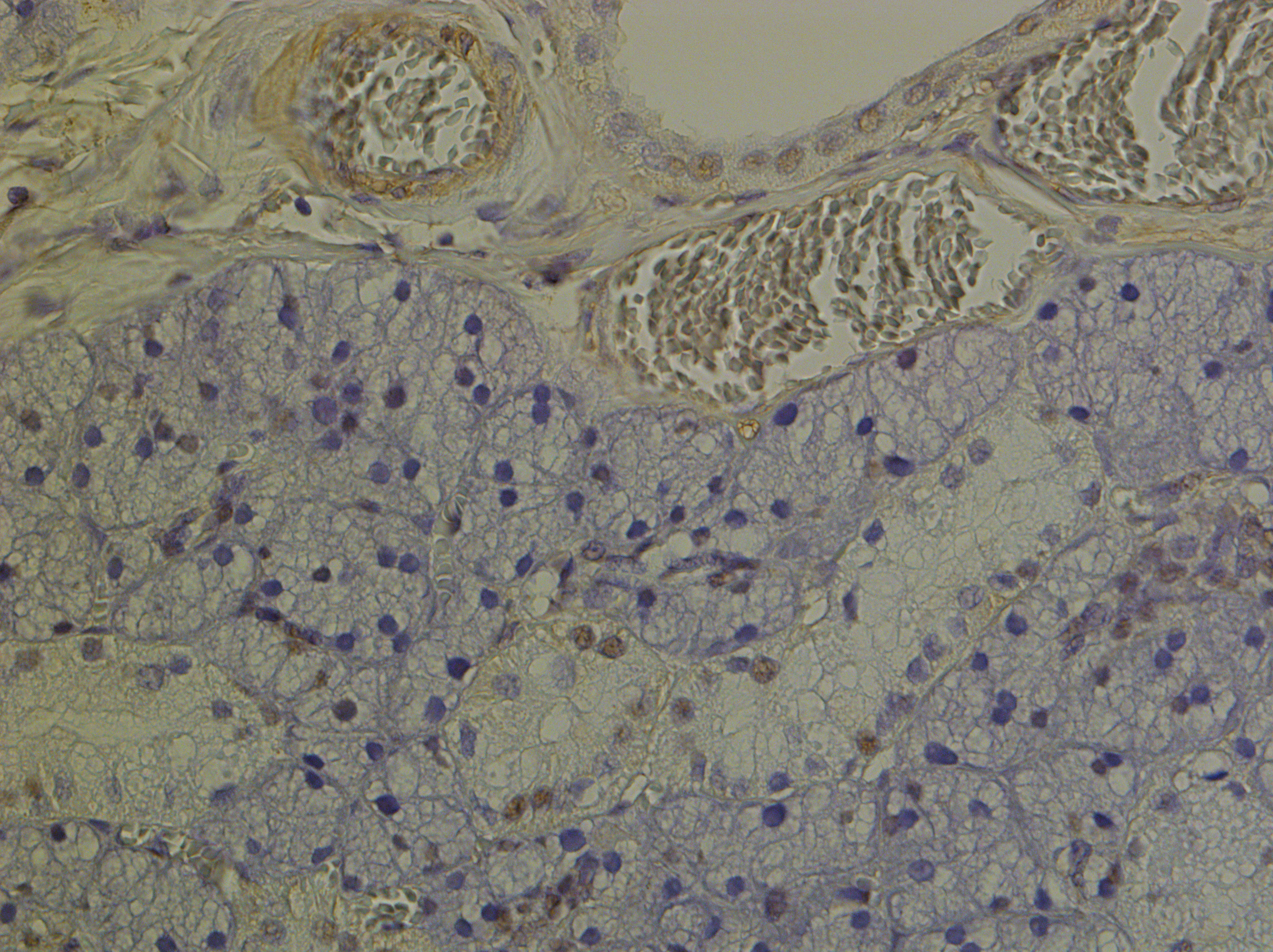

Supplement: S1 Raw file — (ZIP) [file pone.0236727.s004.zip › control4 SMG0J RAGE X40s.tif]

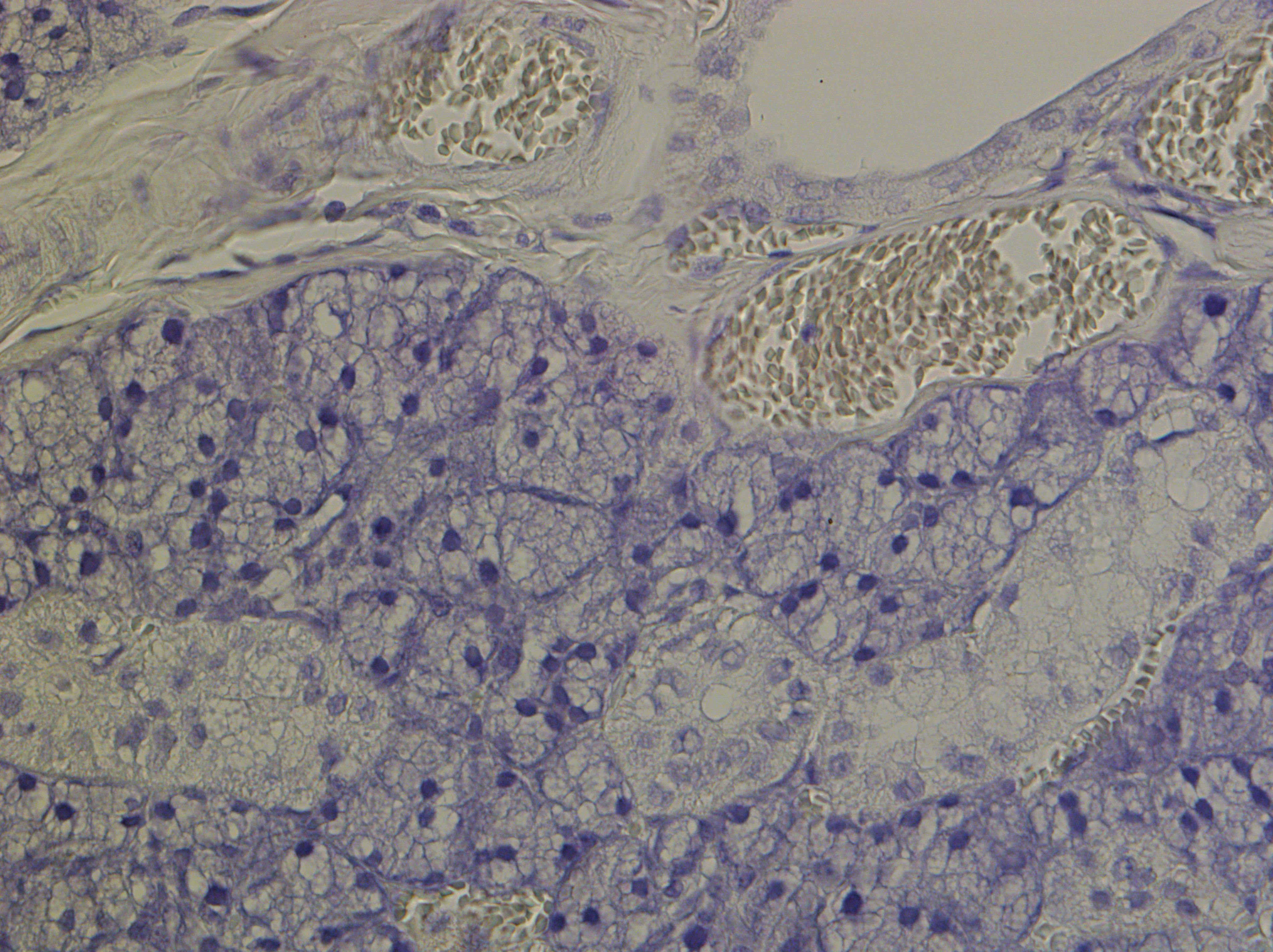

Supplement: S1 Raw file — (ZIP) [file pone.0236727.s004.zip › control4 SMG0J TNFaX40s.tif]

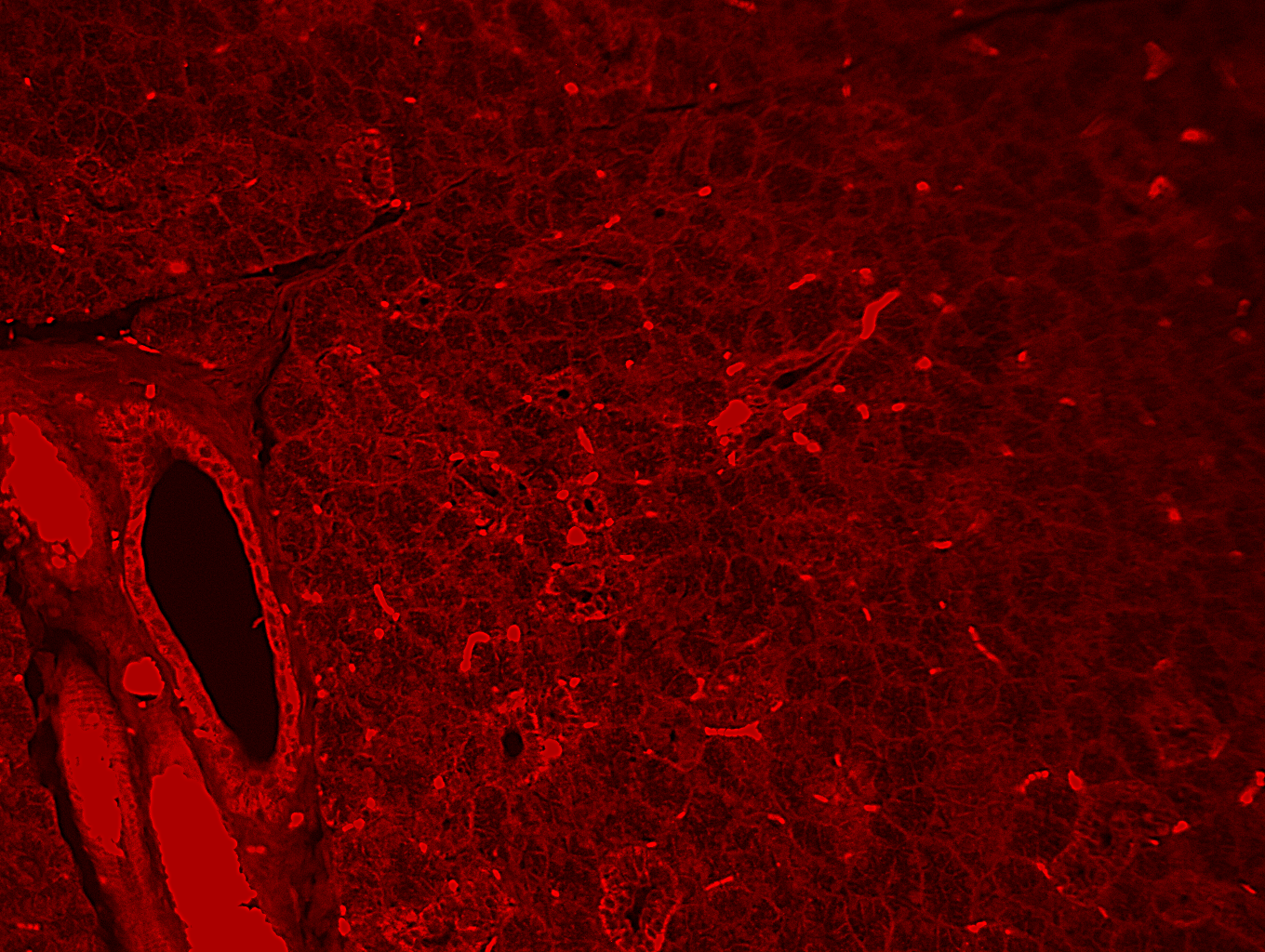

Supplement: S1 Raw file — (ZIP) [file pone.0236727.s004.zip › D34-0J-pCREB-2.tif]

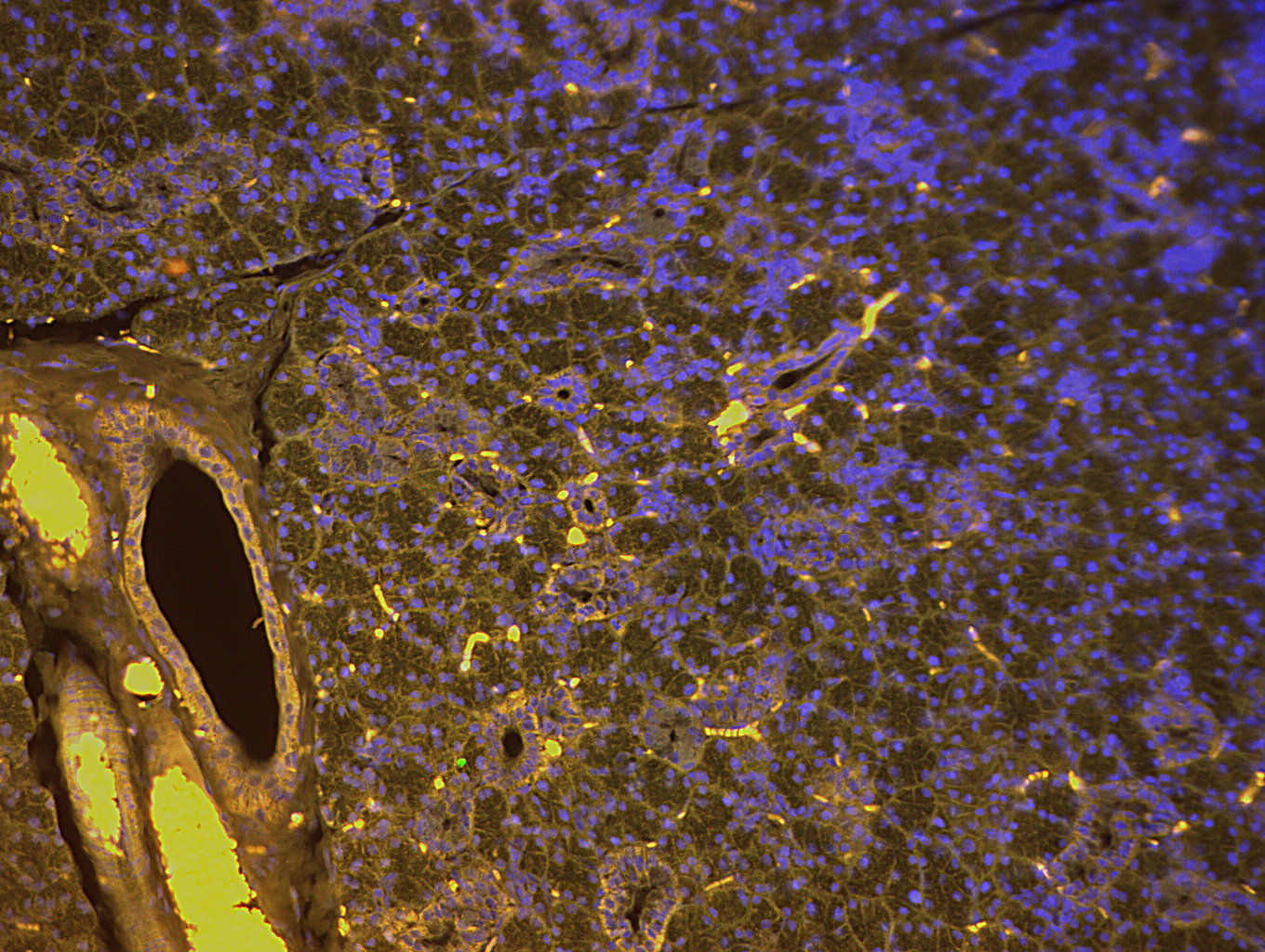

Supplement: S1 Raw file — (ZIP) [file pone.0236727.s004.zip › D34-0J-pCREBERK.tif]

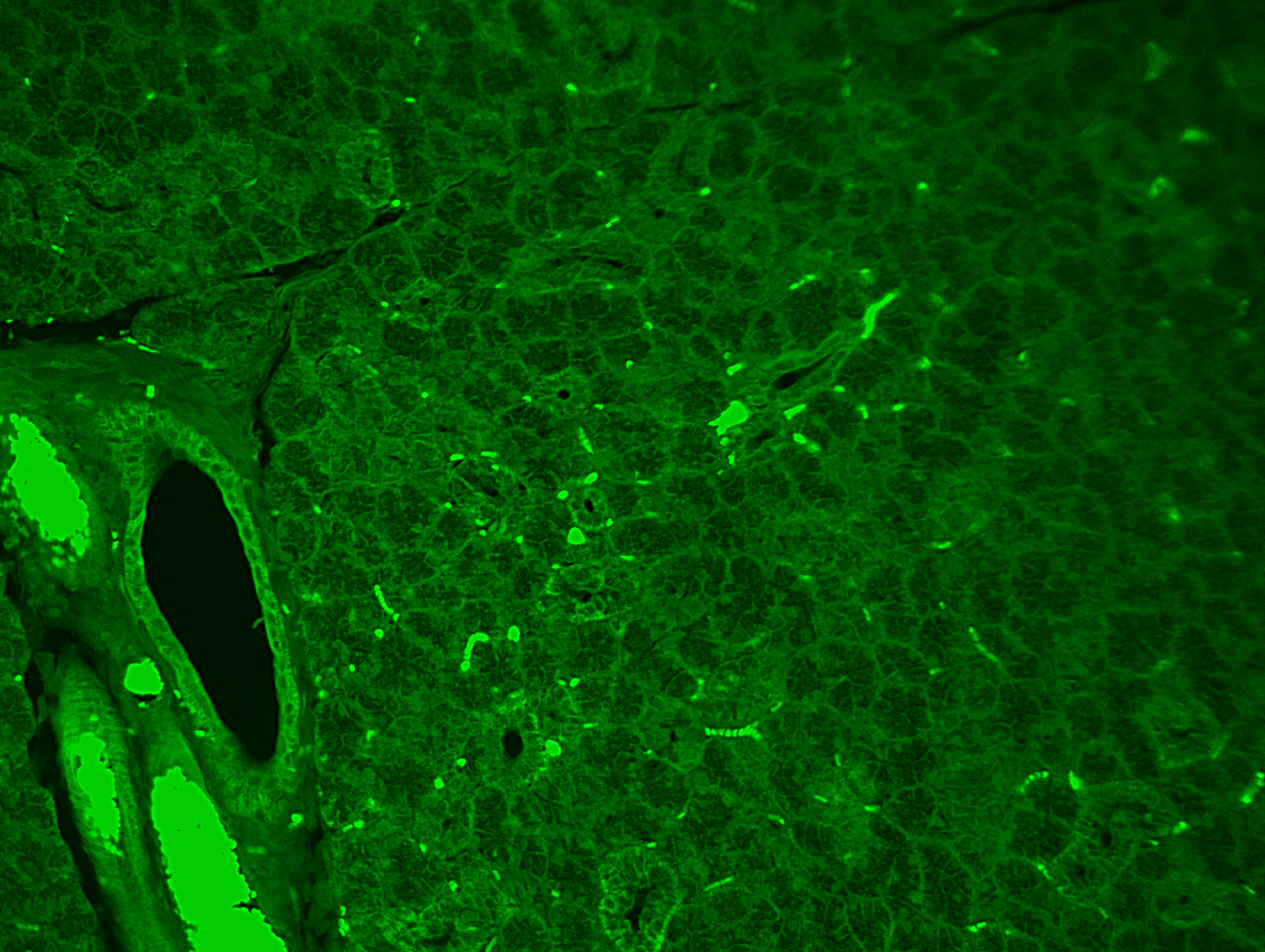

Supplement: S1 Raw file — (ZIP) [file pone.0236727.s004.zip › D34-0J-pERK.tif]

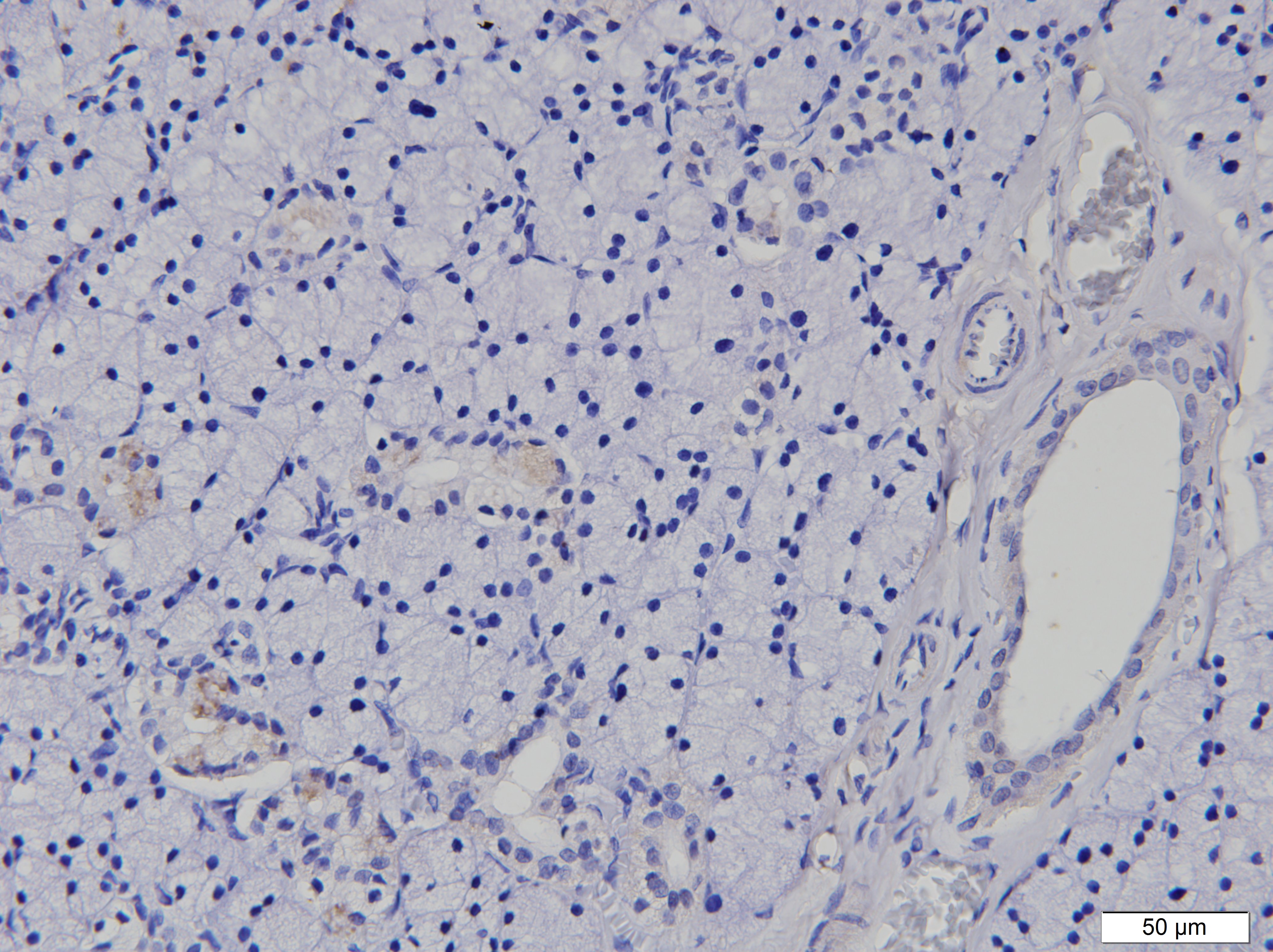

Supplement: S1 Raw file — (ZIP) [file pone.0236727.s004.zip › D34-0J-SM cAMPX40.jpg]

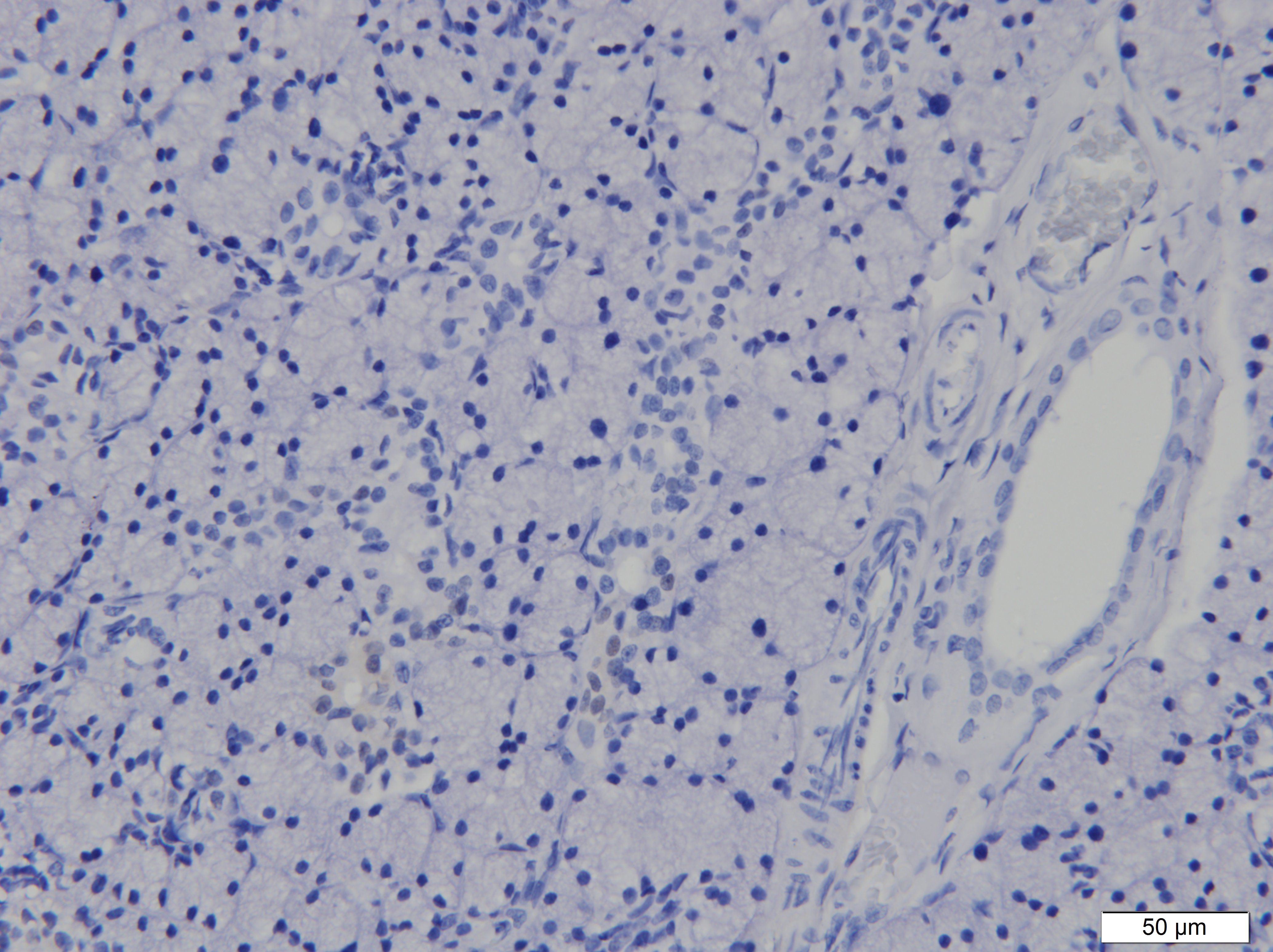

Supplement: S1 Raw file — (ZIP) [file pone.0236727.s004.zip › D34-0J-SM phospho ERKX40-10.jpg]

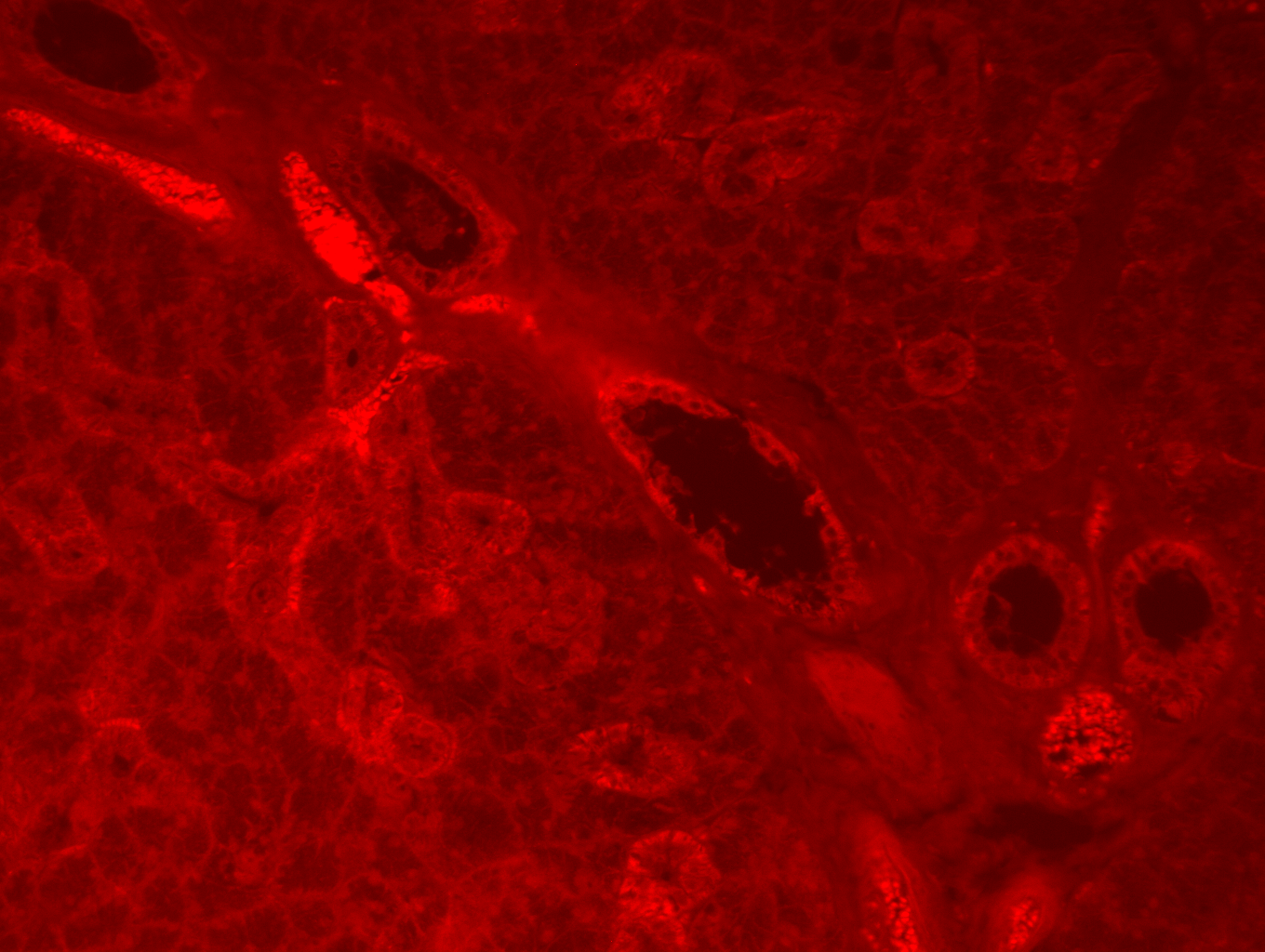

Supplement: S1 Raw file — (ZIP) [file pone.0236727.s004.zip › D37-20J-pCREB-2.TIF]

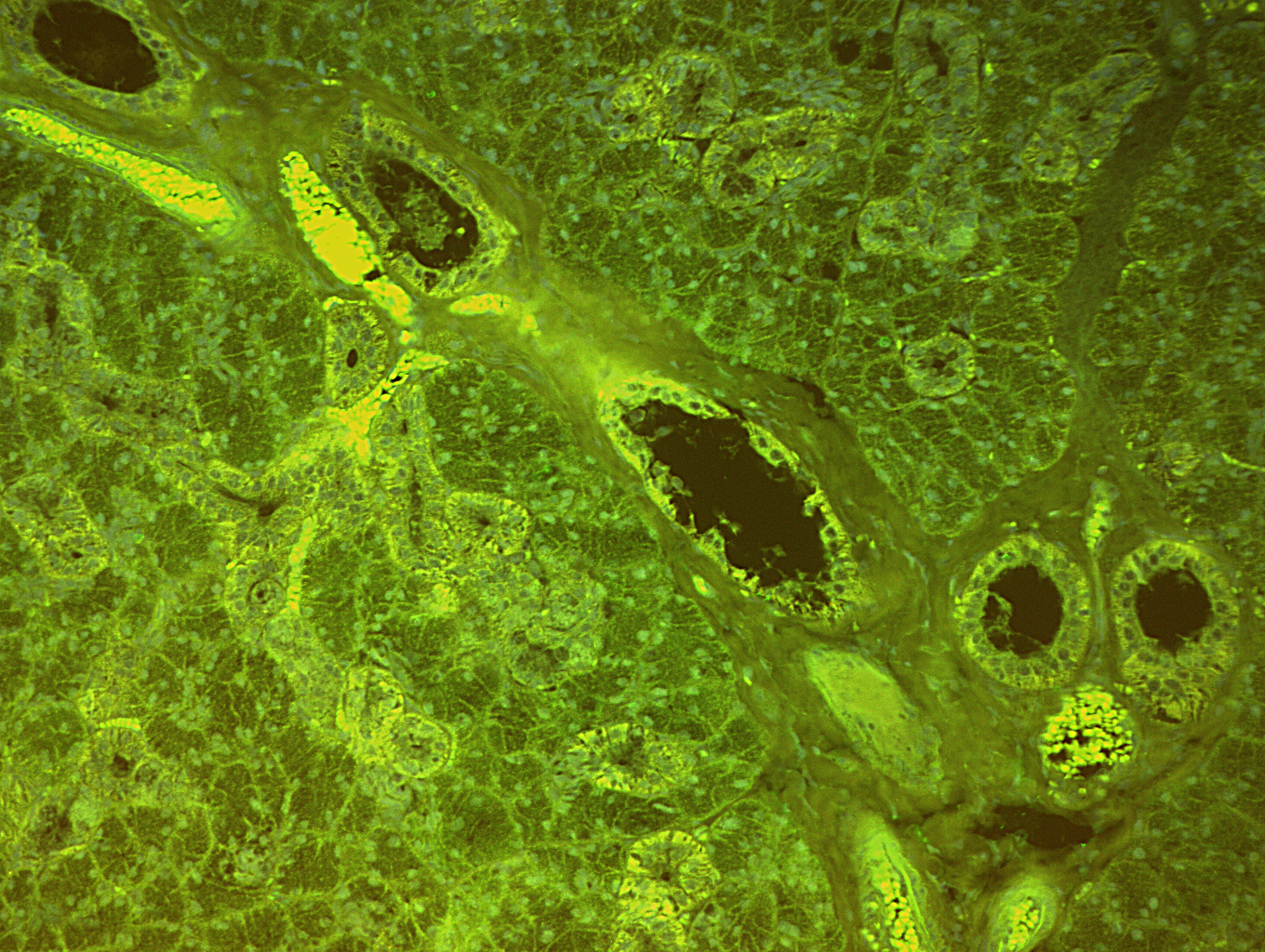

Supplement: S1 Raw file — (ZIP) [file pone.0236727.s004.zip › D37-20J-pCREBERK.tif]

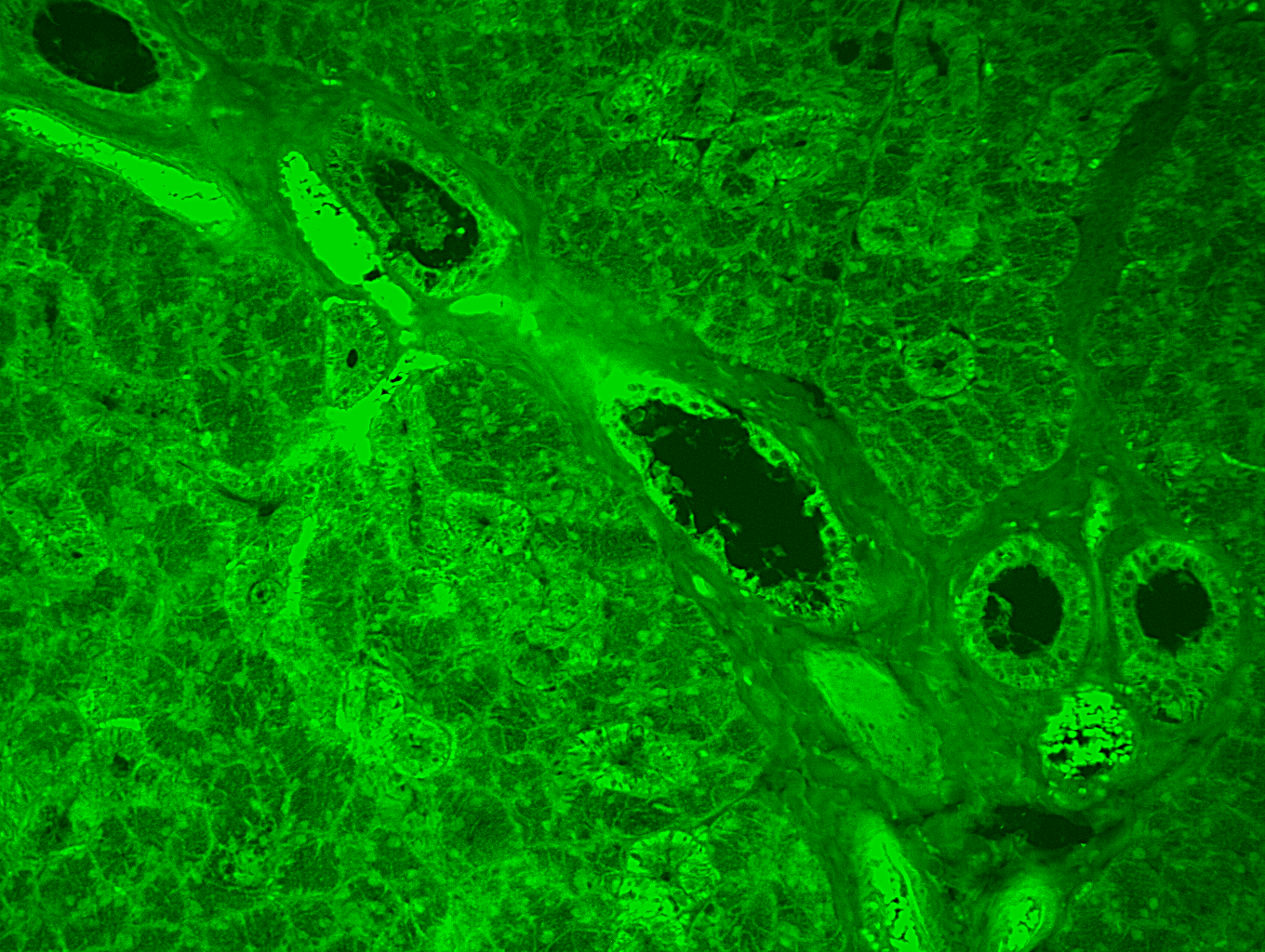

Supplement: S1 Raw file — (ZIP) [file pone.0236727.s004.zip › D37-20J-pERK.tif]

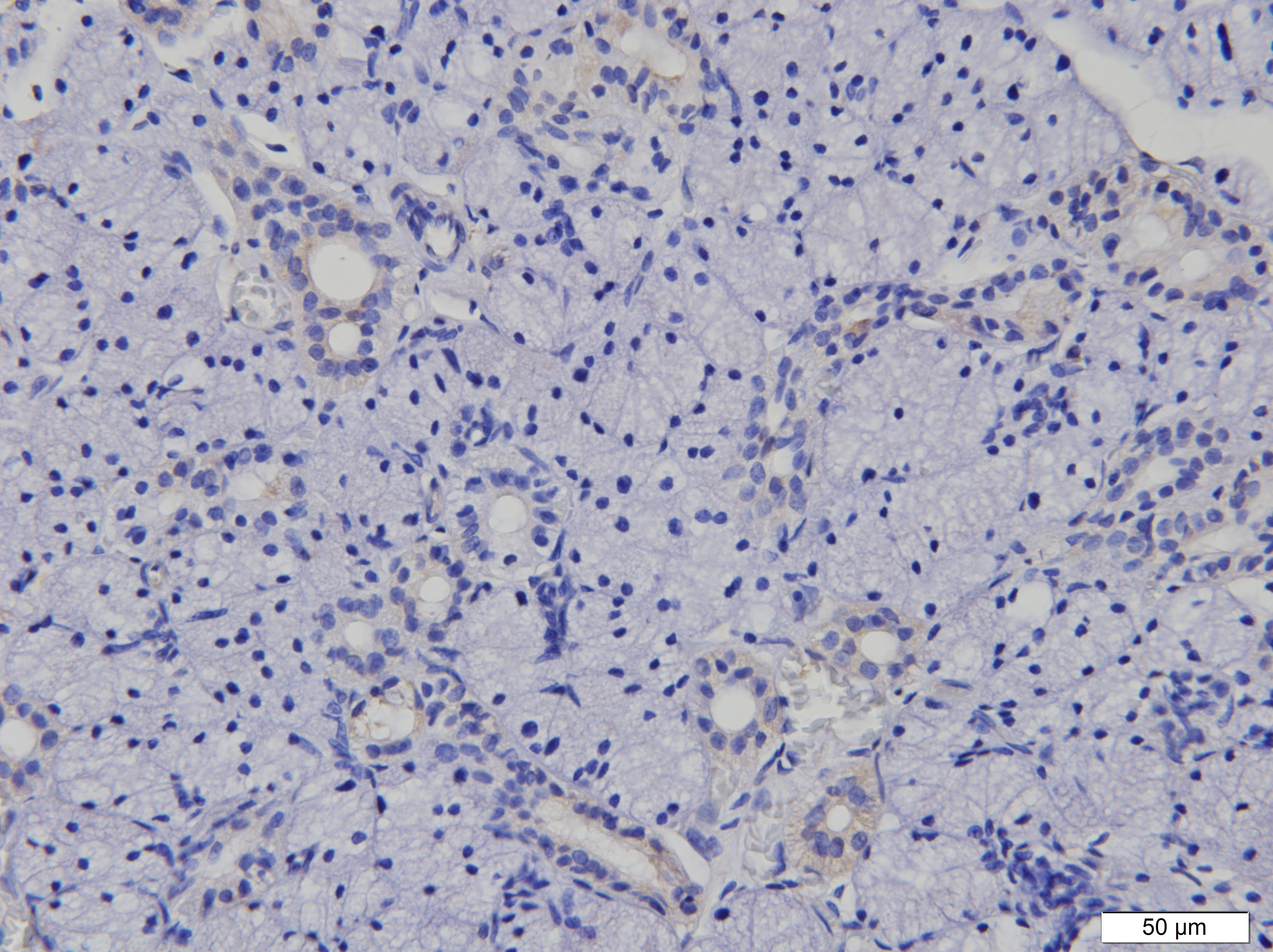

Supplement: S1 Raw file — (ZIP) [file pone.0236727.s004.zip › D37-20J-SM cAMPX40-10.jpg]

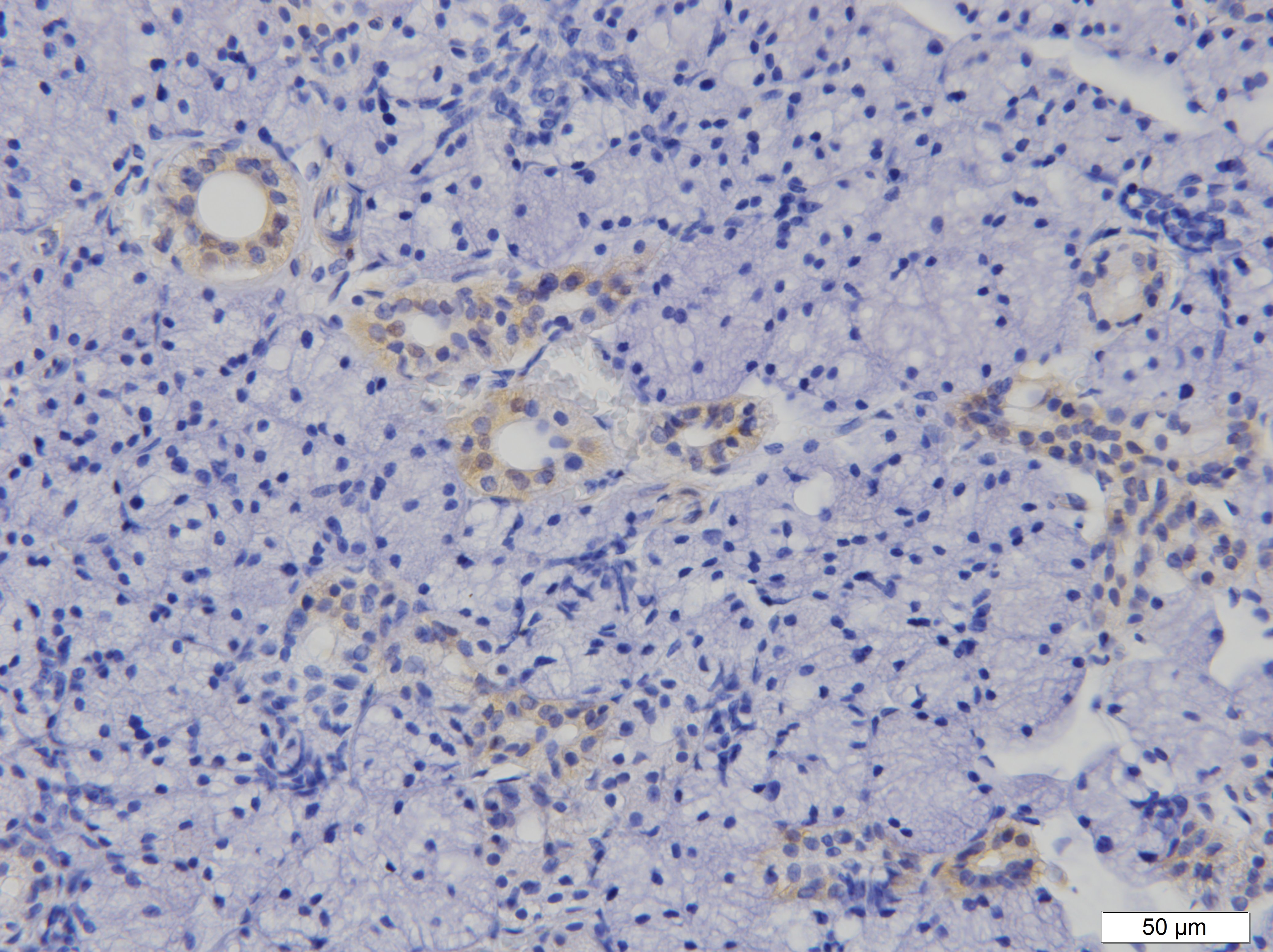

Supplement: S1 Raw file — (ZIP) [file pone.0236727.s004.zip › D37-20J-SM phosphoERKX40-1.jpg]

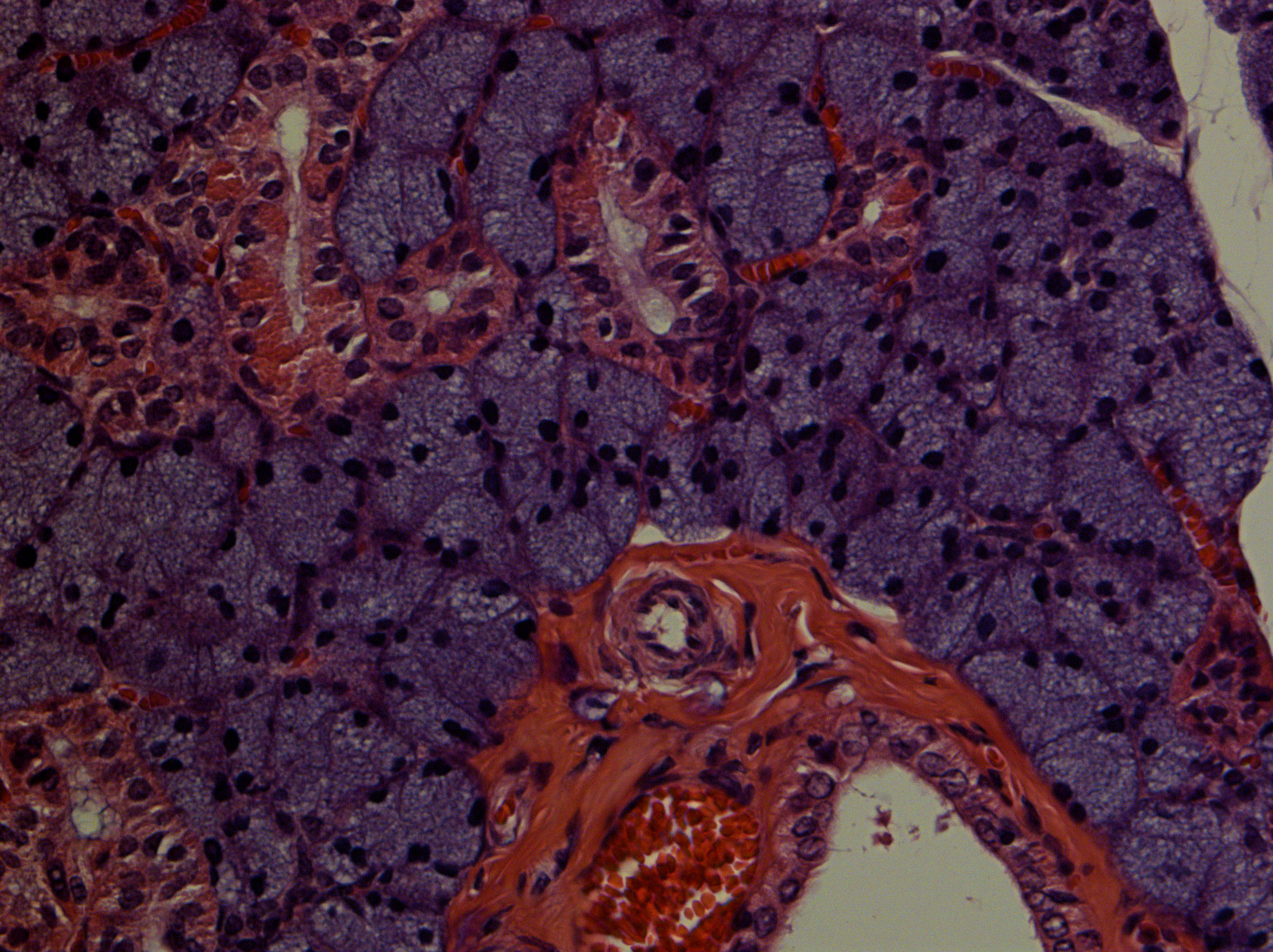

Supplement: S1 Raw file — (ZIP) [file pone.0236727.s004.zip › diabetes1SMG20J HEx40s.tif]

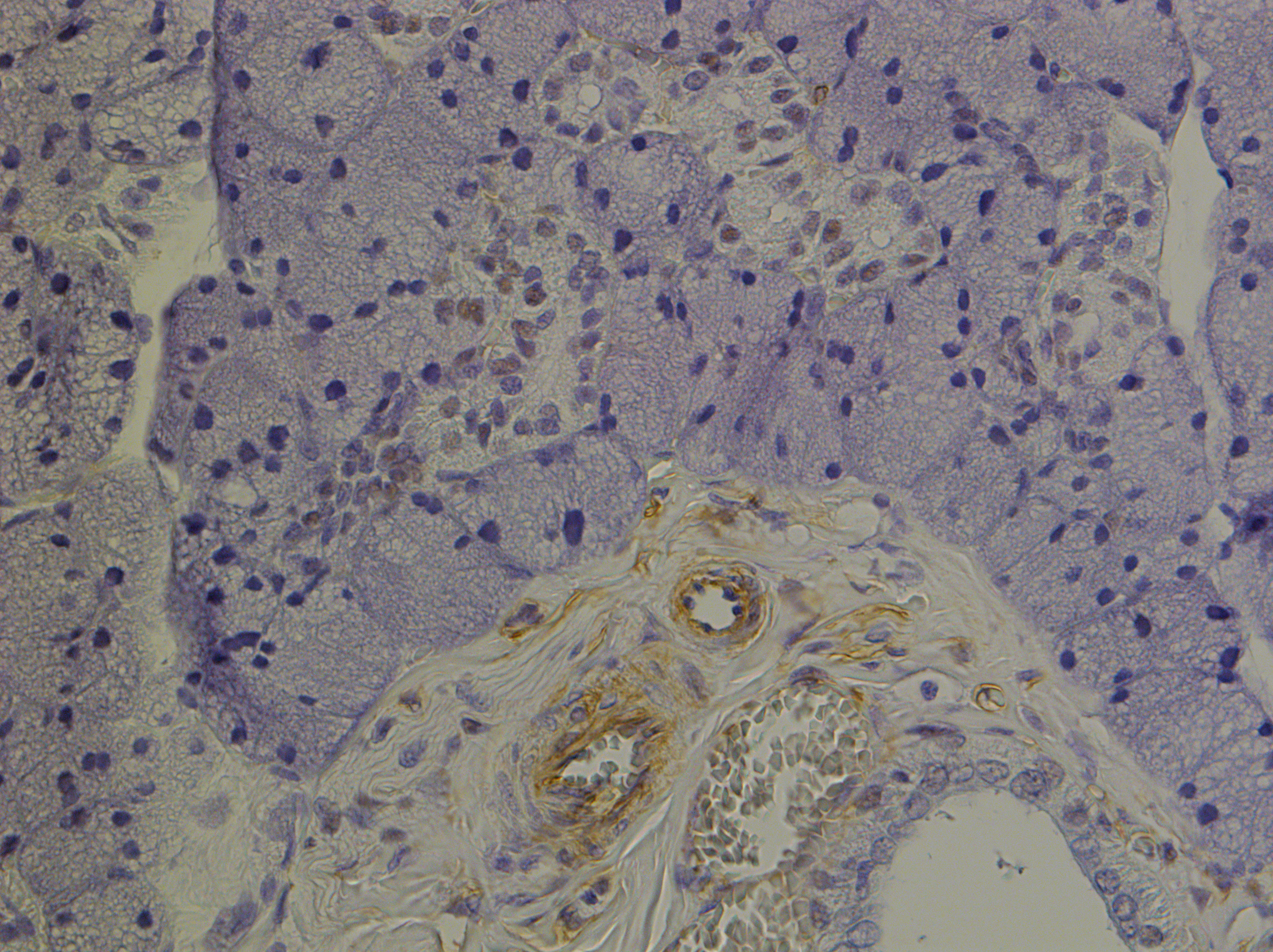

Supplement: S1 Raw file — (ZIP) [file pone.0236727.s004.zip › diabetes1SMG20J HMGB1x40s.tif]

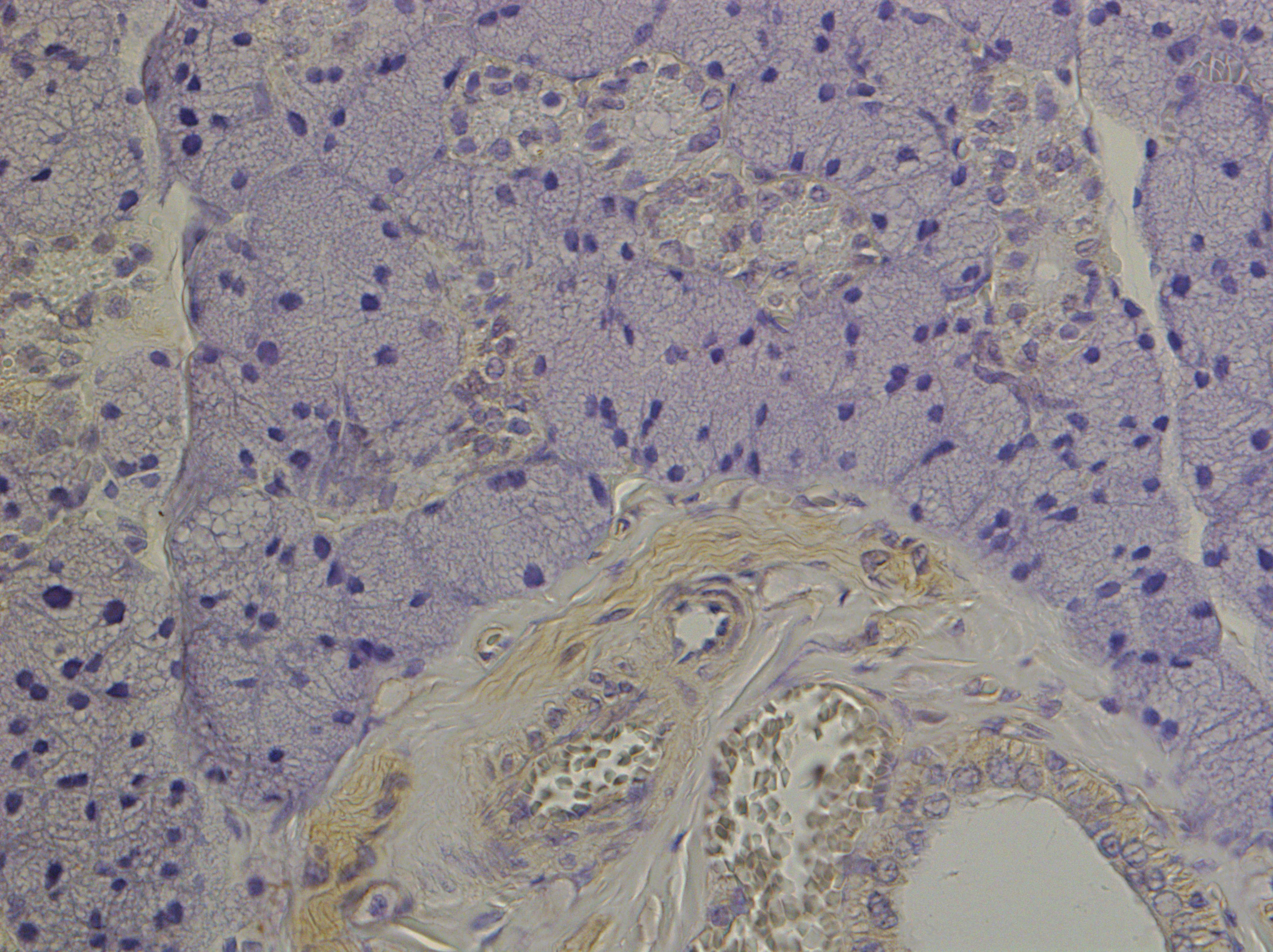

Supplement: S1 Raw file — (ZIP) [file pone.0236727.s004.zip › diabetes1SMG20J phosphoNFkB x40s.tif]

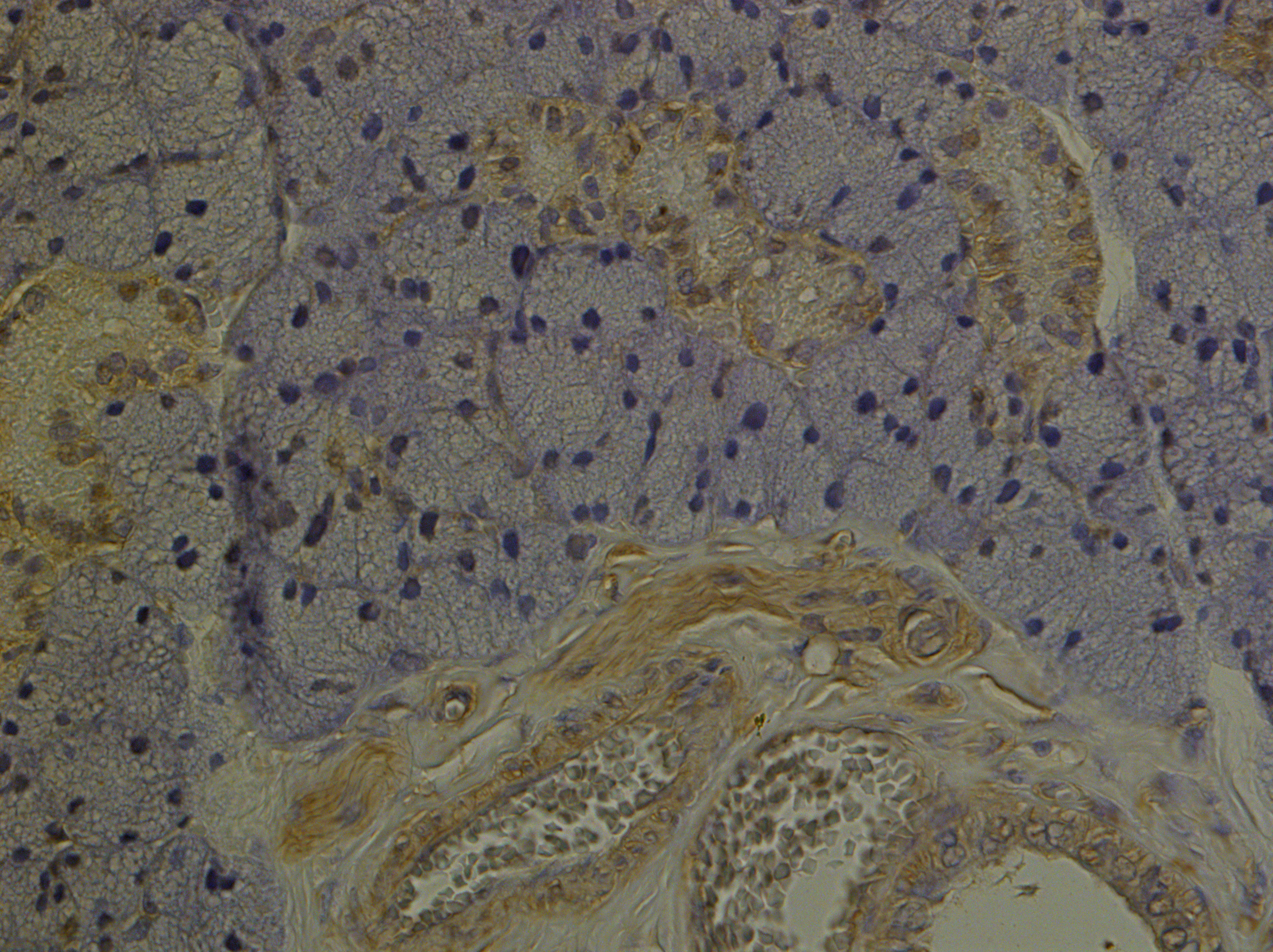

Supplement: S1 Raw file — (ZIP) [file pone.0236727.s004.zip › diabetes1SMG20J RAGEx40s.tif]

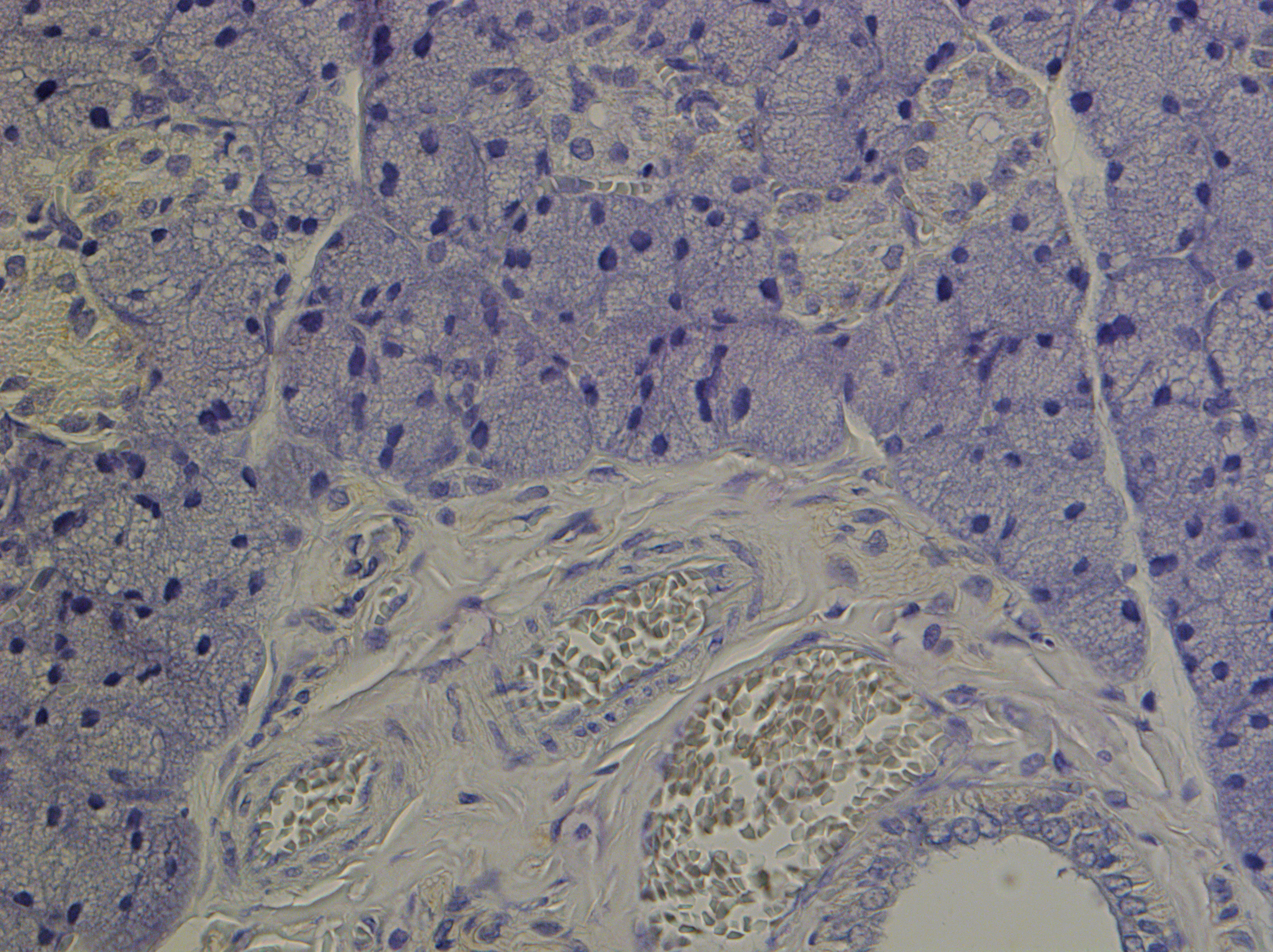

Supplement: S1 Raw file — (ZIP) [file pone.0236727.s004.zip › diabetes1SMG20J TNFax40s.tif]

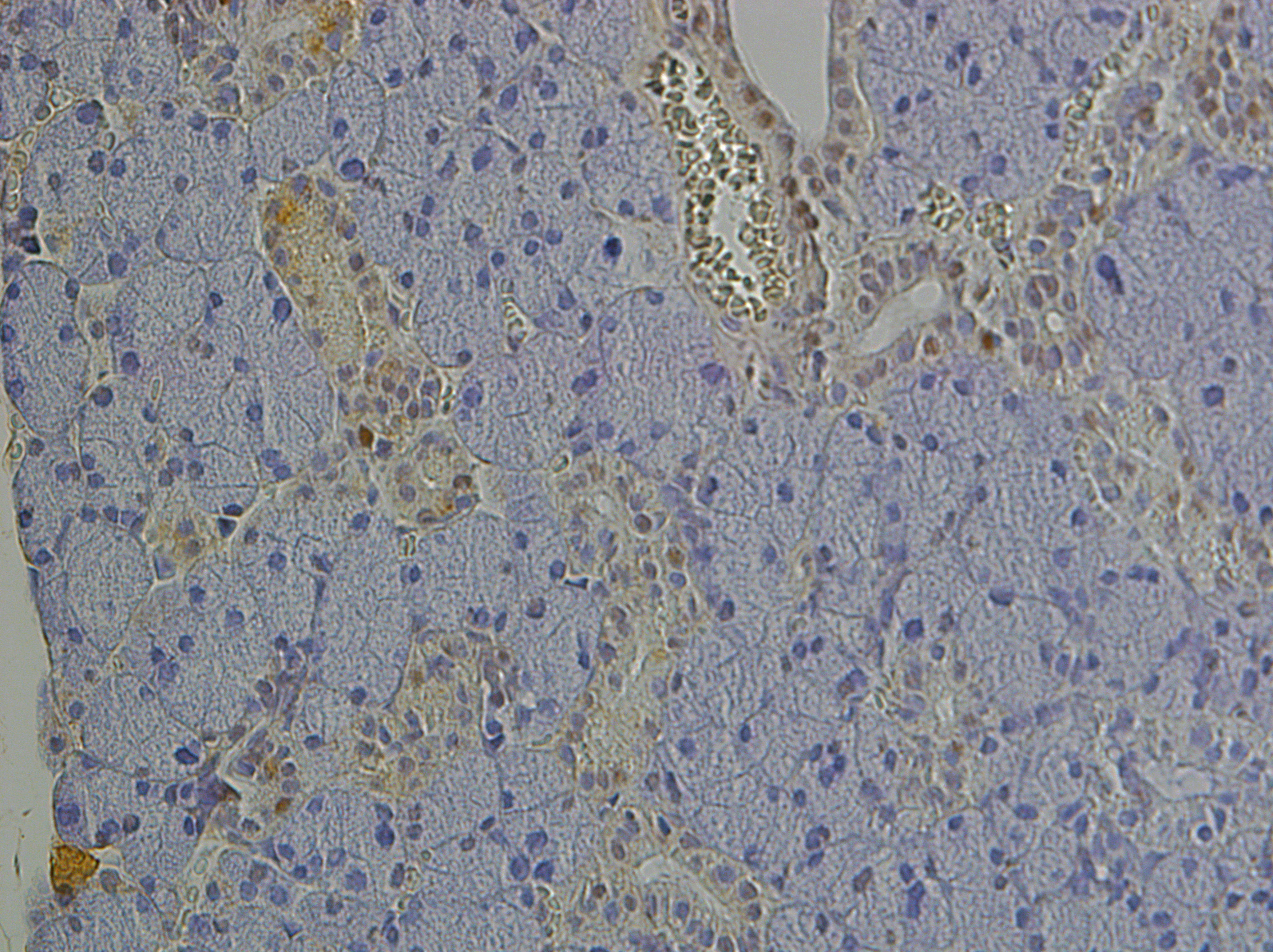

Supplement: S1 Raw file — (ZIP) [file pone.0236727.s004.zip › Diabetes4 SMG 20J pCREB X40s.tif]

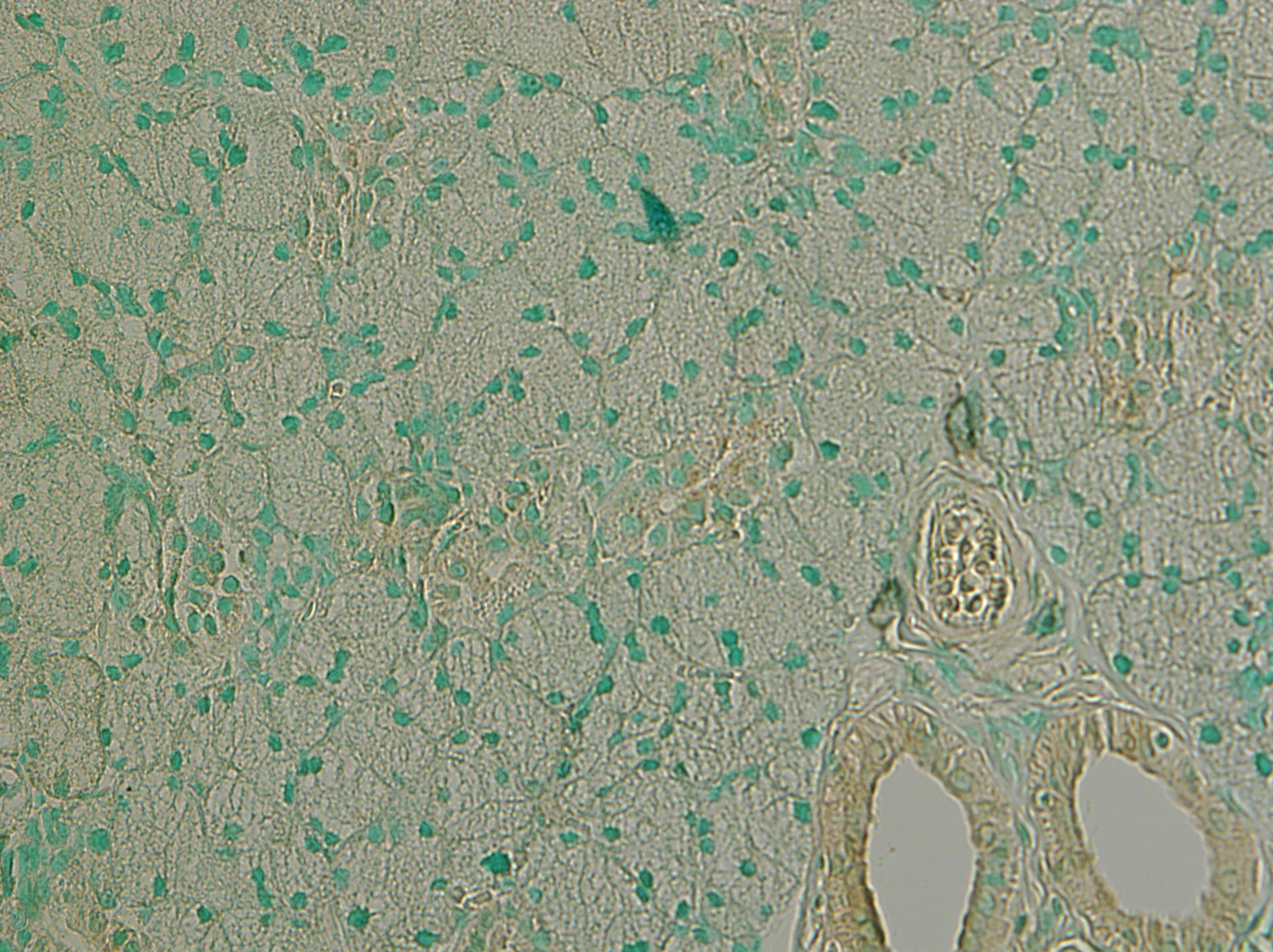

Supplement: S1 Raw file — (ZIP) [file pone.0236727.s004.zip › Diabetes4 SMG 20J TUNEL X40s.tif]

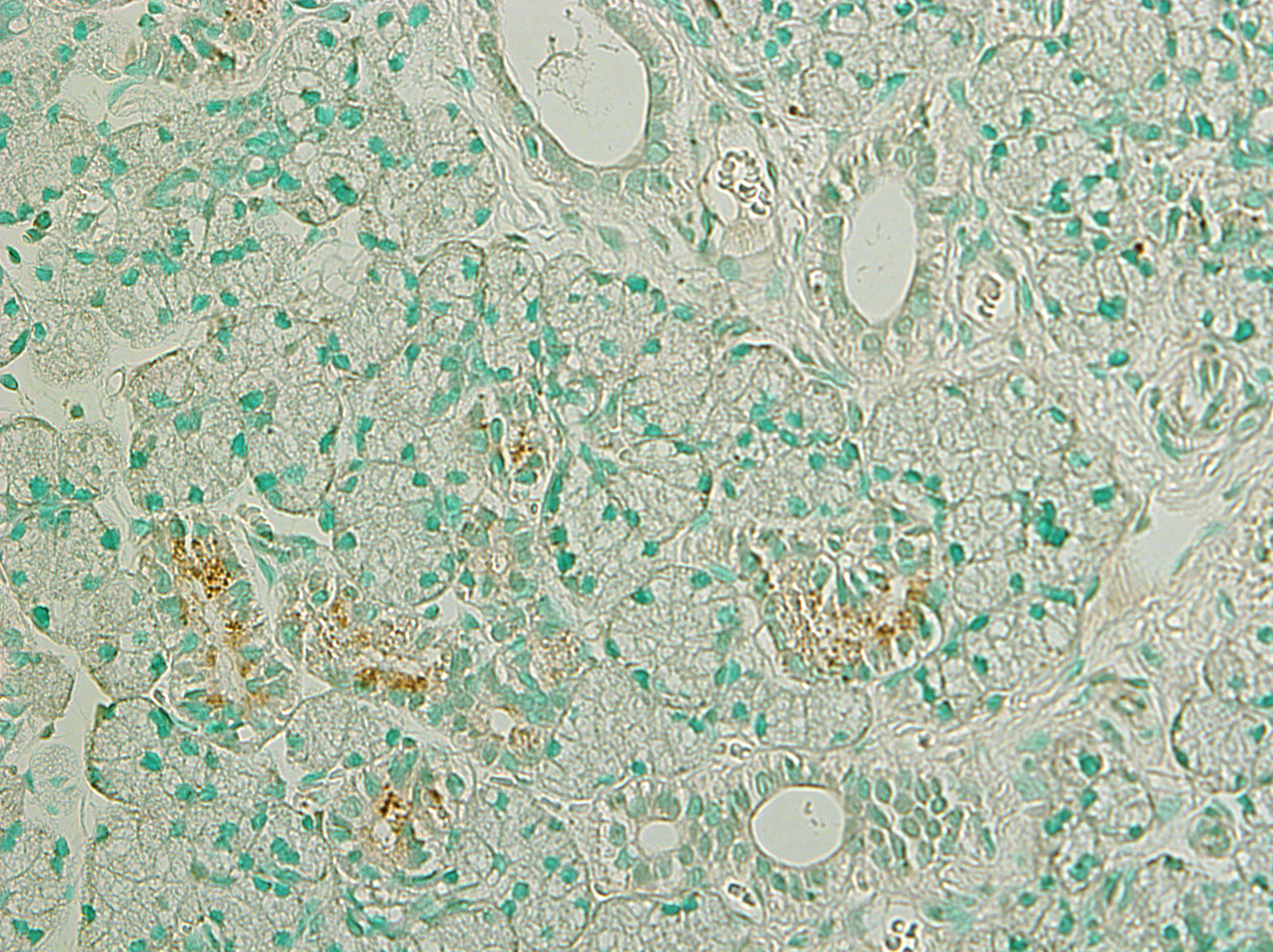

Supplement: S1 Raw file — (ZIP) [file pone.0236727.s004.zip › Diabetes6 SMG 0J TUNEL X40-1.tif]

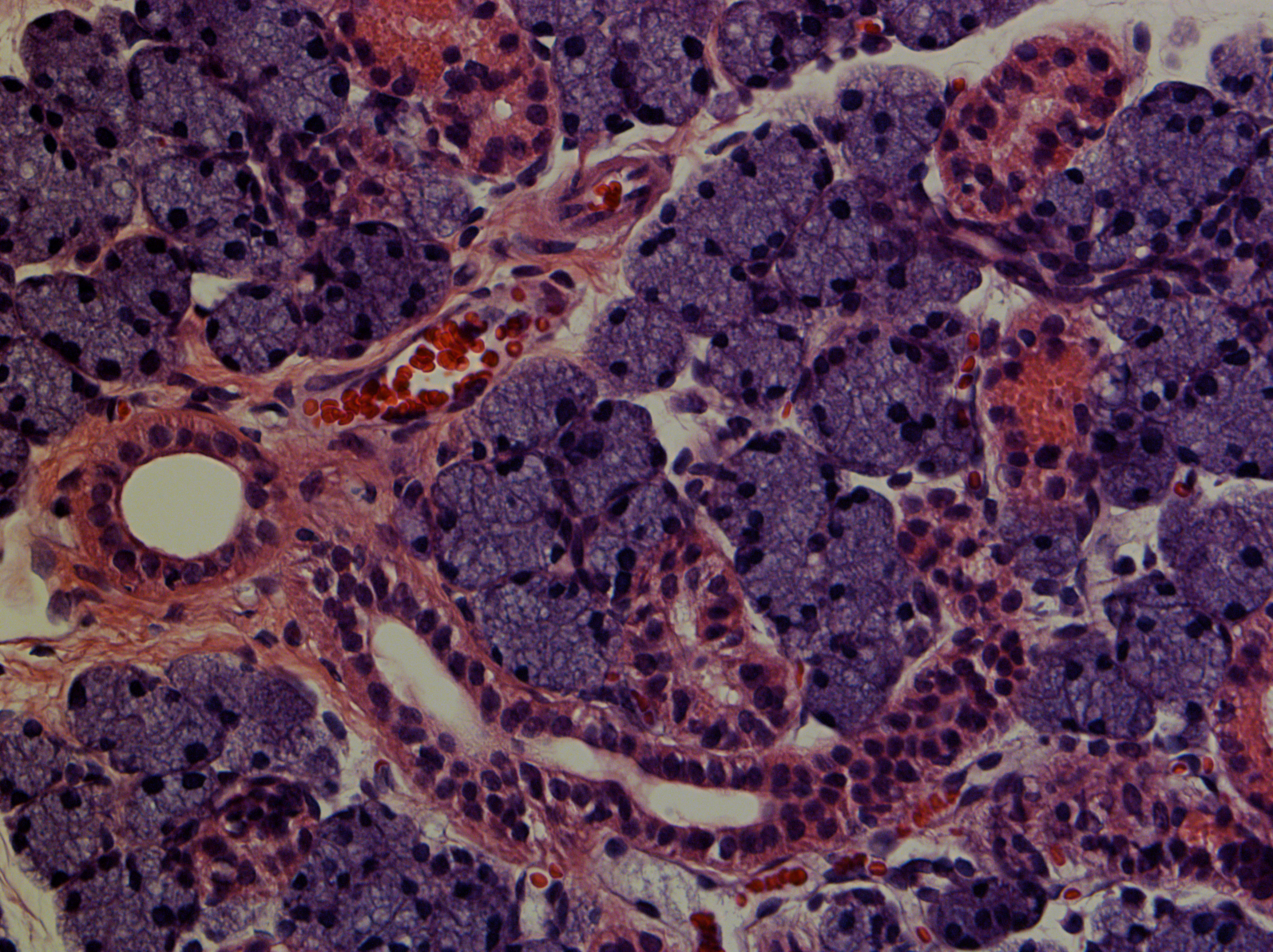

Supplement: S1 Raw file — (ZIP) [file pone.0236727.s004.zip › diabetes6SMG0J HE x40s.tif]

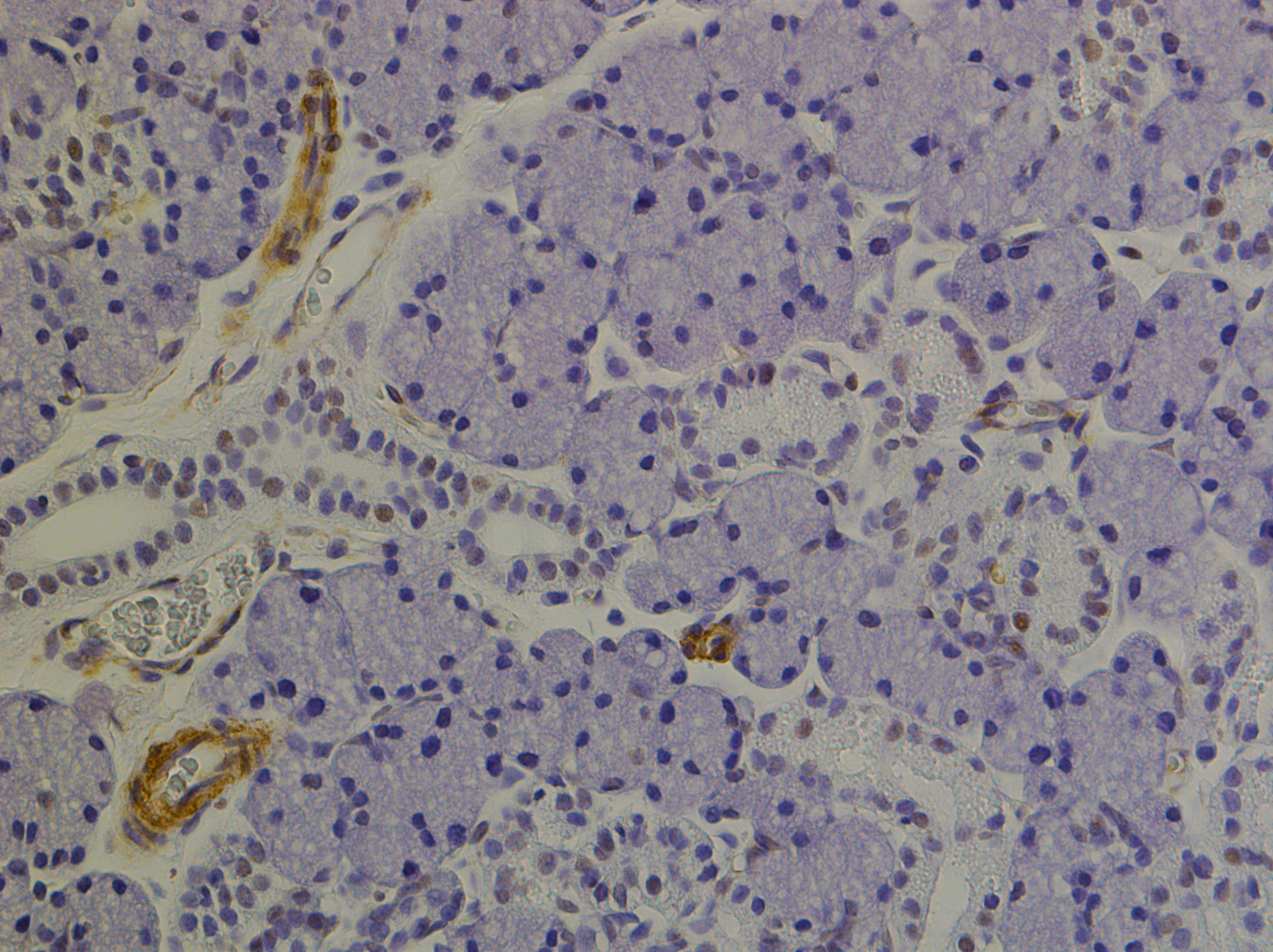

Supplement: S1 Raw file — (ZIP) [file pone.0236727.s004.zip › diabetes6SMG0J HMGB1 x40s.tif]

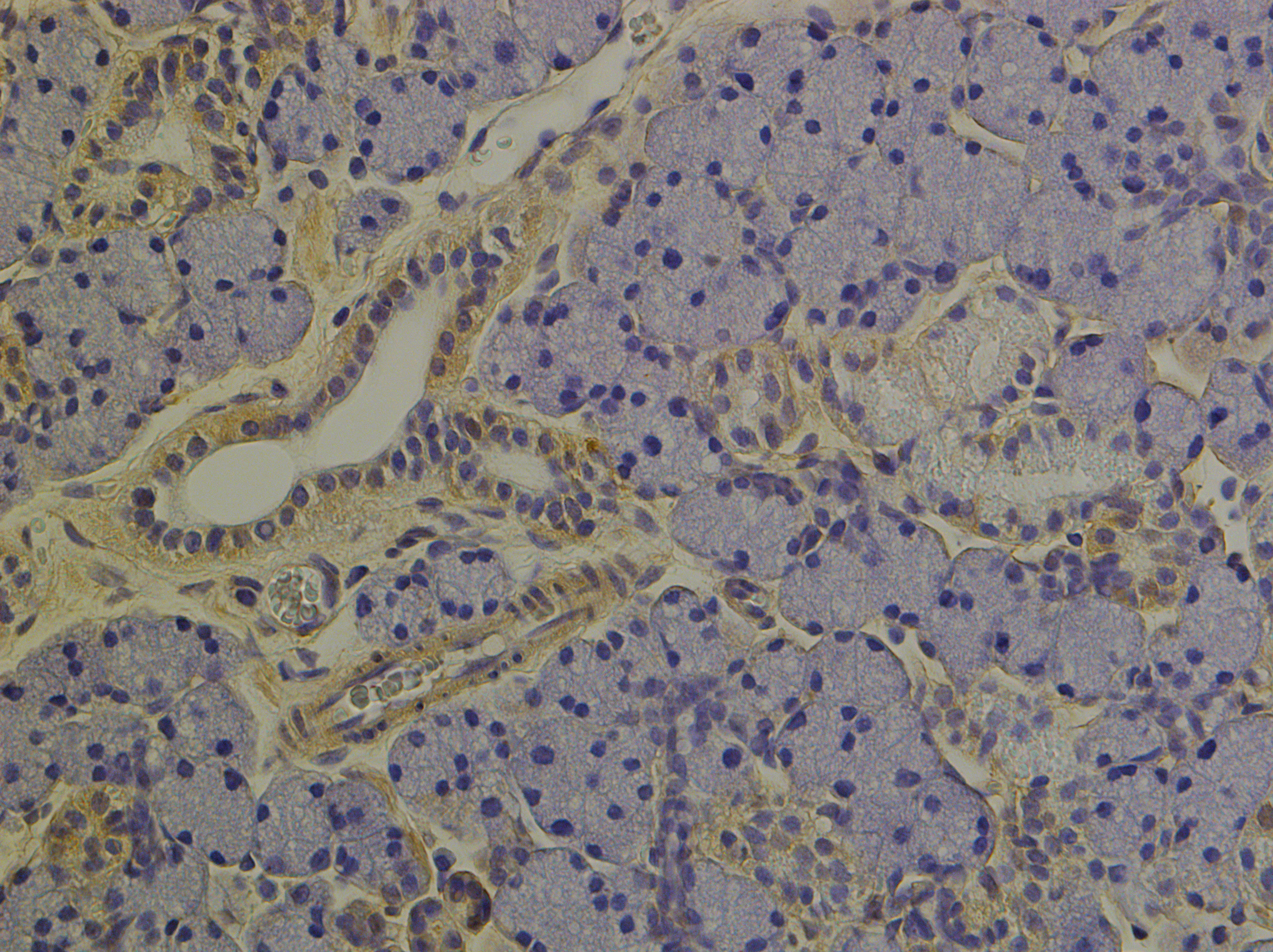

Supplement: S1 Raw file — (ZIP) [file pone.0236727.s004.zip › diabetes6SMG0J phosphoNFkB x40s.tif]

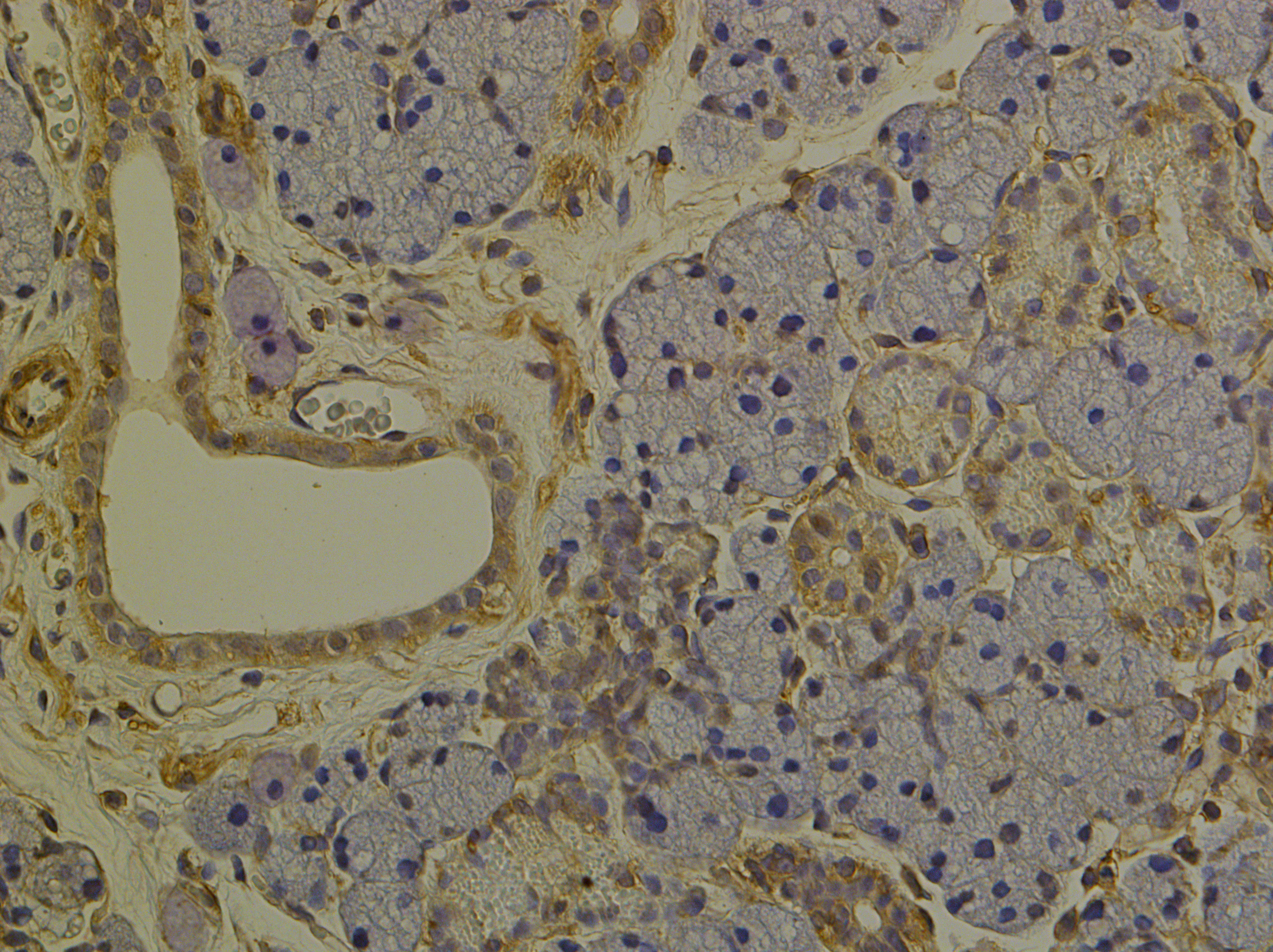

Supplement: S1 Raw file — (ZIP) [file pone.0236727.s004.zip › diabetes6SMG0J RAGE x40s.tif]

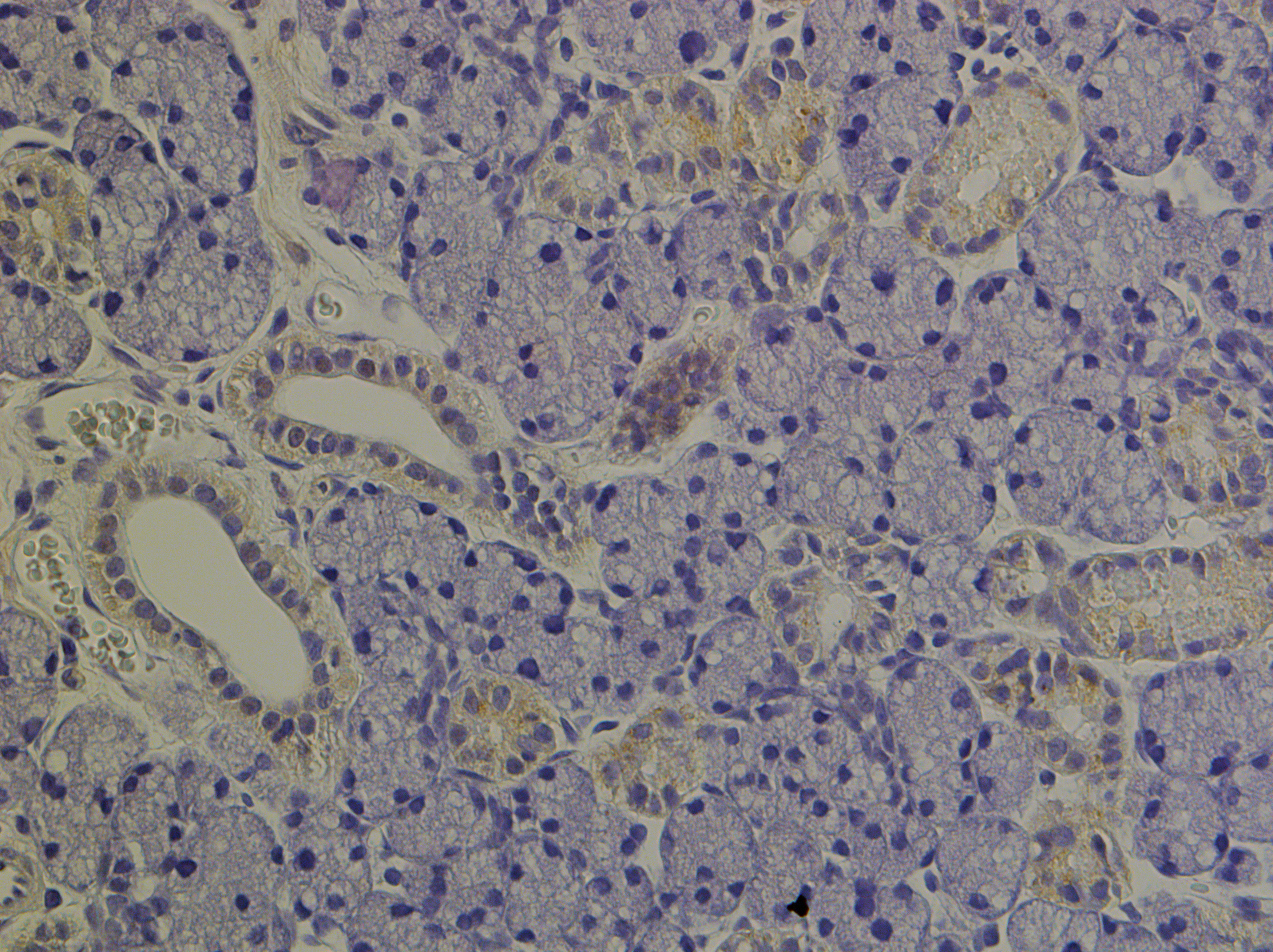

Supplement: S1 Raw file — (ZIP) [file pone.0236727.s004.zip › diabetes6SMG0J TNFa x40s.tif]

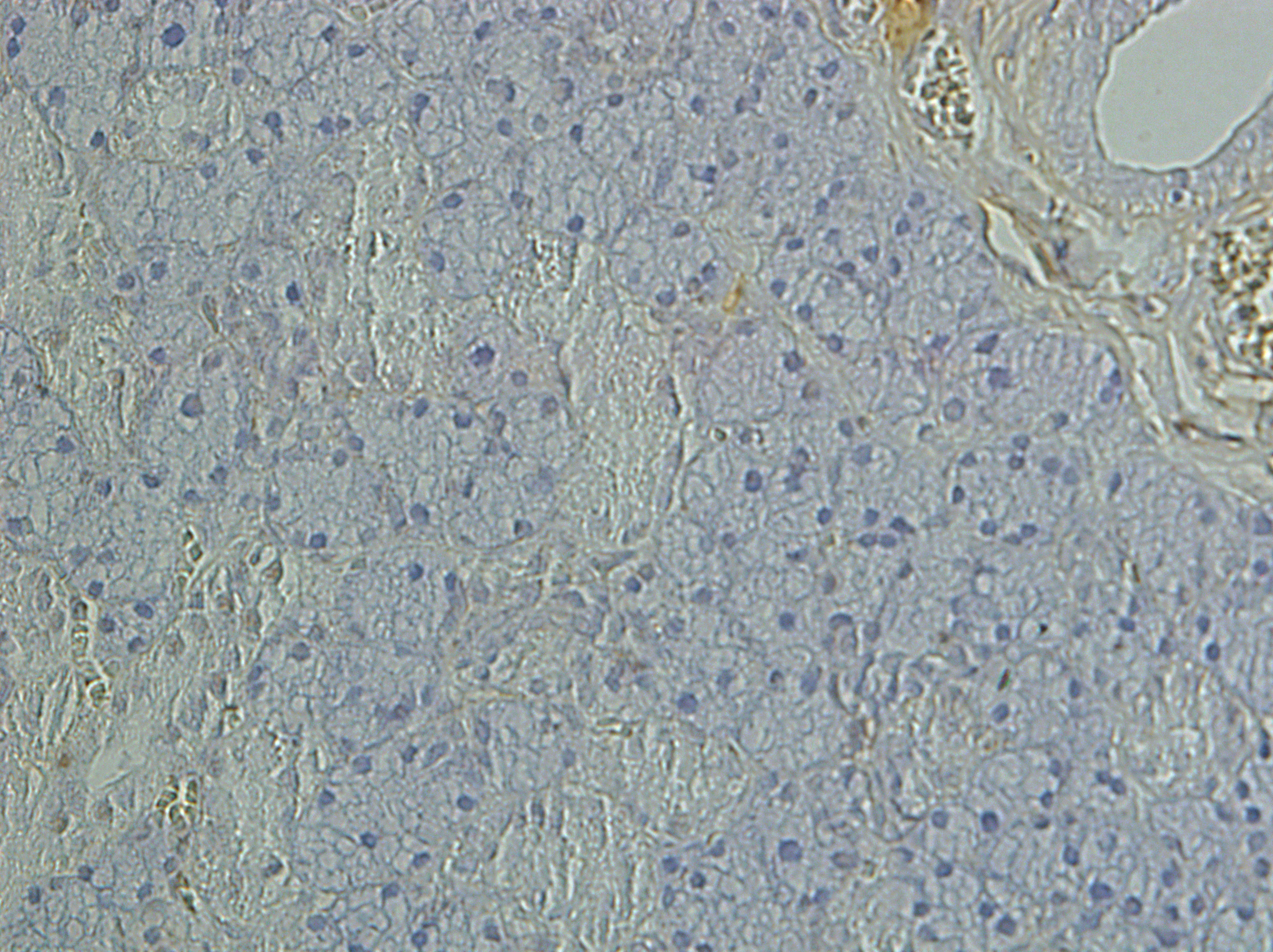

Supplement: S1 Raw file — (ZIP) [file pone.0236727.s004.zip › Diabetes9 SMG 0J pCREB X40s.tif]

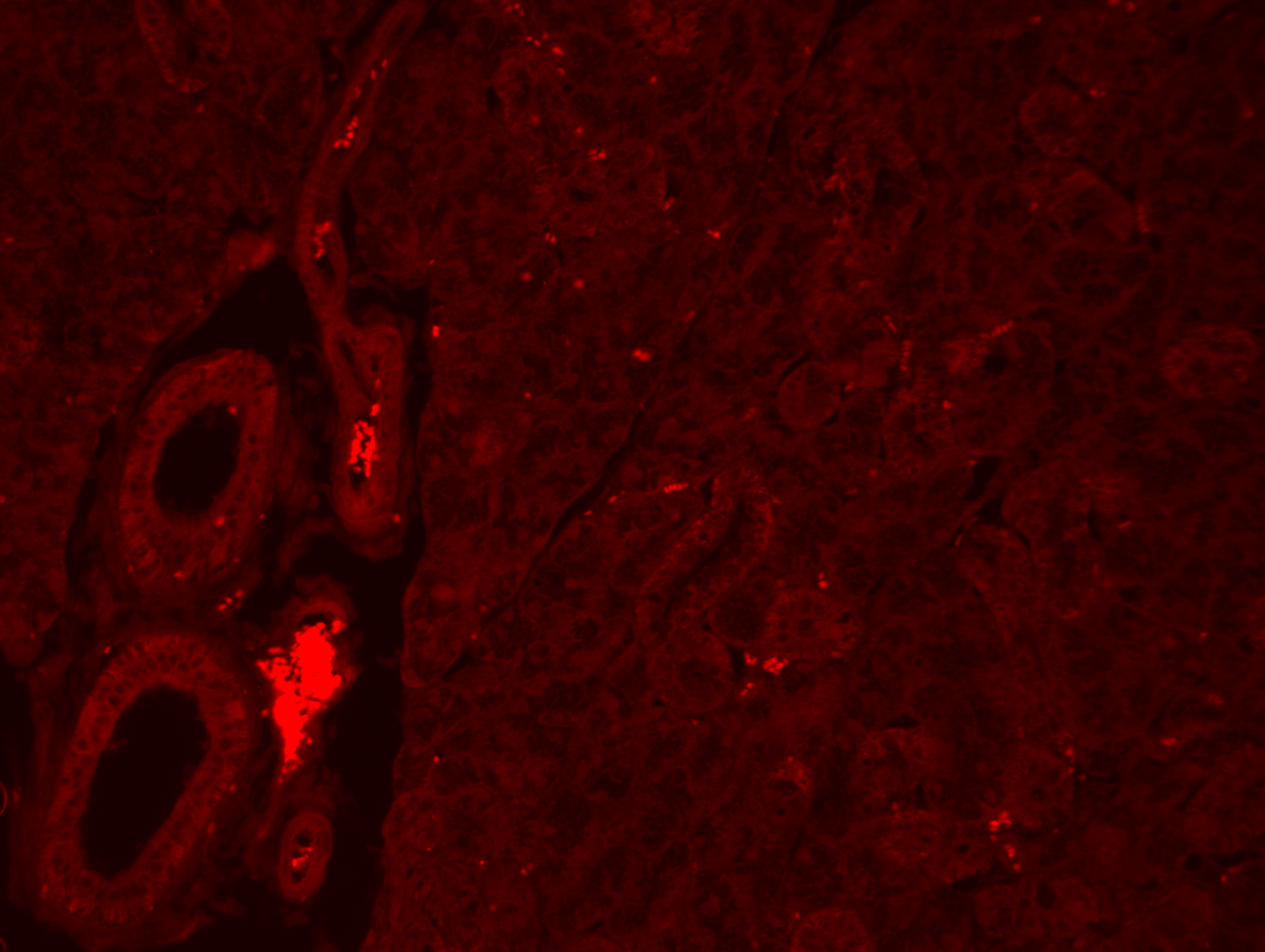

Supplement: S1 Raw file — (ZIP) [file pone.0236727.s004.zip › pH 6.0_CH2.TIF]
