# Supplementary material for: Early effect of laser irradiation in signaling pathways of diabetic rat submandibular salivary glands
Source: PLoS One. 2020 Aug 4;15(8):e0236727. doi: 10.1371/journal.pone.0236727 (PMC7402516; doi:10.1371/journal.pone.0236727)
Supplement: S1 Table — Body weight on the 1st experimental day (initial) and in the 30th day (final). Blood glucose levels were measured in the 3rd experimental day (initial) and in the day of the sacrifice (final), after 12h-fasting (n = 8/group). Data are presented as mean ± SD; * P < 0.05 vs control group (C0); # P < 0.05 vs respective diabetic group (D0); ⱡ P < 0.05 vs initial time in the same group. (DOCX) [file pone.0236727.s003.docx]

|  | **Body weight (g)** | | **Blood Glucose (mg/dl)** | |
| --- | --- | --- | --- | --- |
| **Group** | **Initial** | **Final** | **Initial** | **Final** |
| **C0** | 180 ± 26 | 189 ± 24 | 109 ± 45 | 146 ± 49 ⱡ |
| **D0** | 209 ± 6 * | 161 ± 15 * ⱡ | 475 ± 89 * | 501± 101 * |
| **D20** | 211 ± 9 * | 156 ± 22 * ⱡ | 414 ± 118 * | 321 ± 117 * # |

**S1 Table. LPLI decreased diabetic animals blood glucose levels.**  Body weight on the 1^st^ experimental day (initial) and in the 30^th^ day (final). Blood glucose levels were measured in the 3^rd^ experimental day (initial) and in the day of the sacrifice (final), after 12h-fasting (n=8/group). Data are presented as mean ± SD; * P < 0.05 vs control group (C0); # P < 0.05 vs respective diabetic group (D0); ⱡ P < 0.05 vs initial time in the same group.
