# Supplementary material for: Early effect of laser irradiation in signaling pathways of diabetic rat submandibular salivary glands
Source: PLoS One. 2020 Aug 4;15(8):e0236727. doi: 10.1371/journal.pone.0236727 (PMC7402516; doi:10.1371/journal.pone.0236727)
Supplement: S1 Raw file — (ZIP) [file pone.0236727.s004.zip › 070620-WB beta-actina.pdf]

WB- B-actina 37 KDa

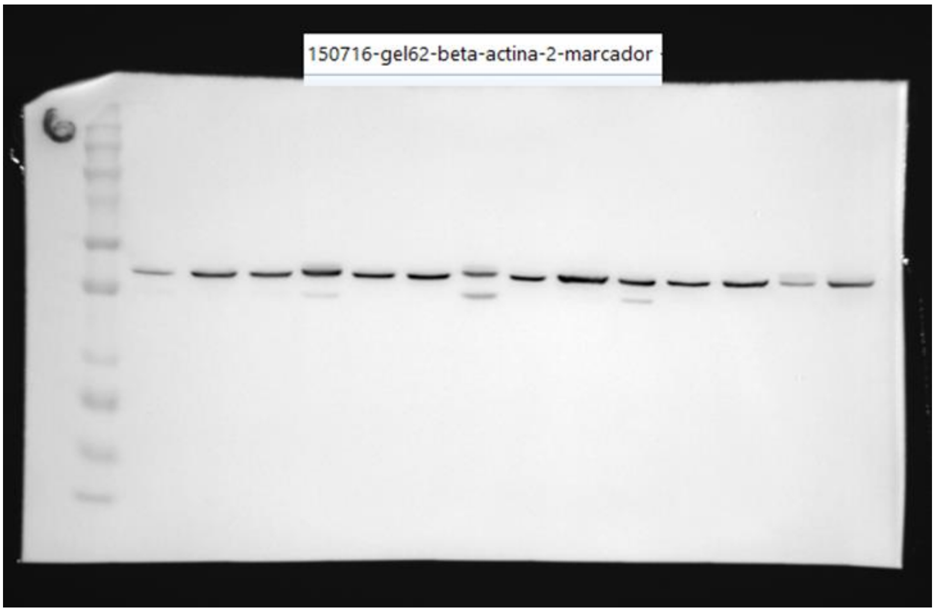

150716-gel6.2-
beta-actina-2-WB

Sample	lane
CG8-0J	1
DG8-0J	2
DG3-20J	3
CT2-0J	4
DG5-0J	5
DG4-20J	6
CN12-0J	7
DG2-0J	8
DG1-20J	9
CN11-0J	10
DN3-0J	11
DG7-20J	12
CN10-0J	13
DN8-0J	14

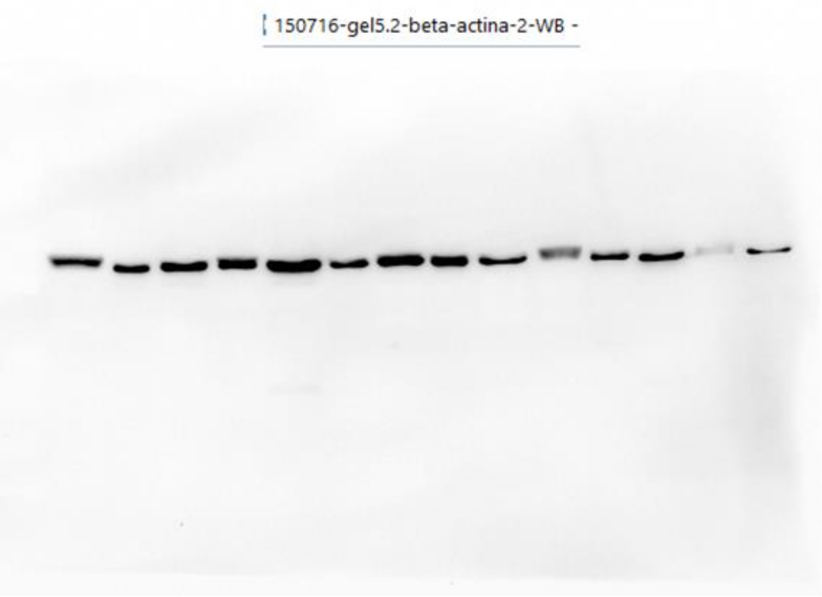

150716-gel5.2-
beta-actina-2-WB

Sample	lane
CT3-0J	1
DT16-0J	2
DT1-20J	3
CT4-0J	4
DT14-0J	5
DT4-20J	6
CE4-0J	7
DT6-0J	8
DE3-20J	9
CT5-0J	10
DT11-0J	11
DT8-20J	12
CT6-0J	13
DT9-0J	14
