# Supplementary material for: Early effect of laser irradiation in signaling pathways of diabetic rat submandibular salivary glands
Source: PLoS One. 2020 Aug 4;15(8):e0236727. doi: 10.1371/journal.pone.0236727 (PMC7402516; doi:10.1371/journal.pone.0236727)
Supplement: S1 Raw file — (ZIP) [file pone.0236727.s004.zip › 070620-WB Phospho NFkB.pdf]

WB Phospho NFkB 65 Kda

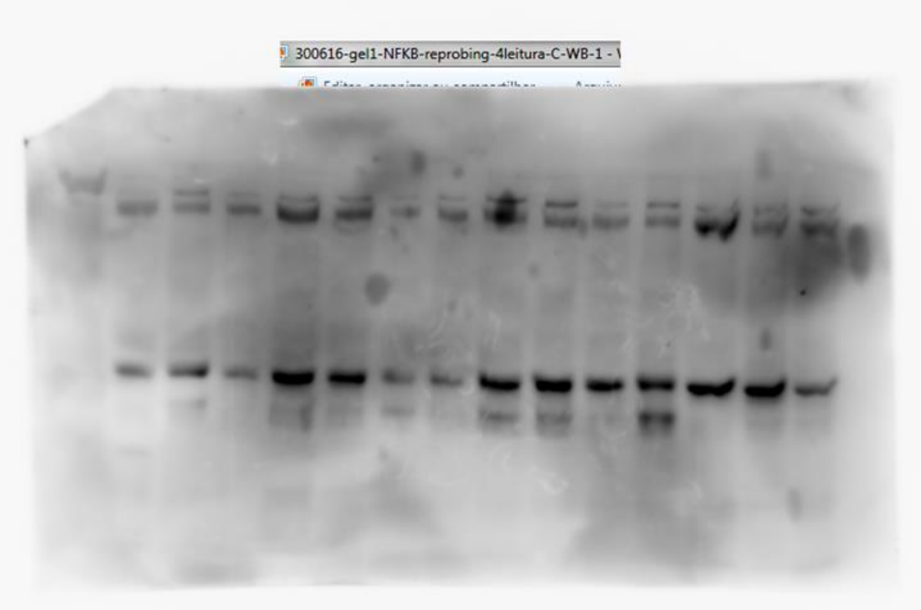

300616-Gel1-NFkB-reprobing-aliqC-4leitura-Wb-1

Sample	lane
CG8-0J	1
DG8-0J	2
DG3-20J	3
CN9-0J	4
DG5-0J	5
DG4-20J	6
CN7-0J	7
DG2-0J	8
DG1-20J	9
DN3-0J	10
DG7-20J	11
CN8-0J	12
DN8-0J	13
DG12-20J	14

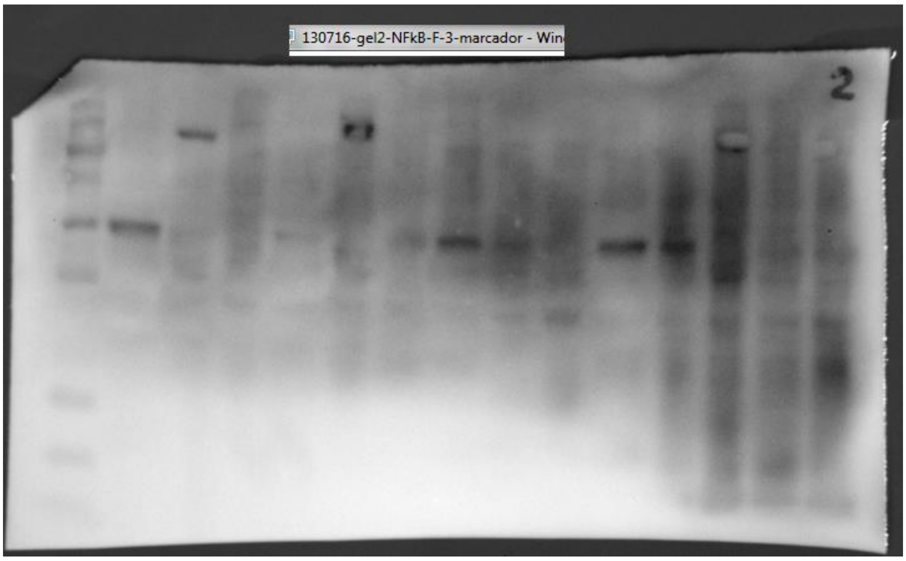

Sample	lane
CT3-0J	1
DT1-20J	2
CT4-0J	3
DT14-0J	4
DT13-20J	5
CE4-0J	6
DT6-0J	7
DE3-20J	8
CT5-0J	9
DT11-0J	10
DT8-20J	11
CT6-0J	12
DT9-0J	13
DT4-20J	14
